# Mechanism of cargo recognition by retromer-linked SNX-BAR proteins

**DOI:** 10.1371/journal.pbio.3000631

**Published:** 2020-03-09

**Authors:** Xin Yong, Lin Zhao, Wankun Deng, Hongbin Sun, Xue Zhou, Lejiao Mao, Wenfeng Hu, Xiaofei Shen, Qingxiang Sun, Daniel D. Billadeau, Yu Xue, Da Jia

**Affiliations:** 1 Key Laboratory of Birth Defects and Related Diseases of Women and Children, Department of Paediatrics, West China Second University Hospital, State Key Laboratory of Biotherapy and Collaborative Innovation Center of Biotherapy, Sichuan University, Chengdu, China; 2 Department of Bioinformatics & Systems Biology, Key Laboratory of Molecular Biophysics of Ministry of Education, College of Life Science and Technology, Huazhong University of Science and Technology, Wuhan, China; 3 School of Food and Biological Engineering, Zhengzhou University of Light Industry, Zhengzhou, China; 4 Division of Oncology Research and Schulze Center for Novel Therapeutics, Mayo Clinic, Rochester, Minnesota, United States of America; Institute of Basic Medical Sciences, NORWAY

## Abstract

Endocytic recycling of internalized transmembrane proteins is essential for many important physiological processes. Recent studies have revealed that retromer-related Sorting Nexin family (SNX)–Bin/Amphiphysin/Rvs (BAR) proteins can directly recognize cargoes like cation-independent mannose 6-phosphate receptor (CI-MPR) and Insulin-like growth factor 1 receptor (IGF1R); however, it remains poorly understood how SNX-BARs select specific cargo proteins and whether they recognize additional ligands. Here, we discovered that the binding between SNX-BARs and CI-MPR or IGF1R is mediated by the phox-homology (PX) domain of SNX5 or SNX6 and a bipartite motif, termed SNX-BAR-binding motif (SBM), in the cargoes. Using this motif, we identified over 70 putative SNX-BAR ligands, many of which play critical roles in apoptosis, cell adhesion, signal transduction, or metabolite homeostasis. Remarkably, SNX-BARs could cooperate with both SNX27 and retromer in the recycling of ligands encompassing the SBM, PDZ-binding motif, or both motifs. Overall, our studies establish that SNX-BARs function as a direct cargo-selecting module for a large set of transmembrane proteins transiting the endosome, in addition to their roles in phospholipid recognition and biogenesis of tubular structures.

## Abstract 2

Internalized transmembrane proteins can be recognized by specific protein complexes and diverted away from the degradation process. This study identifies a new sorting motif recognized by retromer-linked SNX-BAR proteins and reveals a large repertoire of potential cargoes recycled by the SNX-BAR proteins.

## Introduction

Cell surface integral-membrane proteins and their ligands taken up during endocytosis are either sent to lysosome for degradation or subjected to recycling back to the plasma membrane directly or via the trans-Golgi network (TGN) [1–3]. These processes, known as endosomal protein sorting, are not only essential to maintain plasma membrane homeostasis but also indispensable for proper cellular responses to external signals [1–3]. Emphasizing the importance of endosomal protein sorting is the observation that genetic defects in these processes have been linked with a variety of human diseases including Alzheimer’s disease, Parkinson’s disease, cancer, and diabetes [4, 5].

Key protein machineries important for the sequence-dependent recycling include the evolutionarily conserved retromer complex (vacuolar protein sorting 35 [VPS35]/VPS26/VPS29 in higher eukaryotes) [6–8], the recently discovered retriever [9], the WASH complex [10–12], and members of the Sorting Nexin family (SNX) [13–15]. A subset of SNX proteins possessing a Bin/Amphiphysin/Rvs (BAR) domain, in addition to the phox-homology (PX) domain, have been linked with the retromer complex. The retromer-related SNX-BAR proteins (referred as SNX-BARs herein) SNX1, SNX2, SNX5, SNX6, and SNX32 form heterodimeric complexes and are critical for both endosome–to–plasma membrane recycling and endosome-to-TGN retrieval [16, 17]. Current models suggest that SNX-BARs promote the endosome–to–plasma membrane recycling via associating with SNX27 and retromer, with the PDZ domain of SNX27 as the predominant cargo-recognition module [14]. For endosome-to-TGN trafficking, one of the best-characterized cargoes is cation-independent mannose 6-phosphate receptor (CI-MPR), which is necessary to deliver newly synthesized lysosomal hydrolases to the endosomal lumen and thus critical for lysosomal function [18]. However, previous studies have provided contradictory models regarding the role of retromer and SNX-BARs in the endosome-to-TGN retrieval of CI-MPR. Work from many different labs has initially supported the idea that retromer is necessary for the retrieval of CI-MPR, likely through a direct association with its cytoplasmic tail, in particular, the hydrophobic WLM motif [19–21]. However, recent work by Cullen and Steinberg has provided a very different model, in which SNX-BARs, but not retromer, associate with the cytoplasmic tail of CI-MPR and mediate its trafficking [22, 23].

The importance of endosomal sorting pathways for cellular homeostasis and functions is further highlighted by the findings that many viral and bacterial pathogens target these pathways for survival and growth [24]. For instance, whereas retromer activity restricts intracellular growth of the bacteria *Chlamydia*, *Chlamydia* counteracts the host activity through a direct binding between its effector protein IncE and SNX5/SNX6 [25]. Ectopic IncE production disrupts CI-MPR endosomal trafficking, likely by competing with CI-MPR for SNX5 binding [26, 27]. However, it remains poorly understood how SNX-BARs selectively bind to CI-MPR or other cargoes.

Here, we set out to define the mechanisms by which SNX-BARs recognize their ligands. We found a conserved SNX-BAR-binding motif (SBM) in CI-MPR and other cargoes, which consists of the WLM motif or its equivalents, and a hydrophobic stretch localized upstream of the WLM motif. In multiple ligands, the SBM is necessary for the binding to SNX-BARs and subsequent trafficking to the plasma membrane or TGN. In silico analyses of the human proteome revealed over 70 putative SNX-BAR cargoes, suggesting that SNX-BARs have broad functions in receptor trafficking beyond mere tubulation of membranes.

## Results

### SNX-BARs, but not retromer or the SNX3-retromer complex, directly interact with CI-MPR

**Fig 1:**
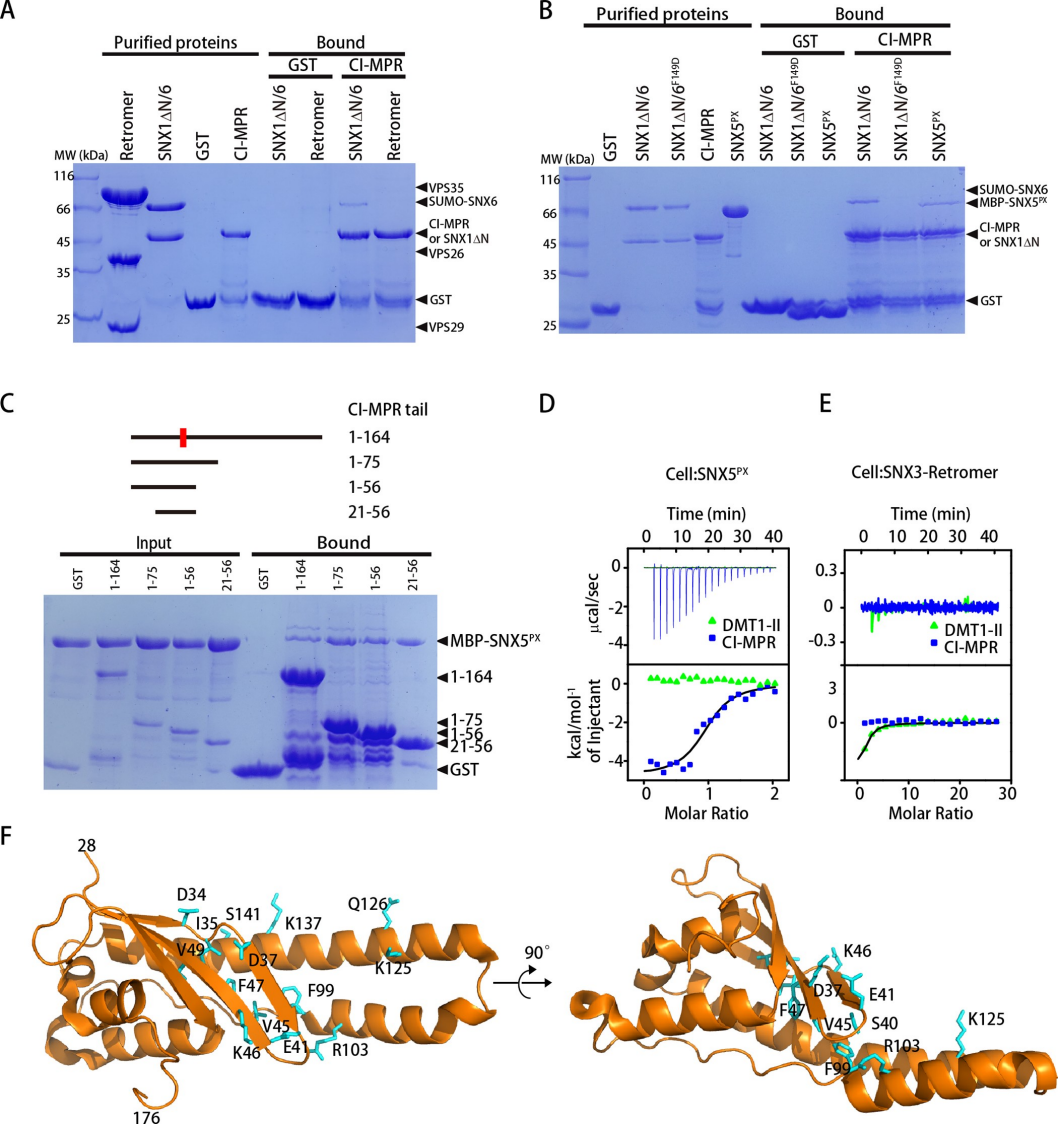
CI-MPR directly interacts with the PX domain of SNX5 or SNX6, but not retromer or the SNX3-retromer complex. (A) GST-CI-MPR (aa1–164) or GST pull-down of purified retromer VPS35/VPS26/VPS29, and SNX1ΔN/SNX6. Shown is a Coomassie blue–stained SDS-PAGE gel of purified proteins used (left) and bound samples (right). The SUMO fusion of SNX6 was uncleaved in order to distinguish the SNX6 protein bands from those of SNX1ΔN or GST-CI-MPR. (B) GST-CI-MPR or GST pull-down of purified SNX1ΔN/SNX6, SNX1ΔN/SNX6-F149D, MBP-SNX5^PX^. Shown is a Coomassie blue–stained SDS-PAGE gel of purified proteins used (left) and bound samples (right). (C) GST-CI-MPR pull-down of purified MBP-SNX5^PX^. Shown are the CI-MPR constructs used (top) and a Coomassie blue–stained gel of purified proteins used and bound samples (bottom). (D) Isothermal titration calorimetry of CI-MPR (aa21–48, blue) or DMT1-II (green) titrated into SNX5^PX^ in a buffer containing 100 mM Hepes (pH 7.5), 300 mM NaCl, 2 mM βME at 25°C. Top and bottom panels show raw and integrated heat from injections, respectively. The black curve in the bottom panel represents a fit of the integrated data to a single-site binding model. Experiments were triplicated, and the numerical data are included in S1 Data. (E) Isothermal titration calorimetry of CI-MPR (aa21–48, blue) or DMT1-II (green) titrated into in the SNX3-retromer complex under the conditions identical to (D). Top and bottom panels show raw and integrated heat from injections, respectively. The black curve in the bottom panel represents a fit of the integrated data to a single-site binding model. (F) Identification of the CI-MPR-binding residues of SNX5^PX^ by NMR. Residues showing significant change of chemical shift or line broadening with the addition of the CI-MPR peptide (aa21–48) are displayed as sticks. CI-MPR, cation-independent mannose 6-phosphate receptor; DMT1-II, divalent metal transporter 1; GST, glutathione-S-transferase; MBP, maltose binding protein; MW, molecular weight; NMR, nuclear magnetic resonance; PX, phox-homology; SNX, Sorting Nexin family; SNX5^PX^, PX domain of SNX5; VPS, vacuolar protein sorting.

Previous studies lead to two distinct models of CI-MPR endosomal trafficking, in which CI-MPR is recycled through direct engagement with retromer or SNX-BARs, respectively. To begin to test these models, we examined whether the binding between the CI-MPR tail and either retromer or the SNX1/SNX6 complex was direct [28]. The glutathione-S-transferase (GST)-CI-MPR tail specifically retained SNX1/SNX6, but not retromer (Fig 1A). Importantly, a single amino acid mutation within the PX domain of SNX6 (SNX1/SNX6-F149D) completely disrupted the association (Fig 1B). Furthermore, the PX domain of SNX5 (SNX5^PX^) was sufficient to interact with the tail, suggesting that the dimerization between SNX1/2 and SNX5/6/32 is not necessary for cargo engagement (Fig 1B). The interaction between CI-MPR and SNX5 can be further mapped to a short segment of CI-MPR (aa21–56 or aa21–48), which harbors the WLM motif (Fig 1C and 1D).

As a recent study suggested that retromer functioned together with SNX3 to promote the trafficking of a subset of CI-MPR [29], we also examined the interaction between CI-MPR and the SNX3-retromer complex. CI-MPR robustly retained SNX1/SNX5 but failed to efficiently pull down the SNX3/VPS35/VPS26 complex (S1A Fig). Quantitative measurement using isothermal titration calorimetry (ITC) [30, 31] revealed that CI-MPR bound to SNX5^PX^, with a dissociation constant (K_d_) value of approximately 18 μM; in contrast, we did not detect any obvious binding between CI-MPR and the SNX3-retromer under our experimental conditions (K_d_ > 400 μM) (Fig 1D and 1E). As a control, divalent metal transporter 1 (DMT1-II), an established cargo of retromer and SNX3, bound to the SNX3-retromer complex with an affinity of K_d_ ≈ 110 μM, close to a reported value under similar assay conditions (127 μM) [13, 32]. Interestingly, although DMT1-II bears a hydrophobic motif (YLL) similar to the WLM motif of CI-MPR, DMT-II did not show apparent binding toward SNX5^PX^, suggesting that additional elements within the CI-MPR tail contribute to the binding (Fig 1D). Finally, ITC experiments revealed that SNX6^PX^ and SNX32^PX^ bind to CI-MPR similarly (K_d_ of 18 μM, 36 μM, and 18 μM for SNX5^PX^, SNX6^PX^, and SNX32^PX^, respectively) (S1B Fig). Interestingly, sequence alignment of members of the SNX family indicates that SNX5/6/32, but not other proteins, contain a long helical hairpin (α2 and α3) (S2 Fig).

To identify the essential residues within SNX5^PX^ that interact with CI-MPR, we used nuclear magnetic resonance (NMR) spectroscopy to examine the perturbations in SNX5^PX^ upon the addition of the CI-MPR peptide (Fig 1E and S3A Fig). SNX5^PX^ labeled with ^15^N/^13^C was produced, and backbone assignments were obtained similar to a published study [33]. To identify the CI-MPR-binding surface, residues displaying significant change of chemical shift on the binding of CI-MPR were mapped onto the SNX5^PX^ crystal structure (PDB: 3HPC). Remarkably, the majority of residues with peaks showing significant chemical shifts or line broadening are found within the β1–β2 of SNX5 PX domain (D34, I35, D37, S40, E41, V45, K46, F47, V49) or the long α-helical hairpin (F99, G101, R103, K125, Q126, S141, K137, T142), regions that are involved in contacting *Chlamydia trachomatis* effector protein IncE [26, 27, 34]. To further identify the SNX5^PX^ residues that interact with CI-MPR, we mutated five amino acids within the above regions that have been shown to directly contact IncE and tested their binding toward CI-MPR. Mutation of three residues within SNX5^PX^ (Y132D, Y136D, E144A) nearly abolished the interaction with CI-MPR, and the E129A mutant also diminished the interaction (S3B Fig). Furthermore, the binding between CI-MPR and SNX5^PX^ could be effectively competed by as low as equivalent molar ratio of IncE, consistent with the finding that the binding affinity between IncE and SNX5^PX^ is about one order of magnitude higher (K_d_ = 0.5 to approximately 1 μM for IncE and approximately 18 μM for CI-MPR) [26, 27, 34] (S3C Fig). Thus, SNX5 likely interacts with CI-MPR in a manner analogous to that of IncE.

### Identification of the SBM

**Fig 2:**
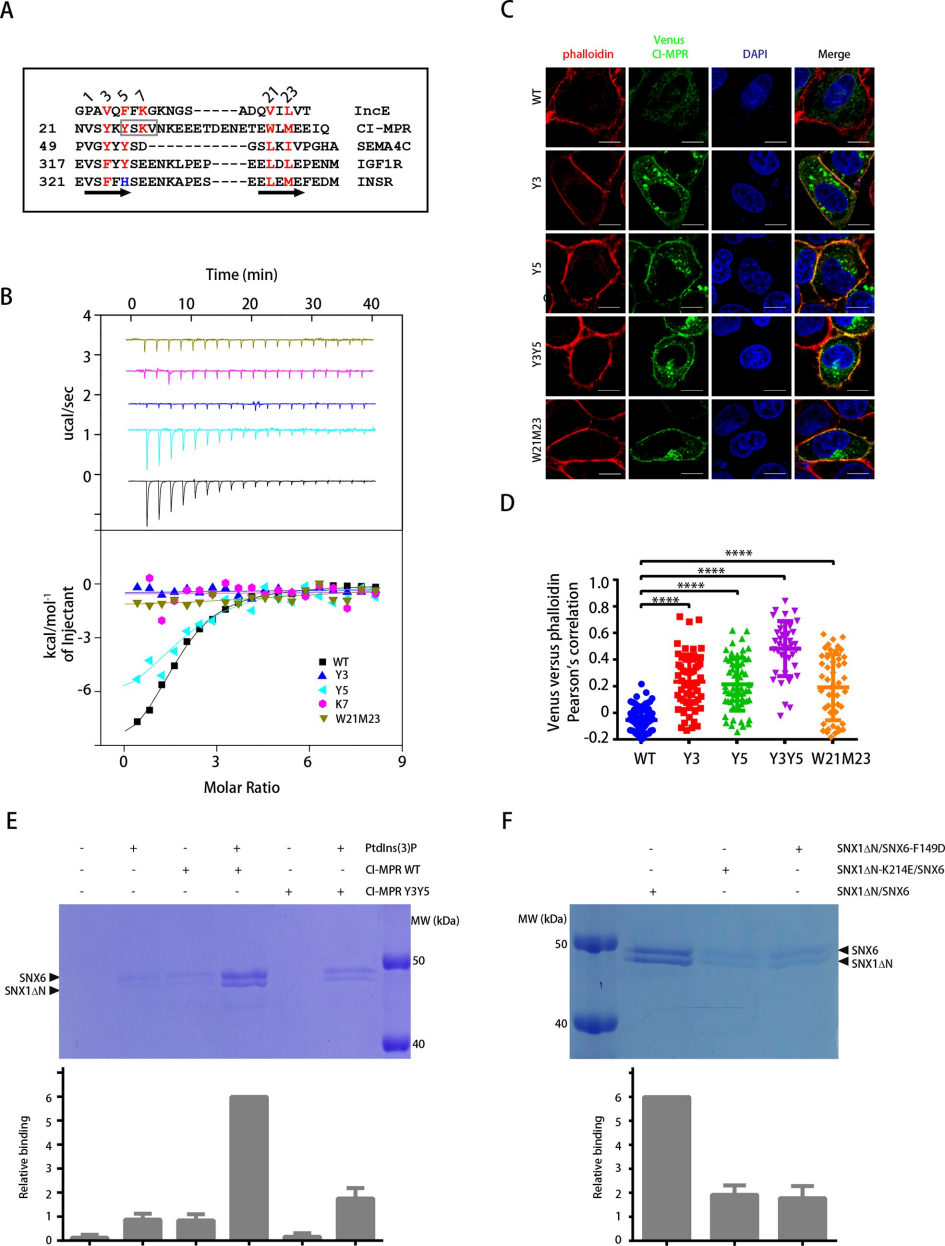
Cooperative binding of an SBM within the CI-MPR tail and PtdIns(3)P recruits SNX-BAR to a membrane. (A) Sequence alignment of the known SNX-BAR interactors. Red color indicates residues conserved among the interactors; blue color indicates a His residue with the third position of INSR, which does not bind to the SNX-BAR proteins. The number before the first residue of each sequence indicates the position of this residue in the cytoplasmic tail. Secondary structure of IncE is listed below the sequences. The AP-2 binding motif within CI-MPR (YSKV) is indicated with a box. (B) Isothermal titration calorimetry of CI-MPR (aa21–48) WT or mutants titrated into SNX5^PX^ in a buffer containing 100 mM Hepes (pH 7.5), 300 mM NaCl, 2 mM βME at 25°C. Raw data are listed at the top; integrated normalized data are listed at the bottom. Colors indicate the peptides used in the experiment. Experiments were triplicated, and the numerical data are included in S1 Data. (C) Steady-state localization of Venus-CI-MPR WT and mutants in HeLa cells. Cells were transfected with Venus-CI-MPR constructs (green) and stained with phalloidin (red). Representative images are shown. Scale bar: 10 μm. (D) Colocalization of Venus-CI-MPR and phalloidin in cells in D. Each dot represents Pearson’s correlation coefficients (r) from one cell. Experiments were triplicated, and the numerical data are included in S1 Data. ****P < 0.0001. *P* values were compared with WT using one-way ANOVA, Tukey's multiple comparisons test, throughout the paper unless otherwise indicated. (E) Binding of the SNX1ΔN/SNX6 complex to liposomes in a liposome flotation assay. Liposomes with or without PtdIns(3)P were incubated with purified SNX1ΔN (aa140–C)/SNX6 complex in the presence or absence of His-CI-MPR tail and then centrifuged in a Histodenz gradient. Samples that floated to the top of the gradient were collected and subjected to SDS-PAGE. Gel image from one of three independent experiments is shown. Total intensity of SNX1 and SNX6 protein bands was quantified using ImageJ and normalized to the level of sample 4 in each experiment. Error bars represent standard deviation. The numerical data are included in S1 Data. (F) Binding of the SNX1ΔN/SNX6 complex WT or mutants to liposomes in a liposome flotation assay. Liposomes with PtdIns(3)P were incubated with purified SNX1ΔN (aa140–C)/SNX6 complex WT or mutants in the presence of His-CI-MPR tail. Shown is the CBB-stained SDS-PAGE for samples floated to the top of the gradient. Intensity of protein bands is normalized to the level of WT proteins. Statistic data represent the results from *n* = 3 independent experiments and are expressed as mean ± SD. The numerical data are included in S1 Data. AP-2, adaptor protein complex 2; BAR, Bin/Amphiphysin/Rvs; CBB, Coomassie brilliant blue; CI-MPR, cation-independent mannose 6-phosphate receptor; IGF1R, Insulin-like growth factor 1 receptor; INSR, insulin receptor; MW, molecular weight; PtdIns(3)P, phosphatidylinositol 3-phosphate; SBM, SNX-BAR-binding motif; SEMA4C, semaphorin 4C; SNX, Sorting Nexin family; WT, wild type.

Sequence alignment between IncE, CI-MPR, and two other known SNX-BAR-binding proteins, semaphorin 4C (SEMA4C) [22] and Insulin-like growth factor 1 receptor (IGF1R), revealed a conserved bipartite motif, which we have termed the SBM (Fig 2A). The WLM residues of CI-MPR fit a ΦXΦ sequence consensus, in which Φ and X symbolize hydrophobic and any amino acids, respectively. About 3–15 amino acids N-terminal to the WLM motif, we identified another highly conserved motif, which fit a consensus ΨX[FY]X[RK] or ΩX[FY], in which Ψ and Ω represent aliphatic and aromatic hydrophobic amino acids, respectively. In the crystal structure of SNX5^PX^ in complex with IncE, IncE forms two antiparallel strands connected by a flexible linker. Several hydrophobic residues of IncE insert into hydrophobic pockets on the surface of SNX5^PX^. In the following discussion, V22 in the CI-MPR tail or its equivalent is designated position 1, and W of the WLM motif or its equivalent as 21, and other residues are numbered in accordance with this designation (Fig 2A).

To confirm the importance of the above consensus, we converted multiple residues of CI-MPR to alanine and tested their binding to SNX5^PX^ using pull-down and ITC assays (Fig 2B and S3A Fig). We also included a mutation deleting five residues from the loop connecting the two hydrophobic stretches (Δloop5). All alanine substitutions within the consensus dramatically reduced the binding to SNX5^PX^, and the V1 mutant also displayed a slightly weaker binding (S4A Fig). In contrast, the Δloop5 mutant still efficiently bound to the SNX5^PX^, confirming that the two hydrophobic stretches within SBM, but not the residues connecting them, are most essential for the interaction (S4A Fig). Quantitative analysis using ITC further indicated that the Y3, K7, W21M23 mutants nearly abolished the interaction, whereas the Y5 mutant decreased the affinity about 2-fold (K_d_ of 18 μM and 36 μM CI-MPR WT and Y5, respectively) (Fig 2B). Further analysis demonstrated that the length for a linker (the distance between K5 and W21) optimal to interact with SNX5 is approximately 5 residues, as Δloop8 displayed a highest affinity (K_d_ of 18 μM, 10 μM, 2.5 μM, and 25 μM for CI-MPR WT, Δloop5, Δloop8, Δloop10, respectively) (S4B and S4C Fig).

In order to further test the functional roles of the CI-MPR residues contacting SNX-BARs, we made use of an assay developed by Waguri and colleagues and extensively used by Simonetti and colleagues, in which the cytoplasmic tail of CI-MPR was linked to an endosome- and Golgi-targeting signal peptide and a fluorescent tag (signal peptide–Venus-Transmembrane domain–CI-MPR, referred as Venus-CI-MPR) [23, 35]. The wild-type (WT) chimera construct showed a predominately cytosolic localization (Fig 2C). Incorporation of single or double mutations (Y3, Y5, Y3Y5, W21M23) into the Venus-CI-MPR construct significantly altered the steady-state distribution of the CI-MPR, shifting from cytosol to peripheral localization, as determined by the colocalization of CI-MPR and phalloidin (Fig 2C and 2D).

Since the PX domains of SNX1/2 and SNX5/6 interact with phosphatidylinositol 3-phosphate (PtdIns[3]P) and the CI-MPR tail, respectively, we tested whether PtdIns(3)P and CI-MPR might cooperate to recruit the SNX-BAR complex to membrane using a liposome flotation assay (Fig 2E and 2F). Although either PtdIns(3)P or liposome-bound CI-MPR tail was sufficient to recruit the SNX1/SNX6 complex to the liposome, the SNX1/SNX6 recruitment was substantially increased (>6-fold) when both PtdIns(3)P and CI-MPR were present (Fig 2E, lane 4 versus lane 2 or 3). Importantly, the CI-MPR mutant Y3Y5 was ineffective in promoting SNX1/SNX6 to liposomes, thus emphasizing the importance of the SBM in mediating the recruitment (Fig 2E, lane 3 versus 5, or lane 4 versus 6). To further investigate the recognition of PtdIns(3)P and CI-MPR for the membrane recruitment of SNX-BAR, we introduced two mutants within the SNX1/SNX6 complex, which abolished the interaction with PtdIns(3)P (SNX1-K214E) [36] or CI-MPR (SNX6-F149D), respectively. Both mutations led to a substantially decreased amount of the SNX-BAR complex recruited to liposomes (Fig 2F). Therefore, the cooperation between sequence-specific binding of membrane-bound cargoes and PtdIns(3)P recognition promotes the membrane recruitment of SNX-BAR.

The insulin receptor (INSR) and IGF1R share many biological activities, including the vast majority of downstream effectors. Interestingly, although the cytoplasmic tails of these two proteins have an amino acid sequence identity of 72%, IGF1R, but not INSR, is recycled by the SNX-BARs [22]. A carful inspection of sequence alignments revealed that IGF1R retains a complete SBM, whereas INSR contains an “H” instead of the conserved “Y” or “F” in position 3 (Fig 2A). Indeed, substitution of Y in position 5 with H (Y5H) or double alanine substitution (F3Y5) dramatically reduced or abolished the binding between IGF1R and SNX5^PX^ (S4D Fig). Conversely, an H5Y substitution allowed INSR to gain interaction with SNX5^PX^ (S4D Fig). Overall, these data established that both hydrophobic stretches of the SBM in multiple proteins are necessary to interact with SNX-BARs.

### Dual recognition of SEMA4C by SNX-BAR and SNX27

**Fig 3:**
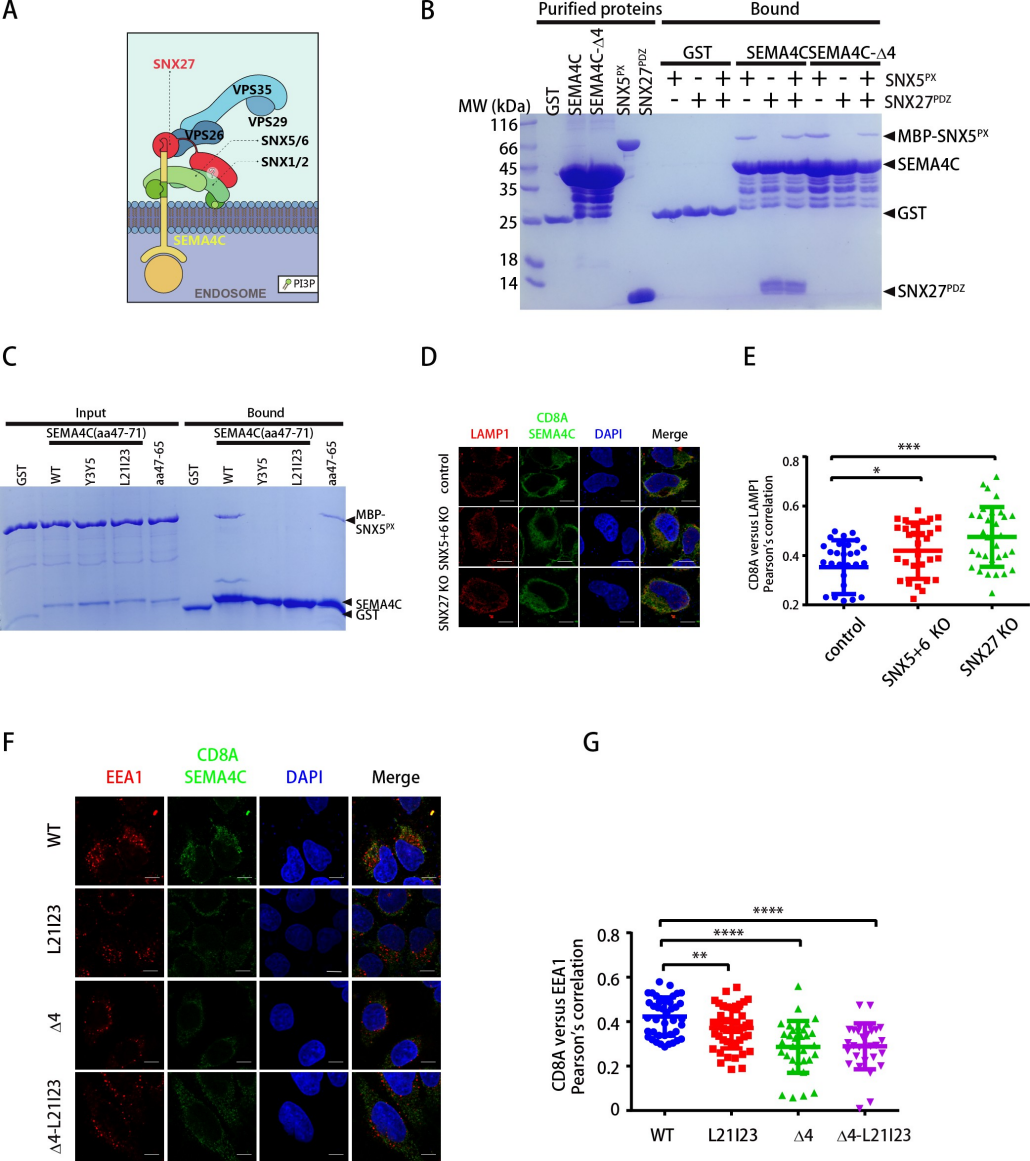
Dual recognition by SNX-BAR and SNX27 mediates transport of SEMA4C. (A) Model showing dual recognition of SEMA4C by SNX-BAR and the SNX27-retromer complex. The PX domains of SNX1/SNX2 and SNX5/SNX6 interacts with membrane-bound PtdIns(3)P and ligands like SEMA4C, respectively. SNX27 harbors a PDZ domain, followed by a PX and FERM domain. Its PDZ and FERM domain bind to the PDZbm within SEMA4C, and SNX1/SNX2, respectively. For simplicity, the PX domain of SNX27 is omitted. (B) GST, GST-SEMA4C-tail (aa1–149), or GST-SEMA4C-Δ4 (aa1–145) pull-down of purified MBP-SNX5^PX^, or SNX27^PDZ^, or the mixture of MBP-SNX5^PX^ and SNX27^PDZ^. Shown are Coomassie blue–stained SDS-PAGE gels of purified proteins used (left) and bound samples (right). (C) GST, GST-SEMA4C (aa47–71) WT or mutants (Y3Y5, L21I23), or GST-SEMA4C (aa47–65) pull-down of purified MBP-SNX5^PX^. Shown are Coomassie blue–stained SDS-PAGE gels of purified proteins used (left) and bound samples (right). (D) Control, SNX1+2-KO, SNX5+6-KO, and SNX27-KO HeLa cells were transiently transfected with plasmids encoding CD8A-SEMA4C. Cells were incubated with monoclonal anti-human CD8A antibody on ice for 30 min. Unbound antibodies were removed, and the internalization of antibody-bound CD8A was carried out in DMEM at 37°C for 3 h. The internalized CD8A–antibody was detected using Alexa-488 secondary antibodies, with lysosomes stained with LAMP1 (red). Scale bar: 10 μm. (E) Quantification of CD8A/LAMP1 colocalization in cells in D. Each dot represents Pearson’s correlation coefficients from one cell. *N* = 3 independent experiments. *P* values were calculated using one-way ANOVA, Tukey's multiple comparisons test. *P < 0.05; ***P < 0.001. The numerical data are included in S1 Data. (F) HeLa cells were transiently transfected with plasmids encoding CD8A-SEMA4C WT, L21I23, Δ4, and Δ4-L21I23. Cells were incubated with monoclonal anti-human CD8A antibody on ice for 30 min. Unbound antibodies were removed, and the internalization of antibody-bound CD8A was carried out in DMEM at 37°C for 1 h. The internalized CD8A–antibody was detected using Alexa-488 secondary antibodies, with early endosomes stained with EEA1 (red). Scale bar: 10 μm. (G) Quantification of CD8A/EEA1 colocalization in cells in F. Each dot represents Pearson’s correlation coefficients from one cell. *N* = 3 independent experiments. *P* values were calculated using one-way ANOVA, Tukey's multiple comparisons test. **P < 0.01; ****P < 0.0001. The numerical data are included in S1 Data. BAR, Bin/Amphiphysin/Rvs; DMEM, Dulbecco’s modified Eagles medium; EEA1, early endosome antigen 1; GST, glutathione-S-transferase; KO, knockout; LAMP1, lysosomal-associated membrane protein 1; MBP, maltose binding protein; PDZbm, PDZ-binding motif; PtdIns(3)P, phosphatidylinositol 3-phosphate; PX, phox-homology; SEMA4C, semaphorin 4C; SNX, Sorting Nexin family; SNX5^PX^, PX domain of SNX5; WT, wild type; VPS, vacuolar protein sorting.

SEMA4C is a single-pass membrane protein belonging to the semaphorin family and plays a role in the neural system development. SEMA4C is identified as a potential binding partner of the SNX-BARs, although the functional significance of this interaction remains to be explored [22]. We found that the cytoplasmic tail of SEMA4C harbors a PDZ-binding motif (PDZbm) at its C terminus, in addition to the SBM in the amino acids 47–71 (Fig 3A). Indeed, whereas the recombinant SEMA4C tail bound to both SNX5^PX^ and SNX27^PDZ^, a deletion of the last four amino acids (SEMA4C-Δ4) retained the interaction with SNX5^PX^ but specifically lost the SNX27^PDZ^ association (Fig 3B). In cells, SEMA4C WT effectively precipitated SNX1, SNX5, and SNX27; conversely, SEMA4C-Δ4 lost its interaction with not only SNX27 but also SNX1, consistent with the previous finding that SNX27 specifically associates with SNX1/2 [14] (S5A Fig). GST pull-down using purified protein revealed that truncated forms of SEMA4C (aa47–71 or aa47–65), both including a complete SBM, were able to interact with SNX5^PX^ (Fig 3C and S5B Fig). Consistent with our data of CI-MPR and IGF1R, alanine substitutions in the positions 3 and 5 or 21 and 23 (Y3Y5 and L21I23) of SEMA4C resulted in a dramatic loss of the binding to the SNX-BARs (Fig 3C, S5C and S5D Fig). Thus, SEMA4C can bind to SNX-BAR and SNX27 independently and simultaneously, which could promote the assembly of a SNX-BAR-SNX27-retromer supercomplex for endosome–to–plasma membrane trafficking.

To investigate the functional significance of the interaction between SEMA4C and SNX-BARs, we engineered a CD8A-SEMA4C chimera and performed uptake assays using an antibody against the extracellular region of CD8A [20, 21]. Using CRISPR-Cas9 technology, we generated knockout (KO) cells that are depleted for both SNX5 and SNX6 (SNX5+6 KO) or SNX27 (SNX27 KO) (S6 Fig). Immunofluorescence analysis of SEMA4C localization showed that the loss of SNX5+6 or SNX27 did not impair the internalization of CD8A-SEMA4C, as determined by the colocalization of SEMA4C and phalloidin 30 min after internalization (S6C and S6D Fig). In contrast, the internalized SEMA4C failed to recycle properly and entered the lysosomal degradation pathway in both SNX5+6 KO and SNX27 KO cells, as defined by the increasing colocalization of SEMA4C and the marker lysosomal-associated membrane protein 1 (LAMP1) (Fig 3D and 3E). Accordingly, SEMA4C, together with CI-MPR and TNF-related apoptosis-inducing ligand (TRAIL) receptor 1 (TRAILR1) (see later discussion), were lost from the cell surface in both cells (S6A and S6B Fig). In contrast, the protein level of Integrin α5, a known cargo of SNX17 and retriever, remained unchanged in SNX5+6 KO and SNX27 KO cells (S6A and S6B Fig).

To determine residues of SEMA4C critical for its endocytic recycling, we introduced mutations in either with the SBM (L21I24) or the PDZbm (Δ4) or both (Δ4-L21I23). All the mutants displayed a decreased colocalization with early endosome antigen 1 (EEA1), consistent with the fact that both SBM and PDZbm of SEMA4C are required for its recycling (Fig 3F and 3G). Finally, to test whether the redistribution to cytosol could lead to enhanced degradation of SEMA4C, we compared protein levels of Venus-SEMA4C WT and mutants in cells treated with the ribosomal inhibitor cycloheximide (S6E and S6F Fig). A double mutant (Δ4-L21I23) that disrupted the interaction with both SNX-BARs and SNX27 displayed enhanced degradation kinetics relative to the WT protein (S6E and S6F Fig). Taken together, SEMA4C is likely to be recognized and recycled by both SNX-BARs and SNX27.

### Discovery and verification of additional SNX-BAR ligands

**Fig 4:**
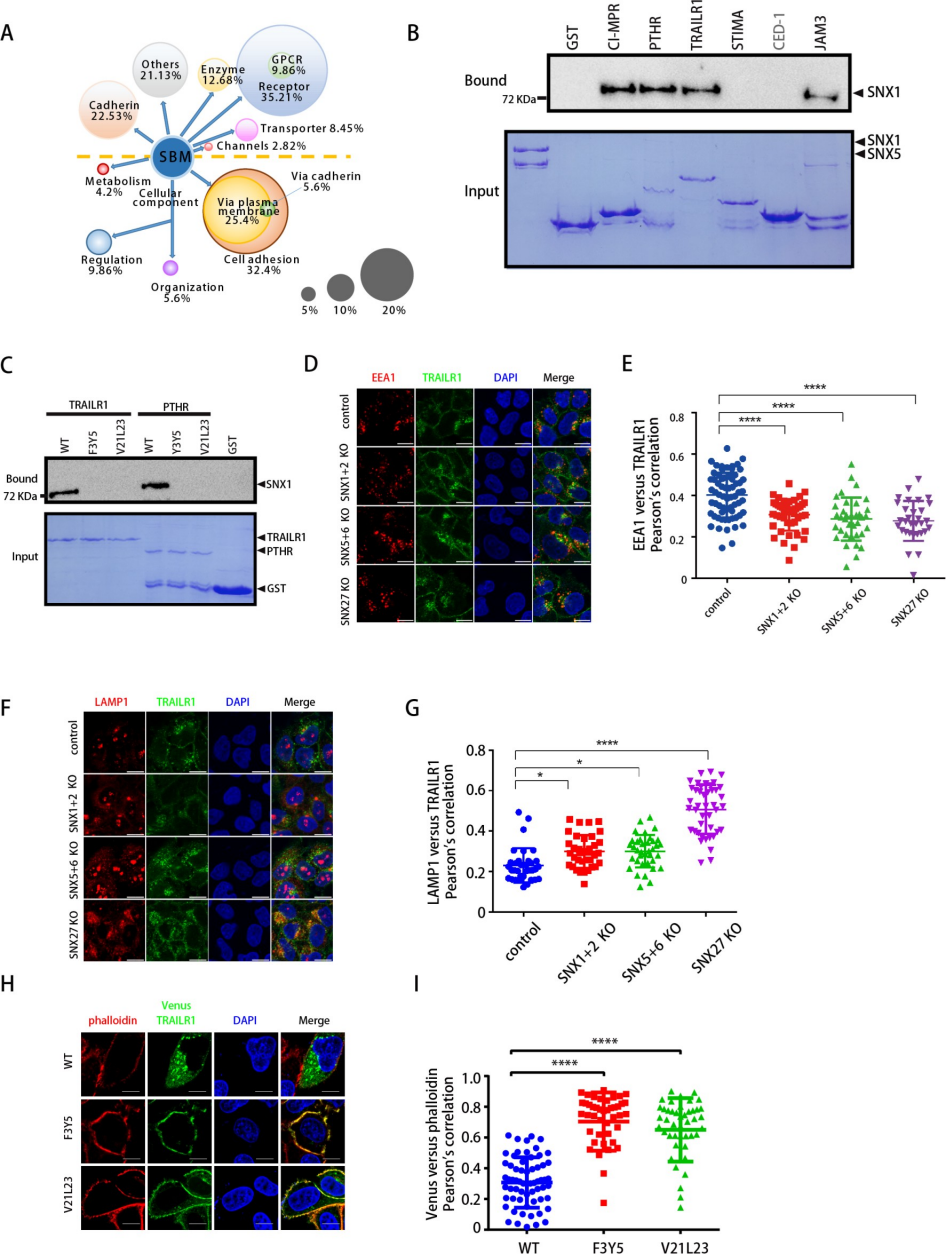
Identification and experimental verification of additional cargo proteins of SNX-BARs. (A) Schematic representation of different functions of SBM-containing proteins. Seventy-one human SBM-containing proteins were manually classified into six categories according to literature, including receptor (GPCR and non-GPCR), enzyme, channel, transporter, cadherin, and others (top), or were analyzed by GO and classified by their biological processes. (B) GST-CI-MPR, PTHR, TRAILR1, STIMA, CED-1, JAM3, or GST pull-down of purified MBP-SNX1/His_6_-sumo-SNX5. Shown are a Coomassie blue–stained SDS-PAGE gel of purified proteins (bottom) and immunoblot using anti-MBP antibody for the same sample (top). CED-1 does not have a SBM and is labeled as gray. (C) GST-TRAILR1 and GST-PTHR WT or mutants pull-down of purified MBP-SNX1//His_6_-sumo-SNX5. Shown are a Coomassie blue–stained SDS-PAGE gel of purified proteins (bottom) and immunoblot using anti-MBP antibody for the same sample (top). (D) Immunofluorescence analysis of internalized TRAILR1 (green) in control, SNX1+2-KO, SNX5+6-KO, and SNX27-KO HeLa cells, with endosomes stained with an anti-EEA1 antibody (red). Cells were incubated with antibody against the ectodomain of TRAILR1 and the lysosomal protease inhibitor leupeptin for 6 h. Cells were then fixed, permeabilized, and stained with antibodies against EEA1 and the internalized TRAILR1 antibody. (E) Colocalization of internalized TRAILR1 antibody and EEA1 in cells in D. Each dot represents Pearson’s correlation coefficients from one cell. ****P < 0.0001. Experiments were triplicated, and the numerical data are included in S1 Data. (F) Immunofluorescence analysis of internalized TRAILR1 (green) in control, SNX1+2-KO, SNX5+6-KO, and SNX27-KO HeLa cells, with lysosome stained with an anti-LAMP1 antibody (red). Cells were incubated with antibody against the ectodomain of TRAILR1 and the lysosomal protease inhibitor leupeptin for 6 h. Cells were then fixed, permeabilized, and stained with antibodies against LAMP1 and the internalized TRAILR1 antibody. (G) Colocalization of internalized TRAILR1 antibody and LAMP1 in cells in F. Each dot represents Pearson’s correlation coefficients from one cell. *P < 0.05; ****P < 0.0001. Experiments were triplicated, and the numerical data are included in S1 Data. (H) Steady-state localization of Venus-TRAILR1 WT and mutants in HeLa cells. Cells were transfected with Venus-TRAILR1 constructs (green) and stained with phalloidin (red). Representative images are shown. (I) Colocalization of Venus-TRAILR1 and phalloidin in cells in H. Each dot represents Pearson’s correlation coefficients from one cell. ****P < 0.0001. Experiments were triplicated, and the numerical data are included in S1 Data. BAR, Bin/Amphiphysin/Rvs; CED-1, Cell death abnormality protein 1; CI-MPR, cation-independent mannose 6-phosphate receptor; EEA1, early endosome antigen 1; GO, Gene Ontology; GPCR, G protein–coupled receptor; GST, glutathione-S-transferase; KO, knockout; LAMP1, Lysosomal-associated membrane protein 1; MBP, maltose binding protein; PTHR, Parathyroid hormone/parathyroid hormone-related peptide receptor; SBM, SNX-BAR-binding motif; SNX, Sorting Nexin family; STIMA, STIM Activating Enhancer; TRAILR1, TNF-related apoptosis-inducing ligand receptor 1; WT, wild type.

Having identified and confirmed the SBM within the known SNX-BAR interacting proteins, we next decided to use this consensus motif to identify additional cargoes from the human proteome. We focused our search on transmembrane proteins localized in the plasma membrane, endosome, Golgi, and lysosome, and searched one of the two following patterns: [ILMV]X[FY]X[RK]X_2-13_ΦXΦ (Type I) or [FYW]X[FY]X_3-15_ΦXΦ (Type II) [37, 38]. Since the first three residues of all known SNX-BAR ligands are predicted to be part of a β-strand, and a coil connects the two hydrophobic segments, we further filtered our search results using the secondary structure information around the motif. Finally, we excluded those hits that contain the SBM within a large folded domain. Using the above criteria, we were able to identify 71 putative SNX-BAR ligands and cargo molecules, with three of them (Calcium homeostasis modulator protein 2 [CAHM2], SLIT and NTRK-like protein 2/4 [SLITRK2], Transmembrane protein 209 [TM209]) containing two SBMs (Fig 4A and S7A Fig, S1 Table). These molecules include G protein–coupled receptor (GPCR) and non-GPCR receptors (25 in total), cadherin (16), enzymes (9), ion channels and transporters (8), and proteins of unknown function (Fig 4A). Interestingly, a number of these ligands or their closely related homologs have been identified in previous proteomic studies using SNX27 or VPS35-suppressed cells, including TRAILR1, Parathyroid hormone/parathyroid hormone-related peptide receptor (PTHR), Roundabout Guidance Receptor 1 (ROBO1), Low-density lipoprotein receptor-related protein 6 (LRP6), SLITRK2/4, Transmembrane protein 87A/B (TM87A/B), and CA043 [14].

In order to test our in silico predictions, we chose four proteins from the list and tested their interactions with the SNX1/SNX5 complex. We also included the cytoplasmic tail of *Caenorhabditis elegans* Cell death abnormality protein 1 (CED-1) in our assay. The recycling of CED-1 is critical for apoptotic cell clearance and requires both SNX-BARs and retromer [39]; however, CED-1 does not possess an SBM that we have identified. Three recombinant proteins (PTHR, TRAILR1, and JAM3) that we tested displayed a remarkable binding toward SNX1/SNX5, similar to CI-MPR (Fig 4B). In contrast, STIM Activating Enhancer (STIMA) and CED-1 failed to effectively retain SNX1/SNX5. In order to assess whether the binding was mediated by the predicted SBMs, we made alanine substitutions for critical residues within the SBMs of PTHR and TRAILR1. Alanine substitutions at positions 3 and 5 or positions 21 and 23 completely abolished the interaction between SNX1/SNX5 and PTHR or TRAILR1, confirming the importance of SBMs for the binding (Fig 4C).

PTHR is a member of the secretin family of GPCR and plays a critical role in bone morphogenesis (S7B Fig). It is well established that PTHR is recycled through SNX27 and retromer [40–42]. As we have discovered that PTHR is also a putative ligand of SNX-BARs, we sought to determine how the interactions with SNX-BARs contributed to PTHR endocytic trafficking. KO of SNX5/6 (SNX5+6 KO) or SNX27 (SNX27 KO) had little effect on the endocytosis of PTHR, indicated by the colocalization of CD8A-PTHR and phalloidin in the antibody uptake assay (S7C Fig). In contrast, the endocytosed CD8A-PTHR displayed a decreased colocalization with EEA1 in both SNX5+6 KO and SNX27 KO cells, indicating that SNX5/6 could be involved in endocytic recycling of PTHR, similar to SNX27 (S7D Fig). PTHR contains a putative adaptor protein complex 2 (AP-2) recognition motif in its cytoplasmic tail, which partially overlaps with the SBM that we have identified (S7B Fig). To establish that the SBM of PTHR is the motif critical for its recycling, we introduced a mutation (Y3A) that is outside of the putative AP-2 recognition motif (S7B Fig). The Y3 mutation did not impair the internalization of CD8A-PTHR but displayed a reduced endosomal localization, indicated by diminished colocalization with EEA1 (S7E and S7F Fig). This is consistent with the inability of the Y3 mutant to interact with and be recycled by SNX5/6. Overall, we demonstrate, using a reporter construct, that PTHR is a cargo likely transported by SNX-BARs, but the physiological role of this new pathway remains to be established.

TRAILR1, also known as Death receptor 4 (DR4) or tumor necrosis factor receptor superfamily member 10A (TNFRSF10A), mediates apoptosis through binding to TRAIL [43]. Although it is known that endocytic recycling of TRAILR1 requires SNX27 and retromer, it remains unclear how TRAILR1 is selected for recycling, as it does not possess a PDZbm required to bind the SNX27-retromer complex [14]. To assess the role of SNX-BARs in TRAILR1 trafficking, we compared the levels of TRAILR1 in SNX1/2/5/6- and VPS35-suppressed cells. Immunofluorescence staining of endogenous TRAILR1 revealed a marked reduction at the cell surface in SNX1/2/5/6-suppressed cells, comparable with the VPS35-suppressed cells [14] (S8A–S8C Fig). Furthermore, KO of SNX1/2 using CRISPR-Cas9 vectors simultaneously targeting SNX1 and SNX2 (SNX1+2 KO), or KO of SNX5/6 (SNX5+6 KO), also caused a severe loss of total TRAILR1, similar to the SNX27 KO cells (S8D–S8F Fig). To test whether the loss of TRAILR1 could be due to decreased endosomal recycling and enhanced lysosomal degradation, we performed uptake assays with an antibody against the extracellular of TRAILR1 in the presence of lysosomal proteases (Fig 4D–4G). In control cells, TRAILR1 antibody showed a strong colocalization with EEA1, which was decreased in SNX1+2 KO, SNX5+6 KO, and SNX27 KO cells (Fig 4D and 4E). Conversely, the colocalization between TRAILR1 and LAMP1 was dramatically increased in all three KO cell lines, relative to control cells (Fig 4F and 4G).

To demonstrate the importance of the SBM within TRAILR1 for recycling, we engineered a CD8A-TRAILR1 chimera [20]. Similar to endogenous TRAILR1, CD8A-TRAILR1 entered the degradative pathway in the SNX5+6 or SNX27 KO cells, as indicated by the increased colocalization of CD8A-TRAILR1 and LAMP1 (S9A and S9B Fig). The CD8A-TRAILR1 F3Y5 and V21L23 mutants displayed a strong colocalization with phalloidin, similar to WT, suggesting that mutation of SBM in TRAILR1 does not affect its endocytosis (S9C and S9D Fig). In contrast, both F3Y5 and V21L23 mutants showed a decreased colocalization with EEA1, consistent with defects in protein recycling (S9E and S9F Fig). These observations were further confirmed by our assays with engineered Venus-TRAILR1. Whereas Venus-TRAILR1 WT was predominately localized in the cytosol, both F3Y5 and V21L23 mutants were significantly redistributed to the cell periphery, as assessed by the colocalization with phalloidin (Fig 4H and 4I). Taken together, SNX-BARs likely cooperate with SNX27 and retromer to mediate the recycling of TRAILR1, via direct binding of the SBM in TRAILR1.

**Fig 5:**
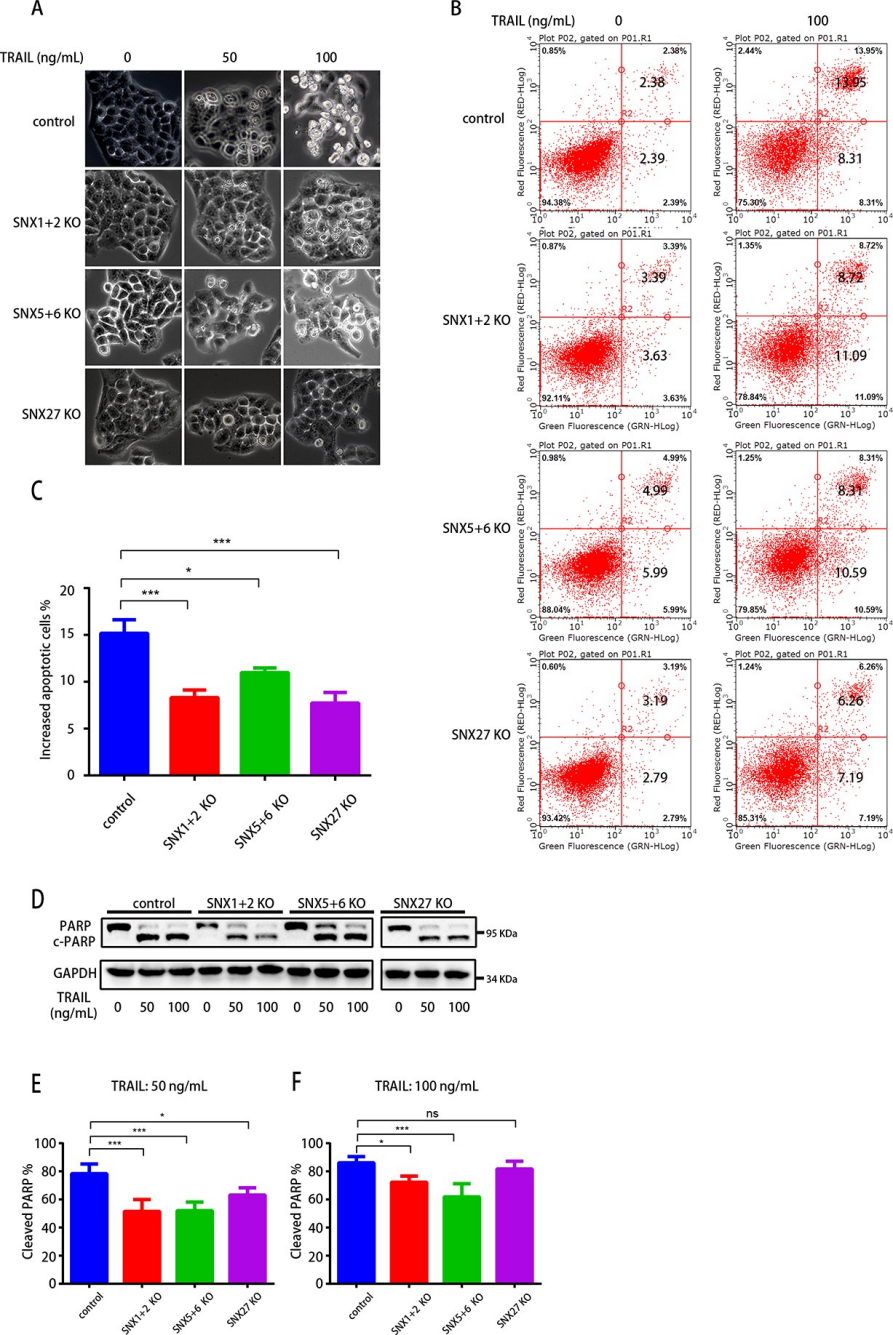
Depletion of SNX-BARs or SNX27 attenuates TRAIL-induced apoptosis. (A) Cell morphology of control, SNX1+2-KO, SNX5+6-KO, and SNX27-KO HeLa cells treated with different concentrations of GST-Trail (0, 50, and 100 ng/mL) for 24 h. (B) Cells were treated with GST-Trail (0 or 100 ng/mL) for 24 h, subjected to Annexin V/PI staining, and analyzed by flow cytometry. (C) Quantification of increased apoptotic cells upon the treatment of GST-TRAIL (apoptotic cell % _with GST-TRAIL_ − apoptotic cell % _without GST-TRAIL_), as shown in B. Statistic data represent the results from *n* = 5 independent experiments and are expressed as mean ± SEM *P < 0.05; ***P < 0.001. The numerical data are included in S1 Data. (D) Protein lysates from control, SNX1+2-KO, SNX5+6-KO, and SNX27-KO KO HeLa cells treated with different concentrations of GST-Trail. Representative blot from *n* = 4 independent experiments. (E-F) Quantification of c-PARP upon the treatment of GST-TRAIL at a concentration of 50 ng/mL (E) or 100 ng/mL (F). Percentage of c-PARP was calculated using the band intensity of c-PARP divided by the total bond intensity of cleaved and uncleaved PARP. Statistic data represent the results from *n* = 4 independent experiments and are expressed as mean ± SD. *P < 0.05; ***P < 0.001. The numerical data are included in S1 Data. BAR, Bin/Amphiphysin/Rvs; c-PARP, cleaved PARP; GAPDH, glyceraldehyde 3-phosphate dehydrogenase; GST, glutathione-S-transferase; KO, knockout; ns, not significant; PARP, Poly(ADP-Ribose) Polymerase; PI, propidium iodide; SNX, Sorting Nexin family; TRAIL, TNF-related apoptosis-inducing ligand.

After demonstrating that SNX-BARs are critical for the endosomal recycling and stability of TRAILR1, we next focused on addressing the functional relevance. As expected, treatment of control cells with recombinant TRAIL protein led to apoptosis of a large portion of cells, with the percentage of apoptotic cells increasing with increasing concentration of TRAIL protein (Fig 5A) [43]. The amount of apoptotic cells was significantly lower in SNX1+2 KO, SNX5+6 KO, and SNX27 KO cells, following TRAIL addition (Fig 5A). This observation was further supported by Annexin V/propidium iodide (PI) staining and fluorescence-activated cell sorting (FACS) analysis, which revealed decreased TRAIL-induced apoptosis in all three KO cells relative to the control cells (7.7%–11% for KO cells versus 15% for control cells) (Fig 5B and 5C). This decrease of apoptosis correlated with our observed reduction of overall TRAILR1 protein level in the KO cells (S8 Fig). Lastly, immunoblot analysis of control and KO cells revealed less Poly(ADP-Ribose) Polymerase (PARP) cleavage in all the KO cells, following the addition of TRAIL at two different concentrations (Fig 5D, 5E and 5F). Altogether, our results indicated that SNX-BAR or SNX27 depletion attenuated TRAIL-induced apoptosis, likely through regulating the trafficking and stability of TRAILR1.

## Discussion

**Fig 6:**
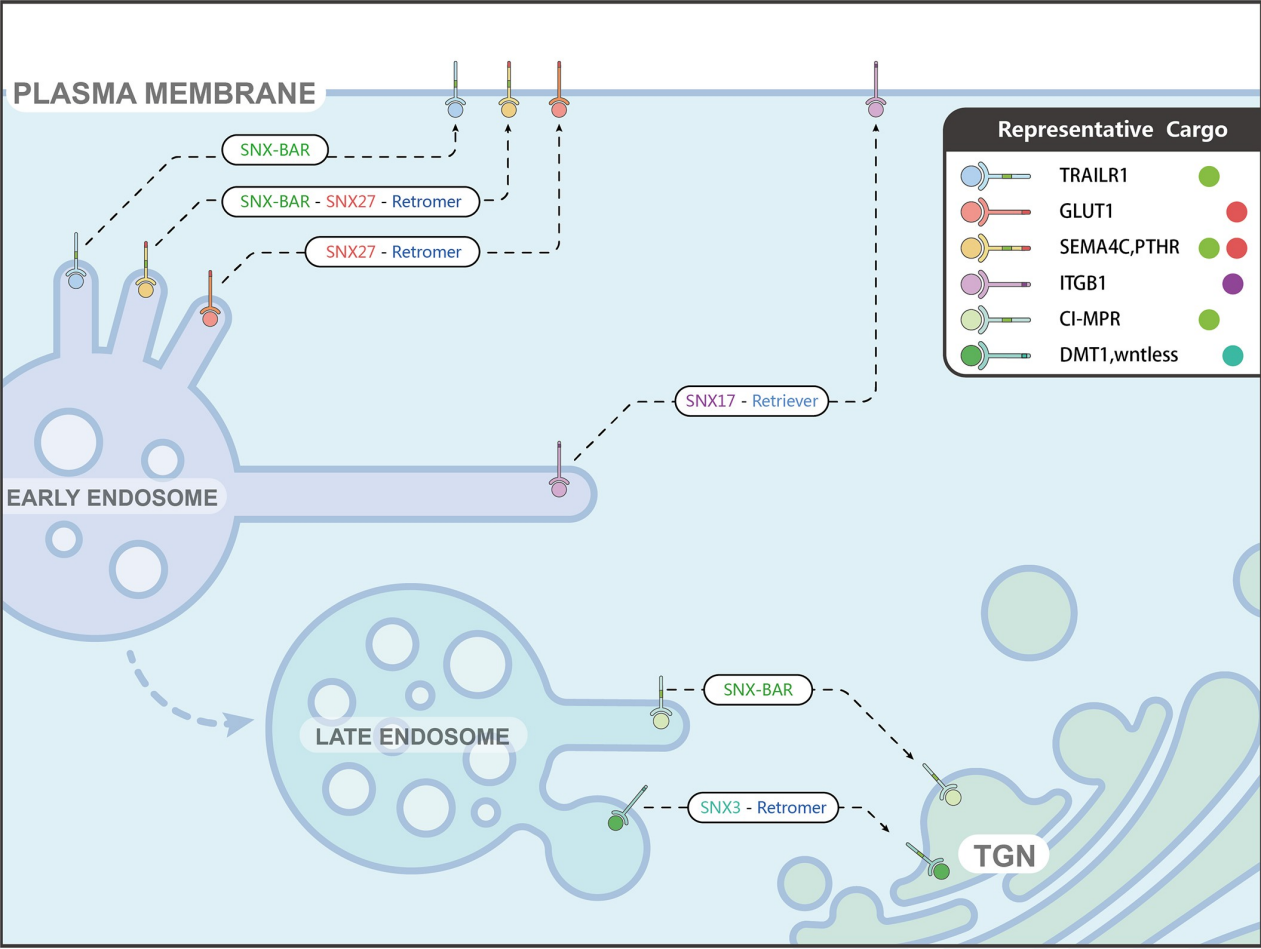
Model showing how sorting nexins mediate cargo transport. SNX-BARs and SNX3-retromer mediate the endosome-to-TGN transport of CI-MPR and DMT1/wntless, respectively. SNX17 and retriever mediate the recycling of transmembrane cargo proteins, such as ITGB1, to the plasma membrane. Finally, both SNX27-retromer and SNX-BARs are involved in the endocytic transport of a variety of cargo, likely through cooperating with each other, although definitive evidence is still lacking. BAR, Bin/Amphiphysin/Rvs; CI-MPR, cation-independent mannose 6-phosphate receptor; DMT1, divalent metal transporter 1; GLUT1, Glucose Transporter Type 1; ITGB1, Integrin Subunit Beta 1; PTHR, Parathyroid hormone/parathyroid hormone-related peptide receptor; SEMA4C, semaphorin 4C; SNX, Sorting Nexin family; TGN, trans-Golgi network; TRAILR1, TNF-related apoptosis-inducing ligand receptor 1.

In this study, we have defined a conserved SBM that is necessary for the binding to the SNX-BAR modules. Defining this SBM not only allows us to explain how the SNX-BARs associate with their known cargoes but also leads to the identification of a large set of putative ligands with broad functions, including TRAILR1 and SEMA4C. The SBM is distinct from the short motifs recognized by the SNX3-retromer [13], SNX17 [44], and SNX27 [14, 45], suggesting that each subfamily of the SNX proteins play a major role in selecting the cargoes, in addition to contacting phosphoinositides and promoting the formation of tubular structures [46] (Fig 6).

We and others demonstrate that SNX-BARs, SNX27, and retromer are all involved in the endocytic recycling of many transmembrane proteins, such as SEMA4C, PTHR, TRAILR1, and Glucose Transporter Type 1 (GLUT1) [14]. Remarkably, TRAILR1 does not possess a PDZbm which is needed for the binding to SNX27, and GLUT1 does not have an SBM. These results, together with an interaction between SNX27 and SNX-BARs [14] and between SNX27 and retromer [14, 47], suggest that mammalian SNX-BARs, SNX27, and retromer form a “supercomplex” to mediate endosome–to–plasma membrane retrieval of certain cargo proteins (Fig 6). Alternatively, SNX-BARs could function in the endosome-to-TGN pathway, as some cargo proteins are recycled to the TGN prior to being delivered to the cell surface. Consistent with our hypothesis, a survey of recently published proteomic data indicates that many proteins (24 out of 66) lost from the surface of SNX-BAR-depleted cells are also affected by SNX27 and VPS35 depletion [14, 48] (S2 Table).

Based on the possession of an SBM and PDZbm, the cargoes can be divided into at least three classes: (1) PDZbm only, represented by GLUT1 and β2 adrenergic receptor (β2AR) [49]; (2) SBM only, represented by IGF1R and TRAILR1; (3) both SBM and PDZbm, such as SEMA4C and PTHR. Interactions between each module of the SNX27-SNX-BAR-retromer supercomplex, such as between SNX1/2 and SNX27 and between SNX27 and retromer, appear to be relatively weak [14, 50] As a result, cargoes able to interact with two different modules simultaneously, such as SEMA4C and PTHR, could greatly facilitate the formation of the supercomplex, which then incorporate the first and second classes of cargoes, or even cargoes that associate with the supercomplex through other means, for recycling.

Although Cui and colleagues reported that retromer and SNX3 can function together to promote the trafficking of CI-MPR [29], we did not detect a direct interaction between the cytoplasmic tail of CI-MPR and the SNX3-retromer complex, suggesting that the SNX-BARs, but not the SNX3-retromer complex, play a dominant role for the retrieval of CI-MPR. The retromer- and SNX3-decorated endosomal transport carriers could incorporate CI-MPR through indirect interactions; alternatively, retromer can modulate the trafficking of CI-MPR by altering lysosomal functions [51].

While our manuscript was under review, Cullen and colleagues have reported the crystal structures of SNX5^PX^ in complex with a peptide encompassing the SBM and also identified dozens of putative SNX-BAR cargoes, using a proteomic approach [48]. Whereas our results are largely consistent with Cullen’s study, including the identification of many identical cargoes, such as ROBO1 and Transmembrane protein 230 (TMEM230), the bioinformatics approach that we used allows identifying cargoes with low abundance or low binding affinity, which could be difficult to detect by proteomic analysis, such as PTHR and TRAILR1. The putative SNX-BAR ligands that we have identified also include proteins involved in cell adhesion, enzymes with a variety of activities, various transporters and ion channels, and many uncharacterized proteins. For most of the SNX-BAR interactome, it remains to be explored how their trafficking and biological functions are modulated by SNX-BARs. For instance, whereas we have identified PTHR as a SNX-BARs-interacting protein and established that SNX-BARs mediates the endocytic recycling of CD8A-PTHR using a reporter construct, its physiological importance remains to be established. Further studies will be necessary to determine whether SNX-BARs play a critical role in bone morphogenesis through recycling of PTHR.

Interestingly, a recent paper by Emr and colleagues revealed that VPS10, the functional homolog of CI-MPR in yeast, also contains a bipartite signal required for its endosomal retrieval [52]. In contrast with CI-MPR, the bipartite signal of VPS10 is recognized by the retromer subunit VPS35 and VPS26, but not by VPS17, the yeast ortholog of SNX5 and SNX6. This difference is partly because VPS17, unlike SNX5 and SNX6, does not encompass within its PX domain the insertion that is required to bind cargoes. The different cargo-recognition mode further emphasizes the significant difference between the yeast retromer and its metazoan counterparts [1]. Altogether, these studies lay a solid foundation for future discoveries concerning the functions of SNX-BARs and retromer in endosomal sorting and signal transduction.

## Methods

### Antibodies and plasmids

DNA constructs and antibodies used in this paper are listed in S3 and S4 Tables, respectively.

### Cell culture and transfection

Cells were maintained in high-glucose Dulbecco’s modified Eagles medium (DMEM) (Hyclone), supplemented with 10% (v/v) fetal bovine serum (FBS) (Biological Industries) and penicillin-streptomycin 1% (v/v) (Hyclone). Cells were grown in an incubator at 37°C with 5% CO_2_ and were transfected using Turbofect Reagent (Thermo Scientific) according the manufacturer’s protocol.

### Immunofluorescence staining and confocal microscopy

Immunofluorescence staining was performed as previously described [30, 31]. Cells were washed with PBS once and then fixed with 4% PFA for 15 min at room temperature (RT). Cells were washed with PBS again and then permeabilized with 0.1% Triton X-100 for 15 min. After being blocked with 2% BSA, the cells were incubated with indicated antibodies overnight at 4°C. Coverslips were washed three times in ice-cold PBS and then incubated with the second antibody. Images were acquired by Zeiss LSM 780 and Olympus FV-1000 confocal microscope and analyzed by NIH ImageJ software. All experiments were repeated at least three times.

### RNAi

siRNA-based knockdown experiments were performed as previously published [23]. Briefly, the siRNA oligonucleotides (Sangon) were transfected using Lipofectamine 3000 (Thermo Fisher Scientific). Twenty-four hours after transfection, the cells were trypsinized and divided into two parts: one was for immunoblotting, and the other was transfected, fixed, and stained, both after an additional 48 h. The targeting sequences were SNX1 (sequence 5-AAGAACAAGACCAAGAGCCAC-3), SNX2 (sequence 5-AAGUCCAUCAUCUCCAGAACC-3′), SNX5 (sequence 5′-CUACGAAGCCCGACUUUGA-3′), SNX6 (sequence 5-UAAAUCAGCAGAUGGAGUA-3′), VPS35-1 (sequence 5′-GUUGUUAUGUGCUUAGUA-3′), VPS35-2 (sequence 5′-AAA UACCAC UUG ACA CUUA-3′).

### Generation of CRISPR KO cell lines

KO cell lines were generated as previously described, with some modifications. Briefly, three gRNAs targeting distinct genomic regions were chosen for each gene, and the corresponding sequences were cloned into the px330 plasmid (gifts from Dr. Florian Steinberg) [22]. Each gRNA-encoding plasmid was mixed with a plasmid encoding GFP-tagged puromycin resistance at a ratio of 1:1 and was transfected into HeLa cells to select the most efficient gRNA. For each targeting gene, one gRNA was chosen (SNX1 gRNA sequence 5′-GAGCCTACAAAGTTACAACAC-3′; SNX2 gRNA sequence 5′-GAAGGGAACCTATCCTATCCT-3′; SNX5 gRNA sequence 5′-GCTCTGAAACGTGGGCAGTG-3′; SNX6 gRNA sequence 5′-GATTATATTCCAAGAAGACA-3′; SNX27 gRNA sequence 5′-GTGCGGGGCCAAGTGAGCGA-3′). Two days after transfection, the medium was replaced with selective medium containing 4 μg/ml puromycin for 48 h. The cells were then recovered in nonselective medium for 3 d, and fresh medium was supplied every day. Five days after transfection, the cells were used for immunoblotting or immunofluorescence staining.

### Surface protein internalization and recycling assays

Internalization and recycling of CD8A-tagged constructs were performed as previously described [20, 21]. Briefly, cytoplasmic tails of transmembrane proteins were fused to the C terminus of CD8A. HeLa cells were transfected with plasmids encoding CD8A-cargo fusion proteins for 24 h before the internalization assay. Monoclonal anti-human CD8A antibody (5 μg/mL in DMEM) was added on ice for 30 min. Unbound antibodies were removed by washing with ice-cold wash buffer (0.1 M glycine and 0.15 M NaCl [pH 3.0]) twice and PBS once. The internalization of antibody-bound CD8A-cargo complexes was carried out in DMEM at 37°C for 1 or 3 h. At indicated time points, cells were fixed with 4% PFA and permeabilized with 0.1% Triton X-100. The internalized CD8A–antibody was detected using Alexa-488 or 546-conjugated secondary antibodies (Jackson).

To assess internalization and recycling of endogenous TRAILR1, HeLa cells were incubated with DMEM containing antibody against the extracellular domain of TRAILR1 (5 μg/ml) and lysosomal protease inhibitor leupeptin (100 μM). After incubation at 37°C for 6 h, unbound antibodies were washed away. Cells were then fixed and permeabilized, and the TRAILR1 antibody was detected by an Alexa-488 coupled secondary antibody (Jackson).

### Surface protein biotinylation assay

Biotinylation assay was performed as previously described with some modifications [53]. Briefly, Cells from three 10-cm dishes were digested with trypsin and washed three times with ice-cold PBS (pH 8.0). The cells were then resuspended into PBS with a final concentration of approximately 1 ×10^7^ cells/mL solution. Biotin reagent (Sulfo-NHS-SS-Biotin, Sangon) was added to the cell suspension with a final concentration of 800 μM and incubated for 30 min at RT. Nonreacting biotin reagent was subsequently removed by washing with ice-cold PBS three times. Next, cells were lysed into a buffer (50 mM Tris [pH 7.5], 50 mM NaCl, 0.5% NP40), and the supernatant was collected. The biotinylated proteins were captured with streptavidin agarose resin (Sangon) for 2 h at RT. The resin was then washed six times with PBS to remove nonspecific binding proteins, and the resin-bound proteins were used for immunoblotting analysis.

### Protein degradation assay

The protocol for in vivo protein degradation assay was adapted from previous reports [22, 23]. Plasmids encoding GLP-VENUS-SEMA4C WT and mutants were transfected into HEK 293T cells using polyethylenimine (PEI). Twenty-four hours after transfection, the cells were divided into five portions (for five different time points) and seeded in a 6-well plate. After an additional 24 h, ribosomal inhibitor cycloheximide was added into the wells, at different times, to a final concentration of 50 μg/ml. Cells were harvested at the same time, lysed, and processed for immunoblotting. Results were quantified over four independent experiments.

### Apoptosis assays

HeLa cells, or SNX1+2-KO, SNX5+6-KO, and SNX27-KO HeLa cells, were treated with different concentrations of recombinant GST-Trail (0, 50, and 100 ng/mL) for 24 h. Cell morphology was observed by OLYMPLUS TH4-200 microscopy. FITC AnnexinV Apoptosis Detection Kit and flow cytometry were used to measure apoptosis. Cells treated with 0 or 100 ng/mL GST-Trail were digested, collected, and centrifuged for 3 min at 3,000*g*. The cells were then washed with cold PBS and centrifuged again for 3 min. Each sample (approximately 1 × 10^5^ cells) was mixed with 200 μL 1×Binding Buffer, 5 μL annexinV-FITC, and 5 μL PI. After incubation for 15 min at RT, samples were diluted with another 300 μL 1×Binding Buffer and subjected to flow cytometry within 15 min.

To measure PARP cleavage triggered by apoptosis, cells were treated as described above and samples were taken for immunoblot analysis. Band intensity was calculated using ImageJ, and results from at least four different experiments were used for statistical analysis.

### Recombinant protein expression and purification

Expression and purification of recombinant proteins was carried out as previously described [28, 30, 54]. GST-tagged SNX5^PX^ was induced by 0.5 mM isopropyl β-D-1-thiogalactopyranoside (IPTG) when cell OD_600_ reached 0.8–1.0, and the cells were grown overnight at 22°C. The protein was first captured by Glutathione Sepharose beads (GE), followed by on-column TEV cleavage. The elution was further purified using Superdex 200 size-exclusion chromatography (GE). The N^15^, C^13^-SNX5^PX^ was expressed using M9 minimal medium and purified with the same protocol. MBP-tagged SNX5 ^PX^, SNX6 ^PX^, and SNX32^PX^ were expressed and purified similarly except that they were subjected to Amylose Resin High Flow (NEB) instead of Glutathione Sepharose beads.

Expression and purification of the retromer complex, SNX1/SNX5, or SNX1ΔN/SNX6 were performed according to the published protocols [28]. SNX3 was expressed with an N-terminal MBP fusion to help its production, and SNX27^PDZ^ was fused to an N-terminal GST tag. These proteins were purified similarly to MBP-tagged SNX5^PX^. GST-tagged CI-MPR, SEMA4C, IGF1R, and other cargoes were all expressed in *Escherichia coli* BL21(DE3) and grown in Luria-Bertani (LB) broth at 37°C.

GST-TRAIL was expressed at 30°C in *E*. *coli* BL21(DE3). The protein was first captured by Glutathione Sepharose beads and then eluted off the beads using a buffer containing 10 mM of GSH. The eluted protein was then subjected to extensive dialysis against the PBS buffer and concentrated to 5 mg/mL for use.

### GST pull-down

GST pull-down experiments were carried out per published reports [27, 31]. Briefly, 20 μg GST or GST-tagged proteins were incubated with Glutathione Sepharose beads in a pull-down buffer (PB: 20 mM Tris-HCl [pH 8.0], 200 mM NaCl, 0.5% Triton-X100) for 1 h, and the unbound protein was then washed away. Bait proteins (100–200 μg) were added to the resin and were incubated for 30 min at 4°C. The beads were then washed three times with 1 ml of PB. Bound proteins were separated by SDS-PAGE and visualized by Coomassie staining or immunoblotting.

For GST pull-down using cell lysate, HEK293T cells were transiently transfected with three vectors (Flag-SNX27, HA-SNX5, GST or GST- SEMA4C). The cells were lysed into the lysis buffer (50 mM Tris-Base [pH 7.5], 50 mM NaCl, 1% NP40) with EDTA-free protease inhibitor cocktail. The cells were then cleared by high-speed centrifugation and incubated with Glutathione Sepharose beads overnight at 4°C. The beads were washed with lysis buffer three times and PBS three times. The bound proteins were detected using anti-GST, anti-SNX1, anti-HA, and anti-FLAG antibodies.

### ITC

ITC experiments were performed on a Microcal iTC200 instrument at 25°C [30, 31]. All proteins and peptides used for ITC experiments were dialyzed overnight at 4°C against an ITC buffer (100 mM HEPES [pH 7.5], 300 mM NaCl, and 2 mM βME). Samples were centrifuged at 12,000 rpm for 10 min before the titration. The titration included one initial injection of 0.2 μl, followed by another 19 injections of 2-μl aliquots, with a spacing of 90 or 120 s between injections. To compare the binding affinity of CI-MPR toward SNX5^PX^, SNX6^PX^, and SNX32^PX^, approximately 2–3 mM of peptides were titrated into approximately 100–300 μM of proteins. For the ITC analysis of CI-MPR and DMT1-II toward SNX5^PX^ and the SNX3-retromer complex, approximately 2–3 mM of peptides were titrated into SNX5^PX^ (300 μM) or the SNX3-retromer complex (10 μM Retromer + 100 μM SNX3). To determine the length of the loop optimal to contact SNX5, approximately 0.5–1 mM of peptides were titrated into SNX5^PX^ (50 μM). All ITC experiments were triplicated. Data were analyzed using the Origin 7.0 software package (OriginLab) by fitting the “one set of sites” model.

### NMR spectroscopy

The NMR experiments were performed as previously described on a Bruker Avance 600M spectrometer equipped with cryoprobe [33]. To obtain backbone resonance assignments, triple-resonance experiments CBCA(CO)NH and CBCANH were recorded at 298 K on a ^15^N/^13^C -labeled SNX^5PX^ sample in 20 mM Tris buffer, 100 mM NaCl, 0.02% NaN_3_ (pH 7.4). To elaborate the binding mode between SNX5PX and CI-MPR, 2D ^1^H, ^15^N-HSQC NMR spectroscopy was performed to monitor the chemical shift perturbation of residues on SNX5^PX^ (100 μM) upon the addition of CI-MPR (aa21–48) (500 μM). All data were processed using NMRPipe software and analyzed with SPARKY.

### Liposome flotation assay

Liposome flotation assays were performed as previously described [31, 55]. All pure synthetic lipids—including 1-palmitoyl-2-oleoyl-sn-glycero-3-phosphocholine (POPC), PtdIns(3)P, and [1,2-dioleoyl- sn-glycero-3-{[N-(5-amino-1-carboxypentyl) iminodiacetic acid] succinyl} (nickel salt) (DOGS-NTA-Ni)—were purchased from Avanti Polar Lipids. Two types of liposomes were generated, POPC/DOGS-NTA-Ni (molar ratio 90:7) and POPC/DOGS-NTA-Ni/PtdIns(3)P (molar ratio 90:7:3). Mixed lipids were extensively dried in a vacuum desiccator for 4 h and then resuspended in a detergent-free buffer (20 mM Hepes-K^+^ [pH 7.5], 150 mM NaCl, and 0.2 mM TCEP). Resuspended mixtures were extruded through a 100-nm polycarbonate filter by a miniextruder (Avanti Polar Lipids) 21 times. Qualities of liposomes were estimated by dynamic light scattering.

Each sample contained 1.5 mM of liposomes (total lipids), 40 μg of SNX1ΔN/6 WT or mutants, and 20 μg of His-CI-MPR WT or mutant, in a total volume of 100 μL. After incubation at 16°C for 4 h, the protein liposome mixtures were subjected to a Histodenz density gradient (40%, 35%, 30%, 0%), which had a total volume of approximately 600 μL. Samples were then centrifuged at 48,000 rpm, 4°C, for 4 h in an SW55Ti rotor (Beckman Coulter). After centrifugation, samples that had floated to the top of the gradient (30 μL) were analyzed by SDS-PAGE.

### Whole-proteome identification of SBM

To search the SBM in human proteome, we used TMHMM (http://www.cbs.dtu.dk/services/TMHMM/) [38] to predict transmembrane proteins and WoLF PSORT (https://wolfpsort.hgc.jp/) [37] to predict the proteins’ subcellular locations. Transmembrane proteins localized in the plasma membrane, endosome, Golgi, and lysosome were chosen to search either of the two following patterns within their cytoplasmic region (for single-pass transmembrane protein) or last cytoplasmic region (for multipass):

- Type I: [ILMV]X[FY]X[RK]X_2-13_ΦXΦ
- Type II: [FYW]X[FY]X_3-15_ΦXΦ

GOR IV (https://npsa-prabi.ibcp.fr/cgi-bin/npsa_automat.pl?page=npsa_gor4.html) [56] or Jpred4 (http://www.compbio.dundee.ac.uk/jpred/index.html) [57] was used to predict the secondary structure around potential motifs to make sure that (1) the first three amino acids in either pattern are localized in a β-strand, at least for one second-structure prediction tool; (2) a random coil existed within the eight amino acids N-terminal to the motif; (3) a random coil existed between two hydrophobic segments. Lastly, the search results were manually inspected using information from UniProt [58], InterPro [59], and HHpred [60]. Only motifs located outside of the folded domains were chosen.

### Classification of SBM-containing proteins

To analyze the molecular functions of SBM-containing proteins, we used the latest version of Gene Ontology (GO) database (released on 4 Dec 2018), which contained 19,716 nonredundant human proteins annotated with at least one GO term, including 71 SBM-containing proteins. Here, we defined the following:

- *N* = number of proteins in human proteome annotated by at least one GO term
- *n* = number of proteins in human proteome annotated by the GO term *t*
- *M* = number of SBM-containing proteins annotated by at least one GO term
- *m* = number of SBM-containing proteins annotated by the GO term *t*

Enrichment ratio (E-ratio) of the GO term *t* was calculated, and the *P* value was calculated with the hypergeometric distribution as below:

$$E-\mathrm{ratio}=\frac{\frac{m}{M}}{\frac{n}{N}}$$

$$p-value=\sum_{m\prime =m}^{n}\frac{\left(\begin{array}{c} M\\ m\prime \end{array})(\begin{array}{c} N-M\\ n-m\prime \end{array}\right)}{\left(\begin{array}{c} N\\ n \end{array}\right)}$$

(E-ratio ≥ 1)

### Statistical analysis

All cellular experiments were performed at least three times. Images were blindly scored for cellular localization, and graphs represent means ± SEM. For the protein degradation assays, results were quantified over four independent experiments, and graphs represent means ± SD. Statistical analyses were performed using one-way ANOVA and Tukey's multiple comparisons test incorporated in Prism 7 (GraphPad Software) (***P < 0.001, **P < 0.01, *P < 0.05, ns: not significant).

## Supporting information

S1 Fig CI-MPR preferentially interacts with SNX-BARs over the SNX3-retromer and binds to the PX domain of SNX5/6/32. (A) GST-CI-MPR (aa21–48) or GST pull-down of purified VPS35/VPS26/SNX3, and MBP-SNX1/His6-sumo-SNX5. Shown is a Coomassie blue–stained SDS-PAGE gel of purified proteins used (left) and bound samples (right). (B) Isothermal titration calorimetry of CI-MPR (aa21–48) titrated into SNX5PX, SNX6PX, or SNX32PX in a buffer containing 100 mM Hepes (pH 7.5), 300 mM NaCl, 2 mM βME at 25°C. Top and bottom panels show raw and integrated heat from injections, respectively. The black curve in the bottom panel represents a fit of the integrated data to a single-site binding model. Experiments were triplicated, and the numerical data are included in S1 Data. aa, amino acid; BAR, Bin/Amphiphysin/Rvs; CI-MPR, cation-independent mannose 6-phosphate receptor; GST, glutathione-S-transferase; MBP, maltose binding protein; PX, phox-homology; SNX, Sorting Nexin family; VPS, vacuolar protein sorting.
(TIF)

S2 Fig Comparative sequence alignment of PX domains from 29 human SNX proteins. The alignment was conducted using ClustalW, with secondary structure of SNX5 listed above. Residues predicted to be critical for CI-MPR binding were highlighted in machaccino. CI-MPR, cation-independent mannose 6-phosphate receptor; PX, phox-homology; SNX, Sorting Nexin family.
(TIF)

S3 Fig Identification of SNX5PX residues critical for contacting CI-MPR. (A) Overlays of the 2D ^1^H-^15^N HSQC spectra of ^15^N-^13^C-labeled SNX5PX in its free form (green, 100 μM) and in the presence of 5 molar equivalents of unlabeled CI-MPR peptide (aa21–48) (black). NMR spectra were recorded on a ^13^C/^15^N-labeled sample in 20 mM Tris buffer (pH 7.4), 100 mM NaCl, 0.02% NaN3. (B) GST-CI-MPR pull-down of purified MBP-SNX5PX WT or mutants (E129A, Y132D, L133A, F136D, E144A). Shown is a Coomassie blue–stained SDS-PAGE gel of bound samples. (C) GST-CI-MPR pull-down of purified MBP-SNX5PX in the presence or absence of IncE. Shown is a Coomassie blue–stained SDS-PAGE gel of purified proteins used (left) and bound samples (right). The molar ratio of GST-CI-MPR and competing protein IncE is indicated at the top of the gel. aa, amino acid; CI-MPR, cation-independent mannose 6-phosphate receptor; GST, glutathione-S-transferase; MBP, maltose binding protein; NMR, nuclear magnetic resonance; PX, phox-homology; SNX, Sorting Nexin family; SNX5PX, PX domain of SNX5; WT, wild type.
(TIF)

S4 Fig Identification of CI-MPR and IGF1R residues critical for contacting SNX5. (A) Shown is a Coomassie blue–stained SDS-PAGE gel of bound proteins by immobilized GST-CI-MPR. Results are representative of three independent experiments. Amount of MBP-SNX5^PX^ retained was expressed relative to the amount of GST-CI-MPR in the bound sample and then normalized to the amount of WT protein. The numbers below the SDS-PAGE indicate the relative binding. (B) Shown is a Coomassie blue–stained SDS-PAGE gel of bound proteins by immobilized GST-CI-MPR WT or mutants deleting the loop. Results are representative of three independent experiments. (C) Isothermal titration calorimetry of CI-MPR (aa21–48) WT or mutants deleting the loop titrated into SNX5^PX^ in a buffer containing 100 mM Hepes (pH 7.5), 300 mM NaCl, 2 mM βME at 25°C. Top and bottom panels show raw and integrated heat from injections, respectively. The black curve in the bottom panel represents a fit of the integrated data to a single-site binding model. Experiments were triplicated, and the numerical data are included in S1 Data. (D) GST-IGF1R tail WT or mutants (F3Y5, Y5H), or GST-INS1R tail WT or H5Y mutant, or GST pull-down of purified MBP-SNX5^PX^. Shown are a Coomassie blue–stained SDS-PAGE gel of purified proteins (bottom) and immunoblot using anti-MBP antibody for the same sample (top). The GST-IGF1R and GST-INS1R samples contained multiple degraded proteins. aa, amino acid; CI-MPR, cation-independent mannose 6-phosphate receptor; GST, glutathione-S-transferase; IGF1R, Insulin-like growth factor 1 receptor; INS1R, insulin receptor 1; MBP, maltose binding protein; SNX, Sorting Nexin family; WT, wild type.
(TIF)

S5 Fig SEMA4C is recognized by both SNX-BARs and SNX27. (A) SEMA4C interacts with SNX1, SNX5, and SNX27 in cells. HEK293T cells were transiently transfected with vectors encoding Flag-SNX27 and HA-SNX5 together with those encoding GST, GST-SEMA4C-tail (aa1149), or GST-SEMA4C-Δ4 (aa1-145). The cells were lysed, and the supernatant was subjected to Glutathione Sepharose beads. The bound proteins were detected using anti-GST, anti-SNX1, anti-HA, and anti-FLAG antibodies. (B) GST, GST-CI-MPR, GST-SEMA4C-tail (aa1–149), or GST-SEMA4C (aa47–71) pull-down of purified MBP-SNX5^PX^. Shown is a Coomassie blue–stained SDS-PAGE gel of purified proteins used (left) and bound samples (right). (C) GST, GST-SEMA4C-(aa1–149)-Y3Y5, or GST-SEMA4C-Δ4-Y3Y5 pull-down of purified MBP-SNX5^PX^, or SNX27^PDZ^, or the mixture of MBP-SNX5^PX^ and SNX27^PDZ^. Shown is a Coomassie blue–stained SDS-PAGE gel of purified proteins used (left) and bound samples (right). (D) Recombinant GST-SEMA4C WT or mutants pull-down of SNX2/SNX6 from cells. HEK293T cells were transiently transfected with HA-YFP-SNX2 and HA-YFP-SNX6. The cells were lysed 36 h after transfection, and the bound proteins were detected by anti-GFP antibody. Shown is a Coomassie blue–stained SDS-PAGE gel of input GST or GST-SEMA4C proteins (bottom) and immunoblot for the bound samples (top). aa, amino acid; BAR, Bin/Amphiphysin/Rvs; CI-MPR, cation-independent mannose 6-phosphate receptor; GFP, green fluorescent protein; GST, glutathione-S-transferase; HA, hemagglutinin; MBP, maltose binding protein; SEMA4C, semaphorin 4C; SNX, Sorting Nexin family; WT, wild type; YFP, yellow fluorescent protein.
(TIF)

S6 Fig Both SNX-BARs and SNX27 are required for endocytic recycling of SEMA4C. (A) Specific loss of SEMA4C, CI-MPR and TRAILR1 in SNX5/6 KO and SNX27 KO cells. HeLa cells were transfected with CRISPR-Cas9 plasmids targeting SNX27, simultaneous SNX5 and SNX6 (SNX5+6), or empty vector (control). Endogenous protein levels in the polyclonal population were analyzed by immunoblotting, and surface fractions were analyzed for the abundance of the indicated proteins. Integrin 5 is a cargo of neither SNX27 nor SNX-BAR and is used as a control. (B) Immunoblots from A were quantified using ImageJ (*n* = 3) and normalized to the levels of control cells. Error bars represent standard deviation. *P* values were compared with WT using one-way ANOVA, Tukey's multiple comparisons test. *P < 0.05; ***P < 0.001; ****P < 0.0001. (C) SBM and/or PDZbm are not required for the internalization of CD8A-SEMA4C. HeLa cells were transiently transfected with plasmids encoding CD8A-SEMA4C WT, L21I23, 4, and 4-L21I23. Cells were incubated with monoclonal anti-human CD8A antibody on ice for 30 min. Unbound antibodies were removed. The internalized CD8A–antibody was detected using Alexa-488 secondary antibodies, with plasma membrane stained with phalloidin (red). Scale bar: 10 μm. (D) Quantification of CD8A/phalloidin colocalization in cells in C. Each dot represents Pearson’s correlation coefficients from one cell. Experiments were triplicated, and the numerical data are included in S1 Data. *P* values were calculated using one-way ANOVA and Tukey's multiple comparisons test. (E-F) SEMA4C degradation assays. HeLa cells were transfected with WT or mutant Venus–SEMA4C constructs. Forty-eight hours after transfection, cells were treated with the ribosomal inhibitor cycloheximide (50 g/ml) for the indicated time periods (F). The levels of Venus-SEMA4C were analyzed by immunoblotting using an anti-GFP antibody. Amount of Venus-SEMA4C was expressed relative to the amount of GAPDH (loading control) and then normalized to the sample at 0 h. Graph shows the degradation kinetics, with error bars indicating SD (G). *P < 0.05 comparison of WT and 4-L21I23 in a one-way ANOVA test. Experiments were repeated four times, and the numerical data are included in S1 Data. BAR, Bin/Amphiphysin/Rvs; CI-MPR, cation-independent mannose 6-phosphate receptor; GAPDH, glyceraldehyde 3-phosphate dehydrogenase; GFP, green fluorescent protein; KO, knockout; ns, not significant; PDZbm, PDZ-binding motif; SBM, SNX-BAR-binding motif; SEMA4C, semaphorin 4C; SNX, Sorting Nexin family; WT, wild type.
(TIF)

S7 Fig Both SNX-BARs and SNX27 mediates endocytic recycling of PTHR. (A) Seventy-one human SBM-containing proteins were analyzed by GO and classified by their molecular functions. The numerical data are included in S1 Data. (B) Amino acid sequences of the cytoplasmic tail of PTHR, and residues in SBM and PDZbm are colored in green and red, respectively. The putative AP-2 binding motif (YGPM) is indicated with a box. (C-D) Control, SNX5+6 KO, and SNX27 KO HeLa cells were transiently transfected with plasmid encoding CD8A-PTHR. Cells were incubated with monoclonal anti-human CD8A antibody on ice for 30 min. Unbound antibodies were then removed by washing with the low-PH buffer, and cells were then chased in serum-free medium at 37°C for 0 h (C) or 1 h (D). The internalized CD8A–antibody was detected with phalloidin (C) or EEA1 (D). The bottom panel shows quantification with CD8A/phalloidin or CD8A/EEA1. Each dot represents Pearson’s correlation coefficients from one cell. Experiments were triplicated, and the numerical data are included in S1 Data. *P* values were calculated using one-way ANOVA, Tukey's multiple comparisons test. ***P < 0.001. Scale bar: 10 μm. (E-F) HeLa cells were transiently transfected with plasmid encoding CD8A-PTHR WT or Y3 (Y3A). Cells were incubated with monoclonal anti-human CD8A antibody on ice for 30 min. Unbound antibodies were then removed by washing with the low-PH buffer, and cells were then chased in serum-free medium at 37°C for 0 h (C) or 1 h (D). The internalized CD8A–antibody was detected with phalloidin (E) or EEA1 (F). The bottom panel shows quantification with CD8A/phalloidin or CD8A/EEA1. Each dot represents Pearson’s correlation coefficients from one cell. Experiments were triplicated, and the numerical data are included in S1 Data. *P* values were calculated using one-way ANOVA, Tukey's multiple comparisons test. **P < 0.01. Scale bar: 10 μm. AP-2, adaptor protein complex 2; BAR, Bin/Amphiphysin/Rvs; EEA1, early endosome antigen 1; GO, Gene Ontology; KO, knockout; ns, not significant; PDZbm, PDZ-binding motif; PTHR, Parathyroid hormone/parathyroid hormone-related peptide receptor; SBM, SNX-BAR-binding motif; SNX, Sorting Nexin family; WT, wild type.
(TIF)

S8 Fig Suppression of SNX-BARs, SNX27, or VPS35 leads to mislocalization and degradation of TRAILR1. (A) HeLa cells were transiently transfected with control siRNA (“siNC”), or siVPS35, or a combination of siRNAs against SNX1, SNX2, SNX5, and SNX6 (“siSNX1256”). Endogenous protein levels were analyzed by immunoblotting 72 h after the transfection. (B) Immunofluorescence analysis of endogenous TRAILR1 (green) in SNX1256- and VPS35-depleted HeLa cells, with plasma membrane stained with phalloidin (red). (C) Quantification of relative fluorescence intensity of TRAILR1 in B. Experiments were triplicated, and the numerical data are included in S1 Data. Each dot represents average fluorescence intensity from one cell. Scale bar: 10 μm. **P < 0.01; ***P < 0.001. (D) HeLa cells were transfected with CRISPR-Cas9 plasmids targeting SNX27, simultaneous SNX1 and SNX2 (SNX1+2), SNX5 and SNX6 (SNX5+6), or empty vector (control). Endogenous protein levels in the polyclonal population were analyzed by immunoblotting. (E) Immunofluorescence analysis of endogenous TRAILR1 in control, SNX1+2-, SNX5+6-, and SNX27-KO HeLa cells. Scale bar: 10 μm. (F) Quantification of relative fluorescence intensity of TRAILR1 in E. Experiments were triplicated, and the numerical data are included in S1 Data. Each dot represents average fluorescence intensity from one cell. **P < 0.01. BAR, Bin/Amphiphysin/Rvs; KO, knockout; siRNA, small interfering RNA; SNX, Sorting Nexin family; TRAILR1, TNF-related apoptosis-inducing ligand receptor 1; VPS, vacuolar protein sorting.
(TIF)

S9 Fig Mutations of SBM impair endosomal recycling of TRAILR1 without interfering with its internalization. (A) Control, SNX5+6-KO, and SNX27-KO HeLa cells were transiently transfected with plasmids encoding CD8A-TRAILR1. Cells were incubated with monoclonal anti-human CD8A antibody on ice for 30 min. Unbound antibodies were removed, and the internalization of antibody-bound CD8A was carried out in DMEM at 37°C for 3 h. The internalized CD8A–antibody was detected using Alexa-488 secondary antibodies, with lysosomes stained with LAMP1 (red). Scale bar: 10 μm. (B) Quantification of CD8A/LAMP1 colocalization in cells in A. Each dot represents Pearson’s correlation coefficients from one cell. Experiments were triplicated, and the numerical data are included in S1 Data. *P* values were calculated using one-way ANOVA, Tukey's multiple comparisons test. Scale bar: 10 μm. **P < 0.01. (C) HeLa cells were transiently transfected with plasmids encoding CD8A-TRAILR1 WT, F3Y5, and V21L23. Cells were incubated with monoclonal anti-human CD8A antibody on ice for 30 min. Unbound antibodies were removed. The internalized CD8A–antibody was detected using Alexa-488 secondary antibodies, with plasma membrane stained with phalloidin (red). (D) Quantification of CD8A/phalloidin colocalization in cells in C. Each dot represents Pearson’s correlation coefficients from one cell. Experiments were triplicated, and the numerical data are included in S1 Data. *P* values were calculated using one-way ANOVA, Tukey's multiple comparisons test. (E) HeLa cells were transiently transfected with plasmids encoding CD8A-TRAILR1 WT, F3Y5, and V21L23. Cells were incubated with monoclonal anti-human CD8A antibody on ice for 30 min. Unbound antibodies were removed, and the internalization of antibody-bound CD8A was carried out in DMEM at 37°C for 1 h. The internalized CD8A–antibody was detected using Alexa-546 secondary antibodies, with endosomes stained with anti-EEA1 antibody (green). (F) Quantification of CD8A/EEA1 colocalization in cells in E. Each dot represents Pearson’s correlation coefficients from one cell. ***P < 0.001; ****P < 0.0001. Experiments were triplicated, and the numerical data are included in S1 Data. DMEM, Dulbecco’s modified Eagles medium; EEA1, early endosome antigen 1; KO, knockout; LAMP1, Lysosomal-associated membrane protein 1; ns, not significant; SBM, SNX-BAR-binding motif; SNX, Sorting Nexin family; TRAILR1, TNF-related apoptosis-inducing ligand receptor 1; WT, wild type.
(TIF)

S1 Table Seventy-one putative SNX-BAR ligands and cargo molecules determined in this study. BAR, Bin/Amphiphysin/Rvs; SNX, Sorting Nexin family.
(XLSX)

S2 Table Shared cargoes affected by loss of SNX-BARs, SNX27, or Retromer. (a) A list of surface proteins affected by the loss of SNX-BARs was reported by Simonetti and colleagues [48]. (b) A list of surface proteins affected by the loss of SNX27 or enriched in the SNX27 interactome was reported by Steinberg and colleagues [14]. (c). A list of surface proteins affected by the loss of VPS35 was reported by Steinberg and colleagues [14]. (d) Cargo proteins with sequence identity of 40% or higher were listed. BAR, Bin/Amphiphysin/Rvs; SNX, Sorting Nexin family; VPS, vacuolar protein sorting.
(DOCX)

S3 Table DNA constructs used in this study. (DOCX)

S4 Table Summary of antibodies used in this study. (DOCX)

S1 Raw images Unprocessed images of all gels and blots in the paper. (PDF)

S1 Data Numerical data for Figs 1D, 2B, 2D, 2E, 2F, 3E, 3G, 4E, 4G, 4I, 5C, 5E, 5F, S1B, S4C, S6D, S6F, S7A, S7C, S7D, S7E, S7F, S8C, S8F, S9B, S9D and S9F. (XLSX)

## Abbreviations

β2AR: β2 adrenergic receptor
AP-2: adaptor protein complex 2
BAR: Bin/Amphiphysin/Rvs
CAHM2: Calcium homeostasis modulator protein 2
CBB: Coomassie brilliant blue
CED-1: Cell death abnormality protein 1
CI-MPR: cation-independent mannose 6-phosphate receptor
c-PARP: cleaved PARP
DMT1-II: divalent metal transporter 1
DR4: Death receptor 4
EEA1: early endosome antigen 1
FACS: fluorescence-activated cell sorting
GAPDH: glyceraldehyde 3-phosphate dehydrogenase
GLUT1: Glucose Transporter Type 1
GPCR: G protein–coupled receptor
GST: glutathione-S-transferase
IGF1R: Insulin-like growth factor 1 receptor
INSR: insulin receptor
ITC: isothermal titration calorimetry
ITGB1: Integrin Subunit Beta 1
K_d_: dissociation constant
KO: knockout
LAMP1: Lysosomal-associated membrane protein 1
LRP6: Low-density lipoprotein receptor-related protein 6
MW: molecular weight
NMR: nuclear magnetic resonancens, not significant
PARP: Poly(ADP-Ribose) Polymerase
PDZbm: PDZ-binding motif
PI: propidium iodide
PtdIns(3)P: phosphatidylinositol 3-phosphate
PTHR: Parathyroid hormone/parathyroid hormone-related peptide receptor
PX: phox-homology
ROBO1: Roundabout Guidance Receptor 1
SBM: SNX-BAR-binding motif
SEMA4C: semaphorin 4C
SLITRK2/4: SLIT and NTRK-like protein 2/4
SNX: Sorting Nexin family
SNX5^PX^: PX domain of SNX5
STIMA: STIM Activating Enhancer
TGN: trans-Golgi network
TM87A/B: Transmembrane protein 87A/B
TM209: Transmembrane protein 209
TMEM230: Transmembrane protein 230
TNFRSF10A: tumor necrosis factor receptor superfamily member 10A
TRAIL: TNF-related apoptosis-inducing ligand
TRAILR1: TRAIL receptor 1
VPS: vacuolar protein sorting
WT: wild type
