# Supplementary figures and images for: Mechanism of cargo recognition by retromer-linked SNX-BAR proteins
Source: PLoS Biol. 2020 Mar 9;18(3):e3000631. doi: 10.1371/journal.pbio.3000631 (PMC7082075; doi:10.1371/journal.pbio.3000631)

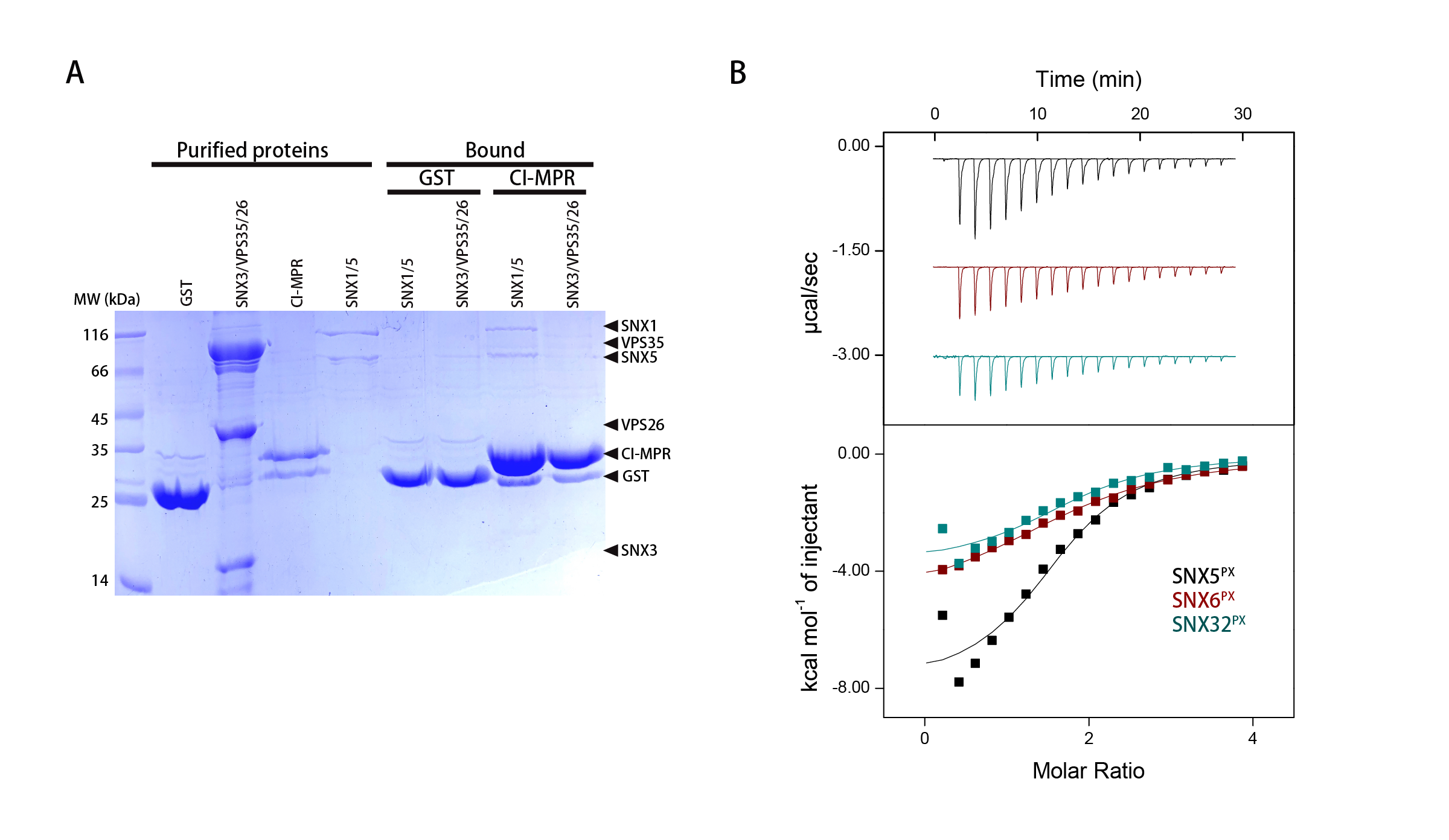

Supplement: S1 Fig — (A) GST-CI-MPR (aa21–48) or GST pull-down of purified VPS35/VPS26/SNX3, and MBP-SNX1/His6-sumo-SNX5. Shown is a Coomassie blue–stained SDS-PAGE gel of purified proteins used (left) and bound samples (right). (B) Isothermal titration calorimetry of CI-MPR (aa21–48) titrated into SNX5PX, SNX6PX, or SNX32PX in a buffer containing 100 mM Hepes (pH 7.5), 300 mM NaCl, 2 mM βME at 25°C. Top and bottom panels show raw and integrated heat from injections, respectively. The black curve in the bottom panel represents a fit of the integrated data to a single-site binding model. Experiments were triplicated, and the numerical data are included in S1 Data. aa, amino acid; BAR, Bin/Amphiphysin/Rvs; CI-MPR, cation-independent mannose 6-phosphate receptor; GST, glutathione-S-transferase; MBP, maltose binding protein; PX, phox-homology; SNX, Sorting Nexin family; VPS, vacuolar protein sorting. (TIF) [file pbio.3000631.s001.tif]

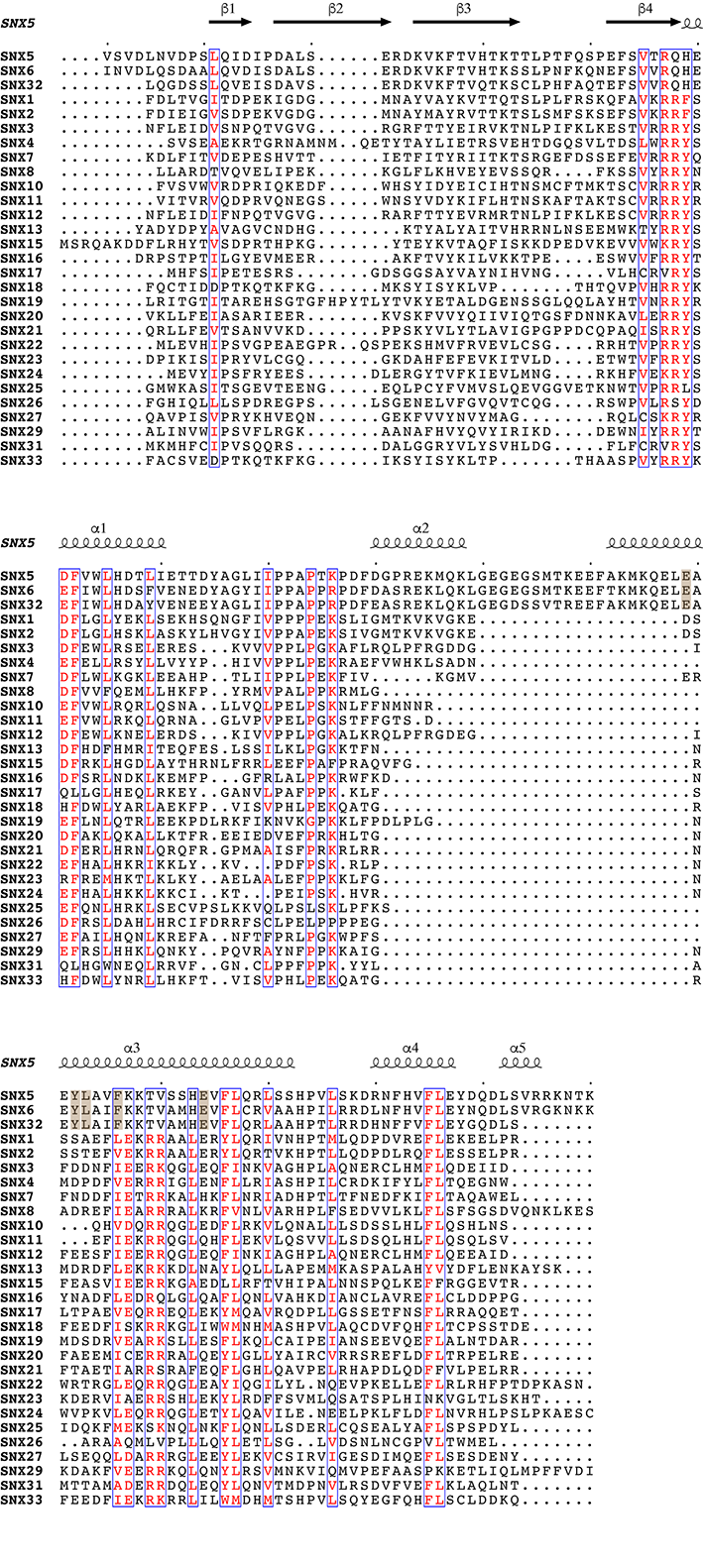

Supplement: S2 Fig — The alignment was conducted using ClustalW, with secondary structure of SNX5 listed above. Residues predicted to be critical for CI-MPR binding were highlighted in machaccino. CI-MPR, cation-independent mannose 6-phosphate receptor; PX, phox-homology; SNX, Sorting Nexin family. (TIF) [file pbio.3000631.s002.tif]

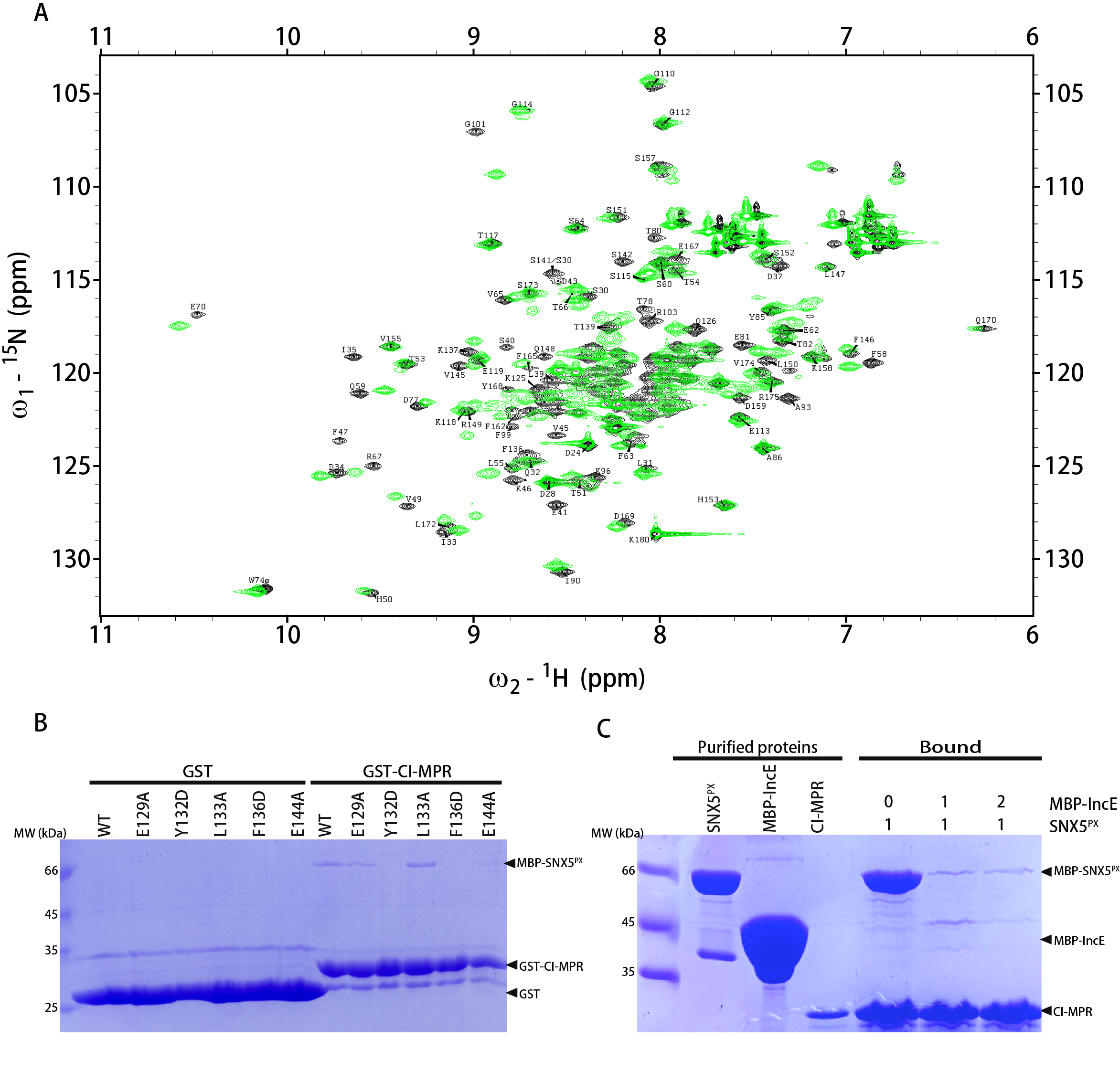

Supplement: S3 Fig — (A) Overlays of the 2D 1H-15N HSQC spectra of 15N-13C-labeled SNX5PX in its free form (green, 100 μM) and in the presence of 5 molar equivalents of unlabeled CI-MPR peptide (aa21–48) (black). NMR spectra were recorded on a 13C/15N-labeled sample in 20 mM Tris buffer (pH 7.4), 100 mM NaCl, 0.02% NaN3. (B) GST-CI-MPR pull-down of purified MBP-SNX5PX WT or mutants (E129A, Y132D, L133A, F136D, E144A). Shown is a Coomassie blue–stained SDS-PAGE gel of bound samples. (C) GST-CI-MPR pull-down of purified MBP-SNX5PX in the presence or absence of IncE. Shown is a Coomassie blue–stained SDS-PAGE gel of purified proteins used (left) and bound samples (right). The molar ratio of GST-CI-MPR and competing protein IncE is indicated at the top of the gel. aa, amino acid; CI-MPR, cation-independent mannose 6-phosphate receptor; GST, glutathione-S-transferase; MBP, maltose binding protein; NMR, nuclear magnetic resonance; PX, phox-homology; SNX, Sorting Nexin family; SNX5PX, PX domain of SNX5; WT, wild type. (TIF) [file pbio.3000631.s003.tif]

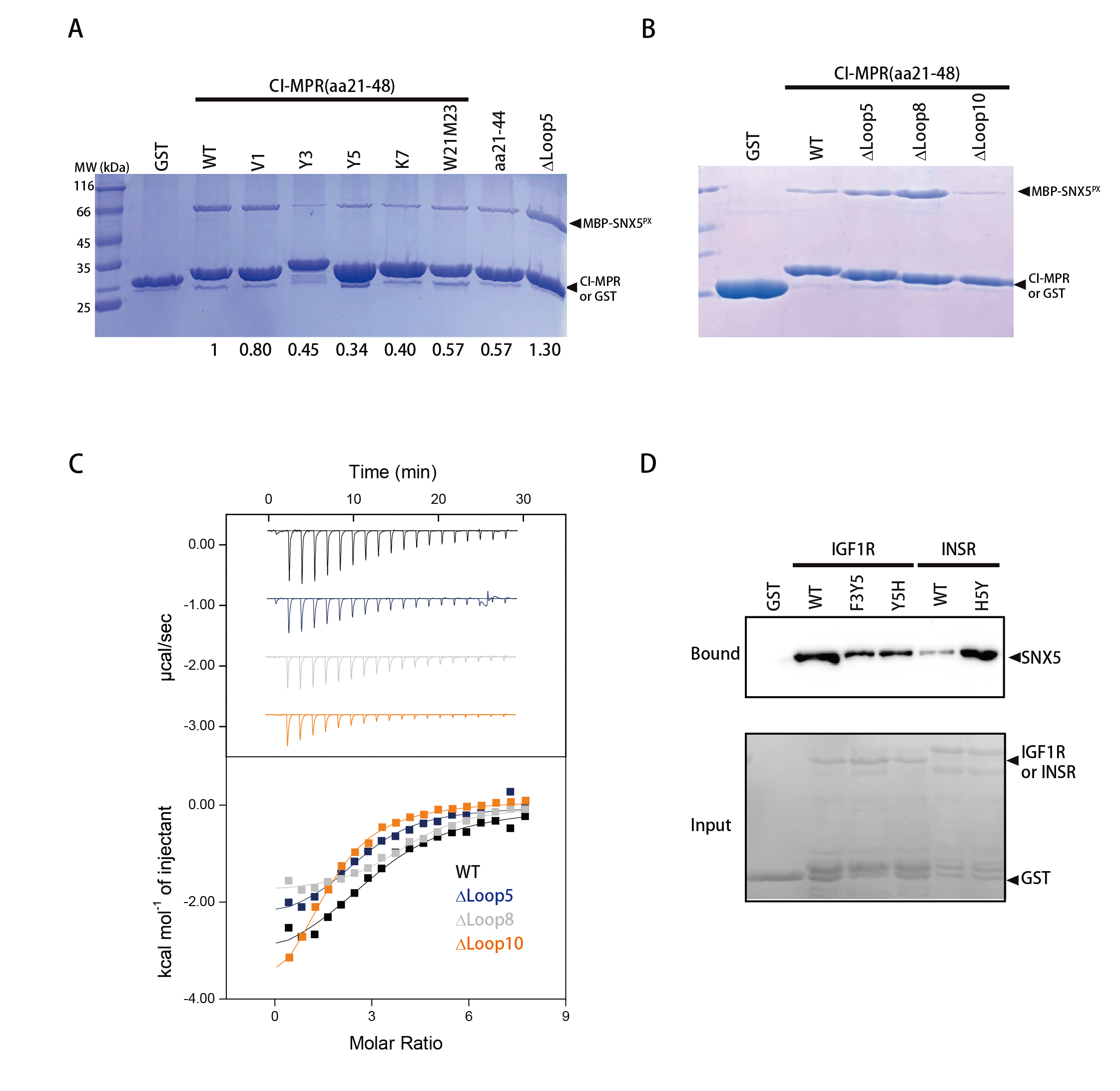

Supplement: S4 Fig — (A) Shown is a Coomassie blue–stained SDS-PAGE gel of bound proteins by immobilized GST-CI-MPR. Results are representative of three independent experiments. Amount of MBP-SNX5PX retained was expressed relative to the amount of GST-CI-MPR in the bound sample and then normalized to the amount of WT protein. The numbers below the SDS-PAGE indicate the relative binding. (B) Shown is a Coomassie blue–stained SDS-PAGE gel of bound proteins by immobilized GST-CI-MPR WT or mutants deleting the loop. Results are representative of three independent experiments. (C) Isothermal titration calorimetry of CI-MPR (aa21–48) WT or mutants deleting the loop titrated into SNX5PX in a buffer containing 100 mM Hepes (pH 7.5), 300 mM NaCl, 2 mM βME at 25°C. Top and bottom panels show raw and integrated heat from injections, respectively. The black curve in the bottom panel represents a fit of the integrated data to a single-site binding model. Experiments were triplicated, and the numerical data are included in S1 Data. (D) GST-IGF1R tail WT or mutants (F3Y5, Y5H), or GST-INS1R tail WT or H5Y mutant, or GST pull-down of purified MBP-SNX5PX. Shown are a Coomassie blue–stained SDS-PAGE gel of purified proteins (bottom) and immunoblot using anti-MBP antibody for the same sample (top). The GST-IGF1R and GST-INS1R samples contained multiple degraded proteins. aa, amino acid; CI-MPR, cation-independent mannose 6-phosphate receptor; GST, glutathione-S-transferase; IGF1R, Insulin-like growth factor 1 receptor; INS1R, insulin receptor 1; MBP, maltose binding protein; SNX, Sorting Nexin family; WT, wild type. (TIF) [file pbio.3000631.s004.tif]

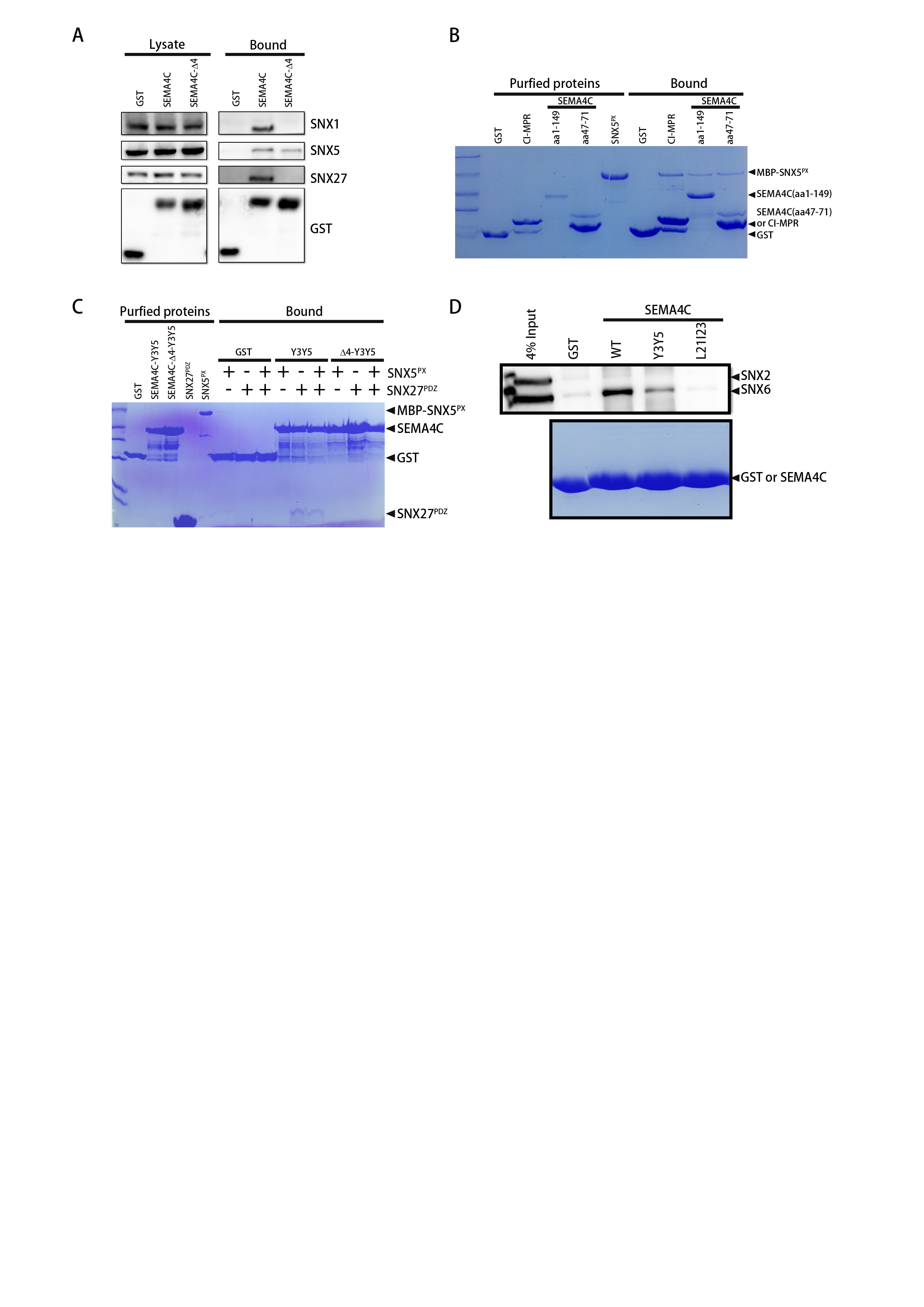

Supplement: S5 Fig — (A) SEMA4C interacts with SNX1, SNX5, and SNX27 in cells. HEK293T cells were transiently transfected with vectors encoding Flag-SNX27 and HA-SNX5 together with those encoding GST, GST-SEMA4C-tail (aa1149), or GST-SEMA4C-Δ4 (aa1-145). The cells were lysed, and the supernatant was subjected to Glutathione Sepharose beads. The bound proteins were detected using anti-GST, anti-SNX1, anti-HA, and anti-FLAG antibodies. (B) GST, GST-CI-MPR, GST-SEMA4C-tail (aa1–149), or GST-SEMA4C (aa47–71) pull-down of purified MBP-SNX5PX. Shown is a Coomassie blue–stained SDS-PAGE gel of purified proteins used (left) and bound samples (right). (C) GST, GST-SEMA4C-(aa1–149)-Y3Y5, or GST-SEMA4C-Δ4-Y3Y5 pull-down of purified MBP-SNX5PX, or SNX27PDZ, or the mixture of MBP-SNX5PX and SNX27PDZ. Shown is a Coomassie blue–stained SDS-PAGE gel of purified proteins used (left) and bound samples (right). (D) Recombinant GST-SEMA4C WT or mutants pull-down of SNX2/SNX6 from cells. HEK293T cells were transiently transfected with HA-YFP-SNX2 and HA-YFP-SNX6. The cells were lysed 36 h after transfection, and the bound proteins were detected by anti-GFP antibody. Shown is a Coomassie blue–stained SDS-PAGE gel of input GST or GST-SEMA4C proteins (bottom) and immunoblot for the bound samples (top). aa, amino acid; BAR, Bin/Amphiphysin/Rvs; CI-MPR, cation-independent mannose 6-phosphate receptor; GFP, green fluorescent protein; GST, glutathione-S-transferase; HA, hemagglutinin; MBP, maltose binding protein; SEMA4C, semaphorin 4C; SNX, Sorting Nexin family; WT, wild type; YFP, yellow fluorescent protein. (TIF) [file pbio.3000631.s005.tif]

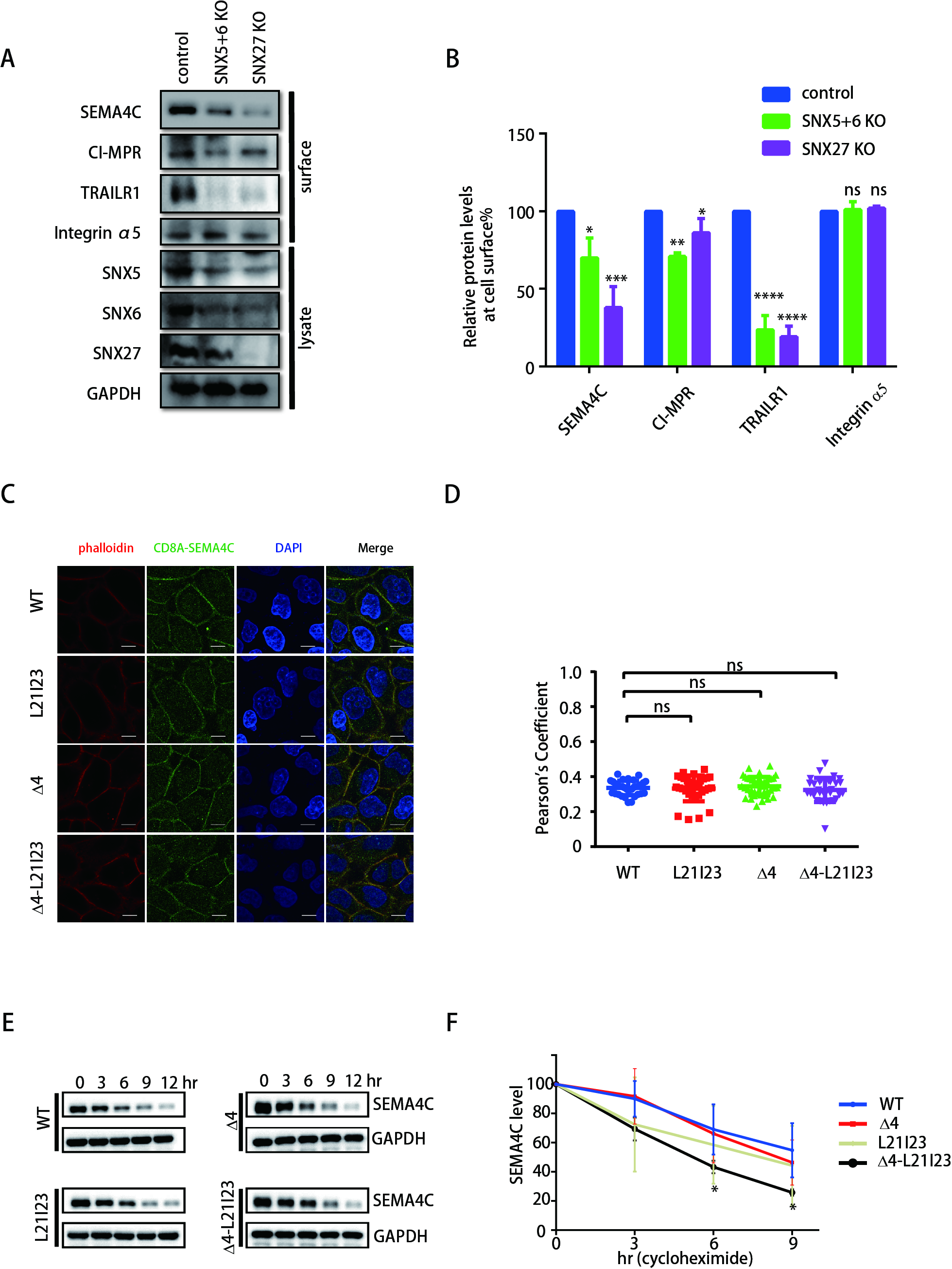

Supplement: S6 Fig — (A) Specific loss of SEMA4C, CI-MPR and TRAILR1 in SNX5/6 KO and SNX27 KO cells. HeLa cells were transfected with CRISPR-Cas9 plasmids targeting SNX27, simultaneous SNX5 and SNX6 (SNX5+6), or empty vector (control). Endogenous protein levels in the polyclonal population were analyzed by immunoblotting, and surface fractions were analyzed for the abundance of the indicated proteins. Integrin 5 is a cargo of neither SNX27 nor SNX-BAR and is used as a control. (B) Immunoblots from A were quantified using ImageJ (n = 3) and normalized to the levels of control cells. Error bars represent standard deviation. P values were compared with WT using one-way ANOVA, Tukey's multiple comparisons test. *P < 0.05; ***P < 0.001; ****P < 0.0001. (C) SBM and/or PDZbm are not required for the internalization of CD8A-SEMA4C. HeLa cells were transiently transfected with plasmids encoding CD8A-SEMA4C WT, L21I23, 4, and 4-L21I23. Cells were incubated with monoclonal anti-human CD8A antibody on ice for 30 min. Unbound antibodies were removed. The internalized CD8A–antibody was detected using Alexa-488 secondary antibodies, with plasma membrane stained with phalloidin (red). Scale bar: 10 μm. (D) Quantification of CD8A/phalloidin colocalization in cells in C. Each dot represents Pearson’s correlation coefficients from one cell. Experiments were triplicated, and the numerical data are included in S1 Data. P values were calculated using one-way ANOVA and Tukey's multiple comparisons test. (E-F) SEMA4C degradation assays. HeLa cells were transfected with WT or mutant Venus–SEMA4C constructs. Forty-eight hours after transfection, cells were treated with the ribosomal inhibitor cycloheximide (50 g/ml) for the indicated time periods (F). The levels of Venus-SEMA4C were analyzed by immunoblotting using an anti-GFP antibody. Amount of Venus-SEMA4C was expressed relative to the amount of GAPDH (loading control) and then normalized to the sample at 0 h. Graph shows the degradation kinetics, with error [file pbio.3000631.s006.tif]

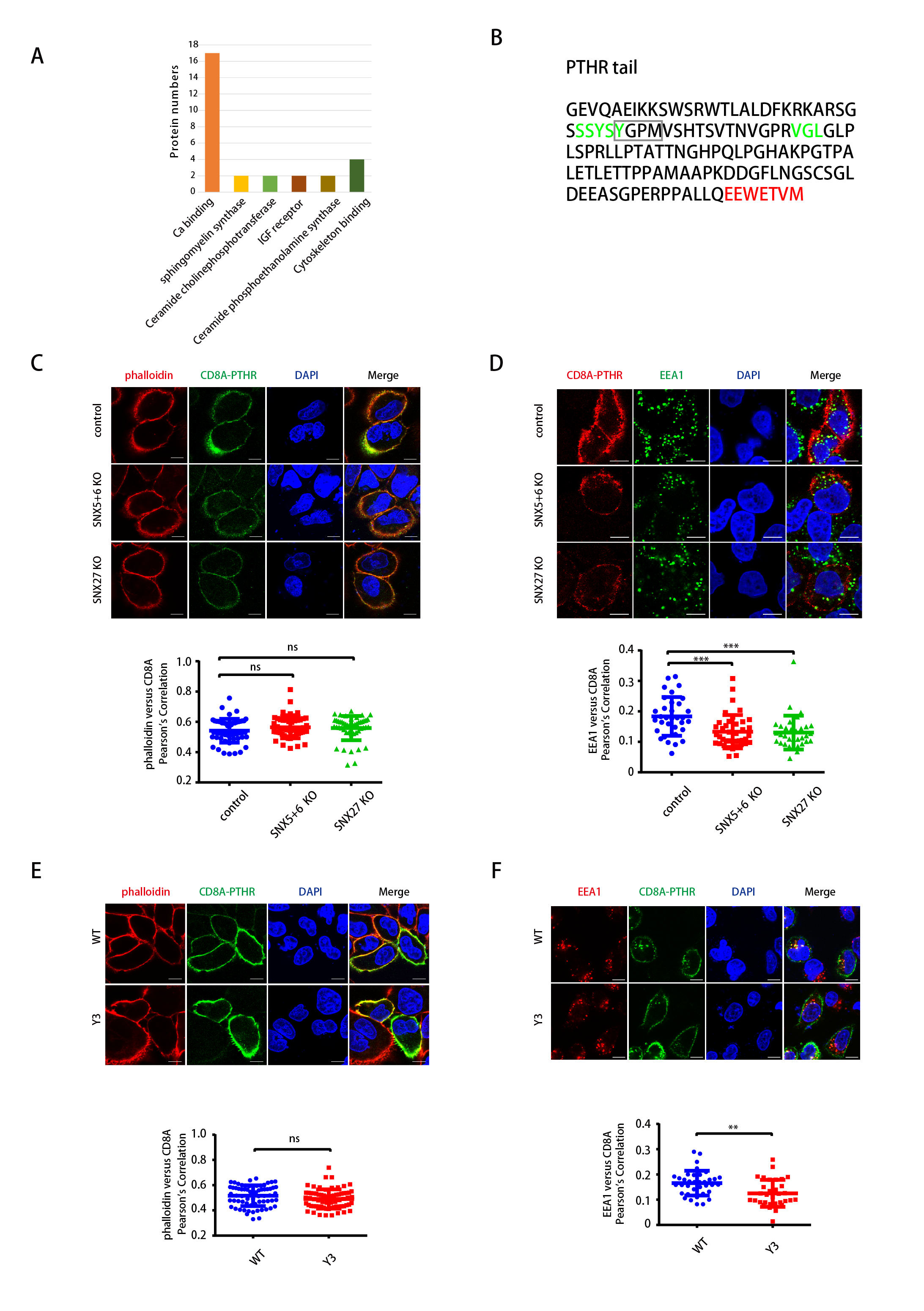

Supplement: S7 Fig — (A) Seventy-one human SBM-containing proteins were analyzed by GO and classified by their molecular functions. The numerical data are included in S1 Data. (B) Amino acid sequences of the cytoplasmic tail of PTHR, and residues in SBM and PDZbm are colored in green and red, respectively. The putative AP-2 binding motif (YGPM) is indicated with a box. (C-D) Control, SNX5+6 KO, and SNX27 KO HeLa cells were transiently transfected with plasmid encoding CD8A-PTHR. Cells were incubated with monoclonal anti-human CD8A antibody on ice for 30 min. Unbound antibodies were then removed by washing with the low-PH buffer, and cells were then chased in serum-free medium at 37°C for 0 h (C) or 1 h (D). The internalized CD8A–antibody was detected with phalloidin (C) or EEA1 (D). The bottom panel shows quantification with CD8A/phalloidin or CD8A/EEA1. Each dot represents Pearson’s correlation coefficients from one cell. Experiments were triplicated, and the numerical data are included in S1 Data. P values were calculated using one-way ANOVA, Tukey's multiple comparisons test. ***P < 0.001. Scale bar: 10 μm. (E-F) HeLa cells were transiently transfected with plasmid encoding CD8A-PTHR WT or Y3 (Y3A). Cells were incubated with monoclonal anti-human CD8A antibody on ice for 30 min. Unbound antibodies were then removed by washing with the low-PH buffer, and cells were then chased in serum-free medium at 37°C for 0 h (C) or 1 h (D). The internalized CD8A–antibody was detected with phalloidin (E) or EEA1 (F). The bottom panel shows quantification with CD8A/phalloidin or CD8A/EEA1. Each dot represents Pearson’s correlation coefficients from one cell. Experiments were triplicated, and the numerical data are included in S1 Data. P values were calculated using one-way ANOVA, Tukey's multiple comparisons test. **P < 0.01. Scale bar: 10 μm. AP-2, adaptor protein complex 2; BAR, Bin/Amphiphysin/Rvs; EEA1, early endosome antigen 1; GO, Gene Ontology; KO, knockout; ns, not significant; PDZbm, PDZ-b [file pbio.3000631.s007.tif]

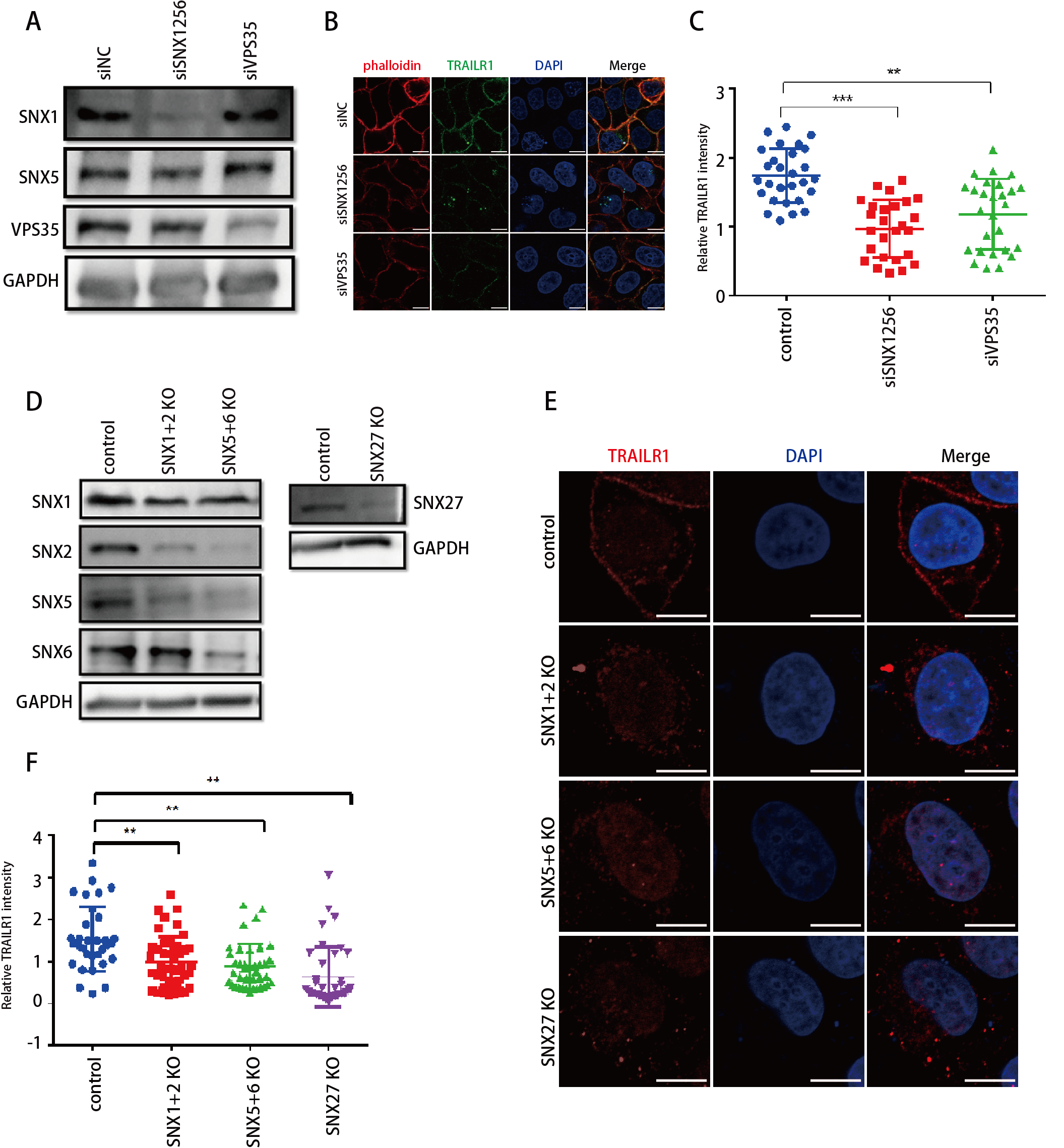

Supplement: S8 Fig — (A) HeLa cells were transiently transfected with control siRNA (“siNC”), or siVPS35, or a combination of siRNAs against SNX1, SNX2, SNX5, and SNX6 (“siSNX1256”). Endogenous protein levels were analyzed by immunoblotting 72 h after the transfection. (B) Immunofluorescence analysis of endogenous TRAILR1 (green) in SNX1256- and VPS35-depleted HeLa cells, with plasma membrane stained with phalloidin (red). (C) Quantification of relative fluorescence intensity of TRAILR1 in B. Experiments were triplicated, and the numerical data are included in S1 Data. Each dot represents average fluorescence intensity from one cell. Scale bar: 10 μm. **P < 0.01; ***P < 0.001. (D) HeLa cells were transfected with CRISPR-Cas9 plasmids targeting SNX27, simultaneous SNX1 and SNX2 (SNX1+2), SNX5 and SNX6 (SNX5+6), or empty vector (control). Endogenous protein levels in the polyclonal population were analyzed by immunoblotting. (E) Immunofluorescence analysis of endogenous TRAILR1 in control, SNX1+2-, SNX5+6-, and SNX27-KO HeLa cells. Scale bar: 10 μm. (F) Quantification of relative fluorescence intensity of TRAILR1 in E. Experiments were triplicated, and the numerical data are included in S1 Data. Each dot represents average fluorescence intensity from one cell. **P < 0.01. BAR, Bin/Amphiphysin/Rvs; KO, knockout; siRNA, small interfering RNA; SNX, Sorting Nexin family; TRAILR1, TNF-related apoptosis-inducing ligand receptor 1; VPS, vacuolar protein sorting. (TIF) [file pbio.3000631.s008.tif]

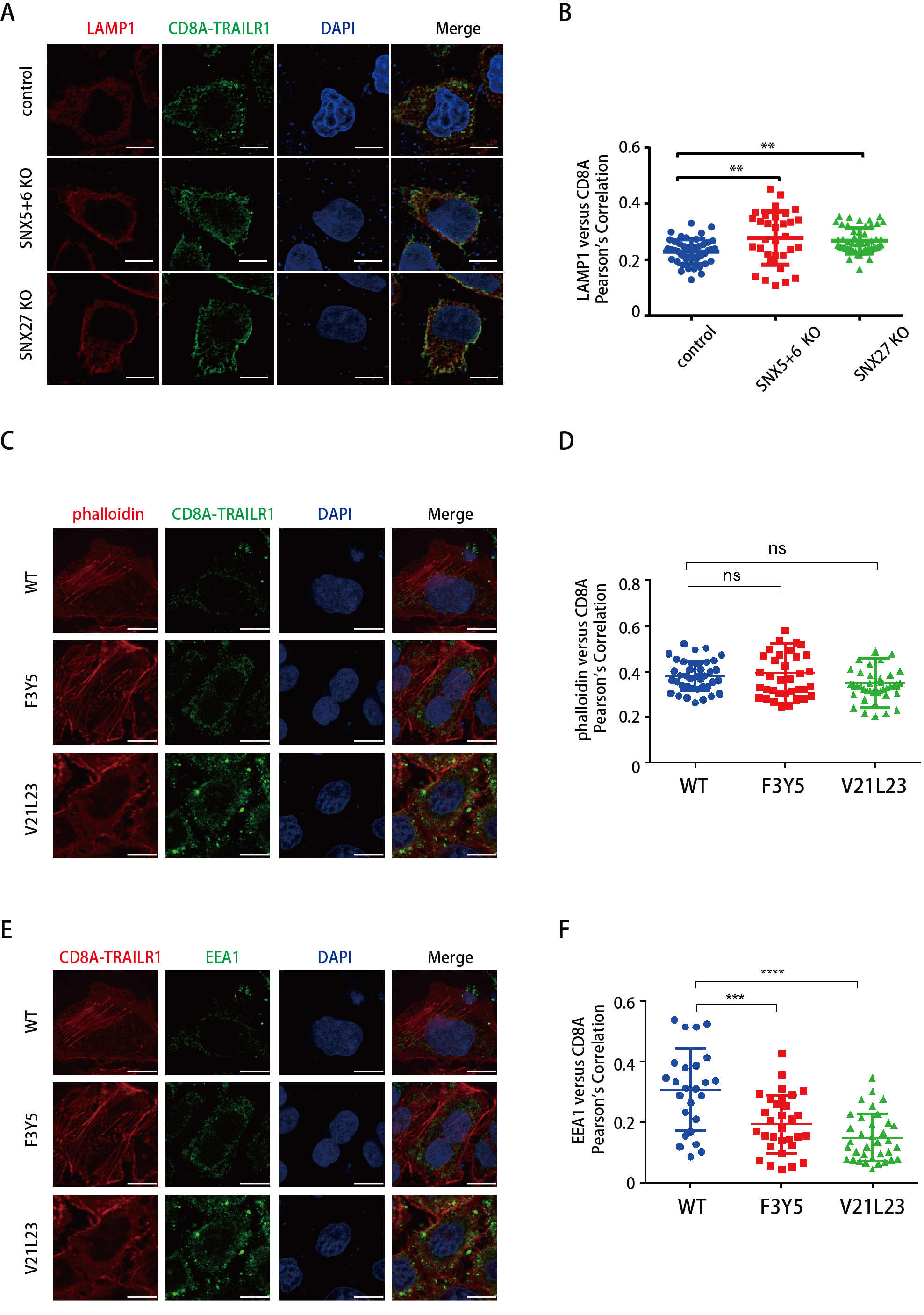

Supplement: S9 Fig — (A) Control, SNX5+6-KO, and SNX27-KO HeLa cells were transiently transfected with plasmids encoding CD8A-TRAILR1. Cells were incubated with monoclonal anti-human CD8A antibody on ice for 30 min. Unbound antibodies were removed, and the internalization of antibody-bound CD8A was carried out in DMEM at 37°C for 3 h. The internalized CD8A–antibody was detected using Alexa-488 secondary antibodies, with lysosomes stained with LAMP1 (red). Scale bar: 10 μm. (B) Quantification of CD8A/LAMP1 colocalization in cells in A. Each dot represents Pearson’s correlation coefficients from one cell. Experiments were triplicated, and the numerical data are included in S1 Data. P values were calculated using one-way ANOVA, Tukey's multiple comparisons test. Scale bar: 10 μm. **P < 0.01. (C) HeLa cells were transiently transfected with plasmids encoding CD8A-TRAILR1 WT, F3Y5, and V21L23. Cells were incubated with monoclonal anti-human CD8A antibody on ice for 30 min. Unbound antibodies were removed. The internalized CD8A–antibody was detected using Alexa-488 secondary antibodies, with plasma membrane stained with phalloidin (red). (D) Quantification of CD8A/phalloidin colocalization in cells in C. Each dot represents Pearson’s correlation coefficients from one cell. Experiments were triplicated, and the numerical data are included in S1 Data. P values were calculated using one-way ANOVA, Tukey's multiple comparisons test. (E) HeLa cells were transiently transfected with plasmids encoding CD8A-TRAILR1 WT, F3Y5, and V21L23. Cells were incubated with monoclonal anti-human CD8A antibody on ice for 30 min. Unbound antibodies were removed, and the internalization of antibody-bound CD8A was carried out in DMEM at 37°C for 1 h. The internalized CD8A–antibody was detected using Alexa-546 secondary antibodies, with endosomes stained with anti-EEA1 antibody (green). (F) Quantification of CD8A/EEA1 colocalization in cells in E. Each dot represents Pearson’s correlation coefficients from one cell. [file pbio.3000631.s009.tif]
