# Supplementary material for: Mechanism of cargo recognition by retromer-linked SNX-BAR proteins
Source: PLoS Biol. 2020 Mar 9;18(3):e3000631. doi: 10.1371/journal.pbio.3000631 (PMC7082075; doi:10.1371/journal.pbio.3000631)
Supplement: S2 Table — (a) A list of surface proteins affected by the loss of SNX-BARs was reported by Simonetti and colleagues [48]. (b) A list of surface proteins affected by the loss of SNX27 or enriched in the SNX27 interactome was reported by Steinberg and colleagues [14]. (c). A list of surface proteins affected by the loss of VPS35 was reported by Steinberg and colleagues [14]. (d) Cargo proteins with sequence identity of 40% or higher were listed. BAR, Bin/Amphiphysin/Rvs; SNX, Sorting Nexin family; VPS, vacuolar protein sorting. (DOCX) [file pbio.3000631.s011.docx]

**S2 Table Shared cargos affected by loss of SNX-BARs, SNX27, or Retromer**

| **SNX-BARs^a^** | **SNX27^b^** | **VPS35^c^** | **Identity^d^** |
| --- | --- | --- | --- |
| ABCC1 | MRP1 |  | 46.1% |
| LRP8 | LRP8 |  | 100% |
| SLCO3A1 | SLCO3A1 | SLCO3A1 | 100% |
| SLC9A6 |  | SLC9A6 | 100% |
| SLC39A8 | SLC39A14 | SLC39A14 | 45% |
| MUC16 | MUC16 |  | 100% |
| SLC22A5 | SLC22A5 | SLC22A5 | 100% |
| SLC6A6 | SLC6A6 |  | 100% |
| ATP11A | ATP11A |  | 100% |
| PLXNA3 | PLXNA2 | PLXNA2 | 76.8% |
| ITGA5 |  | ITGA5 | 100% |
| SLC16A3 | MCT1 | MCT1 | 42.6% |
| ADAM17 | ADAM17 | ADAM17 | 100% |
| HS6ST2 |  | HS6ST2 | 100% |
| PODXL | PODXL | PODXL | 100% |
| Robo1 | Robo1 |  | 100% |
| CSPG4 |  | CSPG4 | 100% |
| CELSR1 | CELSR1 |  | 100% |
| PTK7 |  | PTK7 | 100% |
| SLC7A1 | SLC7A1 | SLC7A1 | 100% |
| CELSR2 | CELSR2 |  | 100% |
| GGT1 | GGT1 | GGT1 | 100% |
| SEMA4C | SEMA4C |  | 100% |
| RFT1 | GPR172A |  | 86.1% |

a. A list of surface proteins affected by the loss of SNX-BARs was reported by Simonetti et al ^[1](#_ENREF_1)^.

b. A list of surface proteins affected by the loss of SNX27 or enriched in the SNX27 interactome was reported by Steinberg et al ^[2](#_ENREF_2)^.

c. A list of surface proteins affected by the loss of VPS35 was reported by Steinberg et al [^2^](#_ENREF_2).

d. Cargo proteins with sequence identity of 40% or higher were listed.
