# Supplementary material for: Mechanism of cargo recognition by retromer-linked SNX-BAR proteins
Source: PLoS Biol. 2020 Mar 9;18(3):e3000631. doi: 10.1371/journal.pbio.3000631 (PMC7082075; doi:10.1371/journal.pbio.3000631)
Supplement: S3 Table — (DOCX) [file pbio.3000631.s012.docx]

**S3 Table DNA Constructs Used in this Study.**

| **Construct name** | **Description** | **Source or reference** |
| --- | --- | --- |
| **Retromer** | | |
| VPS35 | GST-Tev-VPS35-full length, GST-Tev finally removed | (Jia et al, 2012) |
| VPS26 | His6-Tev-VPS26A (9-327), His6-Tev finally removed | (Jia et al, 2012) |
| VPS29 | His6-Tev-VPS29-full length, His6-Tev finally removed | (Jia et al, 2012) |
| **CI-MPR** | | |
| CI-MPR cytoplasmic tail_1-164 | GST-Tev-CI-MPR cytoplasmic tail_1-164 | This study |
| CI-MPR cytoplasmic tail_1-75 | GST-Tev-CI-MPR cytoplasmic tail_1-75 | This study |
| CI-MPR cytoplasmic tail_1-56 | GST-Tev-CI-MPR cytoplasmic tail_1-56 | This study |
| CI-MPR cytoplasmic tail_21-56 | GST-Tev-CI-MPR cytoplasmic tail_21-56 | This study |
| CI-MPR cytoplasmic tail_21-51 | GST-Tev-CI-MPR cytoplasmic tail_21-51 | This study |
| CI-MPR cytoplasmic tail_21-48 | GST-Tev-CI-MPR cytoplasmic tail_21-48 | This study |
| CI-MPR cytoplasmic tail_21-48_V1 | GST-Tev-CI-MPR cytoplasmic tail_21-48_V1 | This study |
| CI-MPR cytoplasmic tail_21-48_Y3 | GST-Tev-CI-MPR cytoplasmic tail_21-48_Y3 | This study |
| CI-MPR cytoplasmic tail_21-48_Y5 | GST-Tev-CI-MPR cytoplasmic tail_21-48_Y5 | This study |
| CI-MPR cytoplasmic tail_21-48_K7 | GST-Tev-CI-MPR cytoplasmic tail_21-48_K7 | This study |
| CI-MPR cytoplasmic tail_21-48_W21M23 | GST-Tev-CI-MPR cytoplasmic tail_21-48_W21M23 | This study |
| CI-MPR cytoplasmic tail_21-44 | GST-Tev-CI-MPR cytoplasmic tail_21-44 | This study |
| CI-MPR cytoplasmic tail_21-48_ΔLoop5 | GST-Tev-CI-MPR cytoplasmic tail_21-48_ΔLoop5 | This study |
| CI-MPR cytoplasmic tail_21-48_ΔLoop8 | GST-Tev-CI-MPR cytoplasmic tail_21-48_ΔLoop8 | This study |
| CI-MPR cytoplasmic tail_21-48_ΔLoop10 | GST-Tev-CI-MPR cytoplasmic tail_21-48_ΔLoop10 | This study |
| Venus-CI-MPR | GLP-Venus-TM-CI-MPR cytoplasmic tail_1-164 | (Simonetti et al, 2017) |
| Venus-CI-MPR Y3 | GLP-Venus-TM-CI-MPR cytoplasmic tail_1-164_ Y3 | This study |
| Venus-CI-MPR Y5 | GLP-Venus-TM-CI-MPR cytoplasmic tail_1-164_ Y5 | This study |
| Venus-CI-MPR Y3Y5 | GLP-Venus-TM-CI-MPR cytoplasmic tail_1-164_ Y3Y5 | This study |
| Venus-CI-MPR W21M23 | GLP-Venus-TM-CI-MPR cytoplasmic tail_1-164_ W21M23 | This study |
| **SNXs** | | |
| SNX1 | MBP-Tev-SNX1-full length | (Yong et al., 2018) |
| SNX1ΔN | MBP-Tev-SNX1(139-C) | (Yong et al., 2018) |
| SNX1ΔN | MBP-Tev-SNX1(139-C)_K214E | This study |
| SNX3 | MBP-Tev-SNX3-full length | (Lucas et al, 2016) |
| SNX5 | His_6_-sumo-SNX5-full length | This study |
| HA-SNX5 | HA-SNX5-full length | This study |
| SNX5^PX^ | MBP-Tev-SNX5 (20-180) | This study |
| SNX5^PX^ _E129A | MBP-Tev-SNX5 (20-180)_ E129A | This study |
| SNX5^PX^ _Y132D | MBP-Tev-SNX5 (20-180)_Y132D | This study |
| SNX5^PX^ _L133A | MBP-Tev-SNX5 (20-180)_L133A | This study |
| SNX5^PX^ _F136D | MBP-Tev-SNX5 (20-180)_F136D | This study |
| SNX5^PX^ _E144A | MBP-Tev-SNX5 (20-180)_E144A | This study |
| SNX3 | MBP-Tev-SNX3-full length | (Lucas et al, 2016) |
| SNX5 | His_6_-sumo-SNX5-full length | This study |
| HA-SNX5 | HA-SNX5-full length | This study |
| SNX6 | His-sumo-SNX6-full length | (Yong et al., 2018) |
| SNX6_F149D | His-sumo-SNX6-full length_F149D | This study |
| SNX6^PX^ | MBP-Tev-SNX6 (33-187) | This study |
| SNX27^PDZ^ | GST-Tev-SNX27 (43-136) | This study |
| SNX32^PX^ | GST-Tev-SNX32（17-175） | This study |
| HA-YFP-SNX2 | HA-YFP-SNX2-full length | This study |
| HA-YFP-SNX6 | HA-YFP-SNX6-full length | This study |
| FLAG-SNX27 | FLAG-SNX27-full length | This study |
| **SEMA4C** | | |
| SEMA4C cytoplasmic tail_1-149 | GST-TEV-SEMA4C cytoplasmic tail_1-149 | This study |
| SEMA4C cytoplasmic tail_1-149_Δ4 | GST-TEV-SEMA4C cytoplasmic tail_1-149_Δ4 | This study |
| SEMA4C cytoplasmic tail_1-149_Y3Y5 | GST-TEV-SEMA4C cytoplasmic tail_1-149_Y3Y5 | This study |
| SEMA4C cytoplasmic tail_1-149_Δ4_Y3Y5 | GST-TEV-SEMA4C cytoplasmic tail_1-149_Δ4_Y3Y5 | This study |
| SEMA4C cytoplasmic tail_47-71 | GST-TEV-SEMA4C cytoplasmic tail_47-71 | This study |
| SEMA4C cytoplasmic tail_47-71_Y3Y5 | GST-TEV-SEMA4C cytoplasmic tail_47-71_Y3Y5 | This study |
| SEMA4C cytoplasmic tail_47-71_L21I23 | GST-TEV-SEMA4C cytoplasmic tail_47-71_L21I23 | This study |
| SEMA4C cytoplasmic tail_47-65 | GST-TEV-SEMA4C cytoplasmic tail_47-65 | This study |
| Venus-SEMA4C | GLP-Venus- TM-SEMA4C cytoplasmic tail_1-149 | This study |
| Venus-SEMA4C L21I23 | GLP-Venus-TM-SEMA4C cytoplasmic tail_1-149_ L21I23 | This study |
| Venus-SEMA4C-Δ4 | GLP-Venus-TM-SEMA4C cytoplasmic tail_1-149_Δ4 | This study |
| Venus-SEMA4C-Δ4-L21I23 | GLP-Venus-TM-SEMA4C cytoplasmic tail_1-149_ Δ4L21I23 | This study |
| CD8A-SEMA4C | CD8A-SEMA4C- cytoplasmic tail_1-149 | Based on (Seaman, 2004) |
| CD8A-SEMA4C-L21I23 | CD8A-SEMA4C cytoplasmic tail_1-149_ L21I23 | This study |
| CD8A-SEMA4C-Δ4 | CD8A-SEMA4C cytoplasmic tail_1-149_Δ4 | This study |
| CD8A-SEMA4C-Δ4-L21I23 | CD8A-SEMA4C cytoplasmic tail_1-149_ Δ4L21I23 | This study |
| **IGF1R and INSR** | | |
| IGF1R cytoplasmic tail | GST-TEV-IGF1R cytoplasmic tail(960-1367) | This study |
| IGF1R cytoplasmic tail_Y3Y5 | GST-TEV-IGF1R cytoplasmic tail(960-1367)_Y3Y5 | This study |
| IGF1R cytoplasmic tail_Y5H | GST-TEV-IGF1R cytoplasmic tail(960-1367)_Y5H | This study |
| INSR cytoplasmic tail | GST-TEV-INSR cytoplasmic tail(980-1382) | This study |
| INSR cytoplasmic tail_H5Y | GST-TEV-INSR cytoplasmic tail(980-1382)_H5Y | This study |
| **PTHR** | | |
| PTHR cytoplasmic tail | GST-TEV-PTHR cytoplasmic tail(464-593) | This study |
| PTHR cytoplasmic tail_Y3Y5 | GST-TEV-PTHR cytoplasmic tail(464-593)_Y3Y5 | This study |
| PTHR cytoplasmic tail_V21L23 | GST-TEV-PTHR cytoplasmic tail(464-593)_V21L23 | This study |
| CD8A-PTHR | CD8A-TM-PTHR cytoplasmic tail(464-593) | This study |
| CD8A-PTHR Y3 | CD8A -TM-PTHR cytoplasmic tail(464-593)_Y3 | This study |
| **TRAILR1** | | |
| TRAILR1 cytoplasmic tail | GST-TEV-TRAILR1 cytoplasmic tail(263-468) | This study |
| TRAILR1 cytoplasmic tail_F3Y5 | GST-TEV-TRAILR1 cytoplasmic tail(263-468)_F3Y5 | This study |
| TRAILR1 cytoplasmic tail_V21L23 | GST-TEV-TRAILR1 cytoplasmic tail(263-468)_V21L23 | This study |
| Venus-TRAILR1 | GLP-Venus- TM-TRAILR1 cytoplasmic tail | This study |
| Venus-TRAILR1 F3Y5 | GLP-Venus- TM-TRAILR1 cytoplasmic tail_F3Y5 | This study |
| Venus-TRAILR1 V21L23 | GLP-Venus- TM-TRAILR1 cytoplasmic tail_V21L23 | This study |
| CD8A-TRAILR1 | CD8A- TM-TRAILR1 cytoplasmic tail | This study |
| CD8A-TRAILR1 F3Y5 | CD8A- TM-TRAILR1 cytoplasmic tail_F3Y5 | This study |
| CD8A-TRAILR121L23 | CD8A- TM-TRAILR1 cytoplasmic tail_V21L23 | This study |
| GST-TRAIL | GST-TRAIL-Full length | (Kong et al,2019) |
| **OTHER** | | |
| STIMA cytoplasmic tail | GST-TEV-STIMA cytoplasmic tail(215-294) | This study |
| CED-1 cytoplasmic tail | GST-TEV-CED-1 cytoplasmic tail(932-980) | This study |
| JAM3 cytoplasmic tail | GST-TEV-JAM3 cytoplasmic tai(263-310) | This study |
| IncE | MBP-TEV-IncE(109-132) | (Sun et al, 2017) |
