# Supplementary material for: Mechanism of cargo recognition by retromer-linked SNX-BAR proteins
Source: PLoS Biol. 2020 Mar 9;18(3):e3000631. doi: 10.1371/journal.pbio.3000631 (PMC7082075; doi:10.1371/journal.pbio.3000631)
Supplement: S4 Table — (DOCX) [file pbio.3000631.s013.docx]

**S4 Table Summary of Antibodies Used in this Study**

| Antibody | Source | Catalog# |
| --- | --- | --- |
| GFP | Proteintech | 50430-2-AP |
| GAPDH | Proteintech | 0494-1-AP |
| SNX1 | BD | 611482 |
| SNX2 | BD | 611308 |
| SNX5 | Absin | abs121839 |
| SNX6 | Absin | abs122003 |
| SNX6 | Santa Cruz | sc-365965 |
| FLAG | Proteintech | 20543-1-AP |
| HA | Proteintech | 51064-2-AP |
| GST(B-14) | Santa Cruz | SC-138 |
| TRAILR1 | CST | D9S1R |
| TRAILR1(ectodomain) | Aviva | Clone DR-4-02 |
| VPS35 | Abcam | ab10099-100 |
| CD8A | Thermo Fisher | 14-0086-80 |
| PARP | Proteintech | 22999-1-AP |
| EEA1 | CST | 3288 |
| MBP | Proteintech | 15089-1-AP |
| LAMP1 | Abcam | 24170 |
