# Supplementary material for: Mechanism of cargo recognition by retromer-linked SNX-BAR proteins
Source: PLoS Biol. 2020 Mar 9;18(3):e3000631. doi: 10.1371/journal.pbio.3000631 (PMC7082075; doi:10.1371/journal.pbio.3000631)

Fig 1

Loading orders and samples are indicated as described in manuscript.

Fig 1A

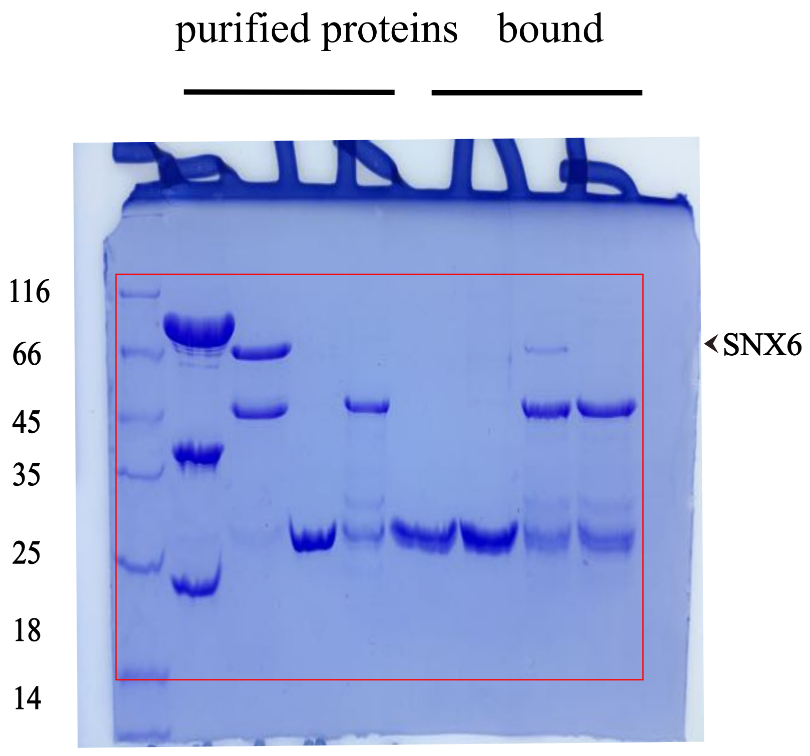

Fig 1B

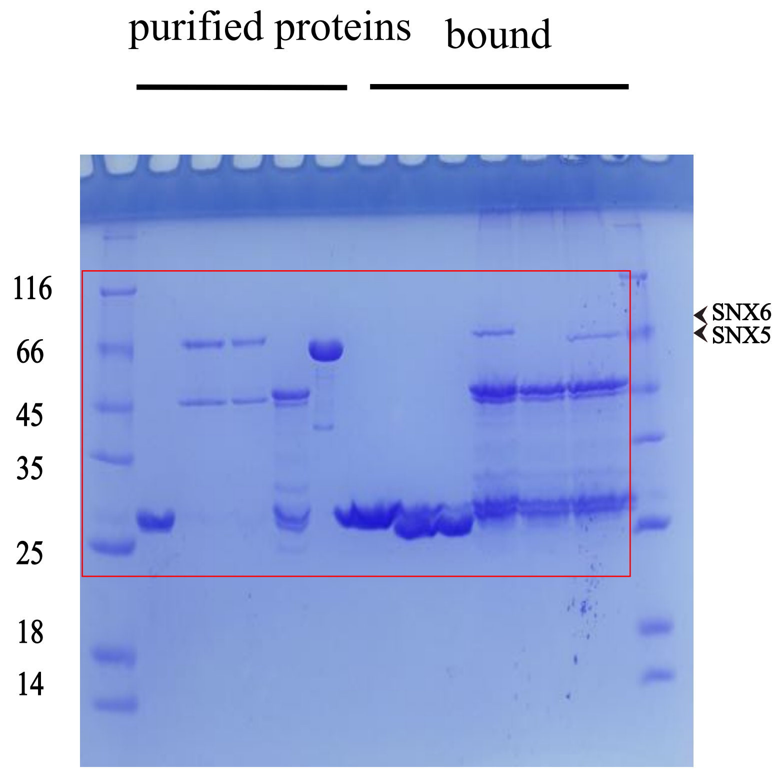

Fig 1C

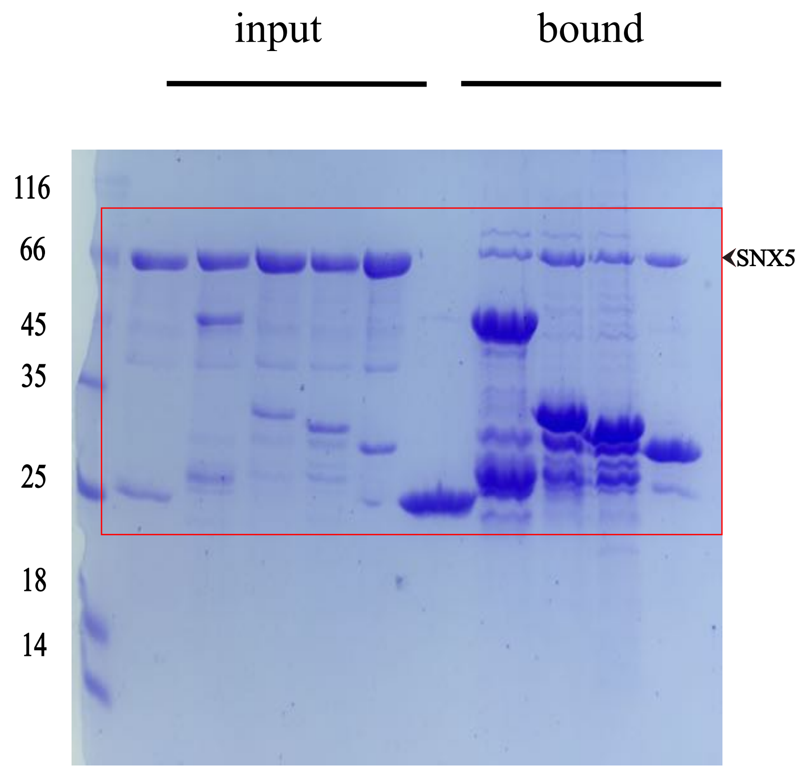

Fig 2

Fig 2E

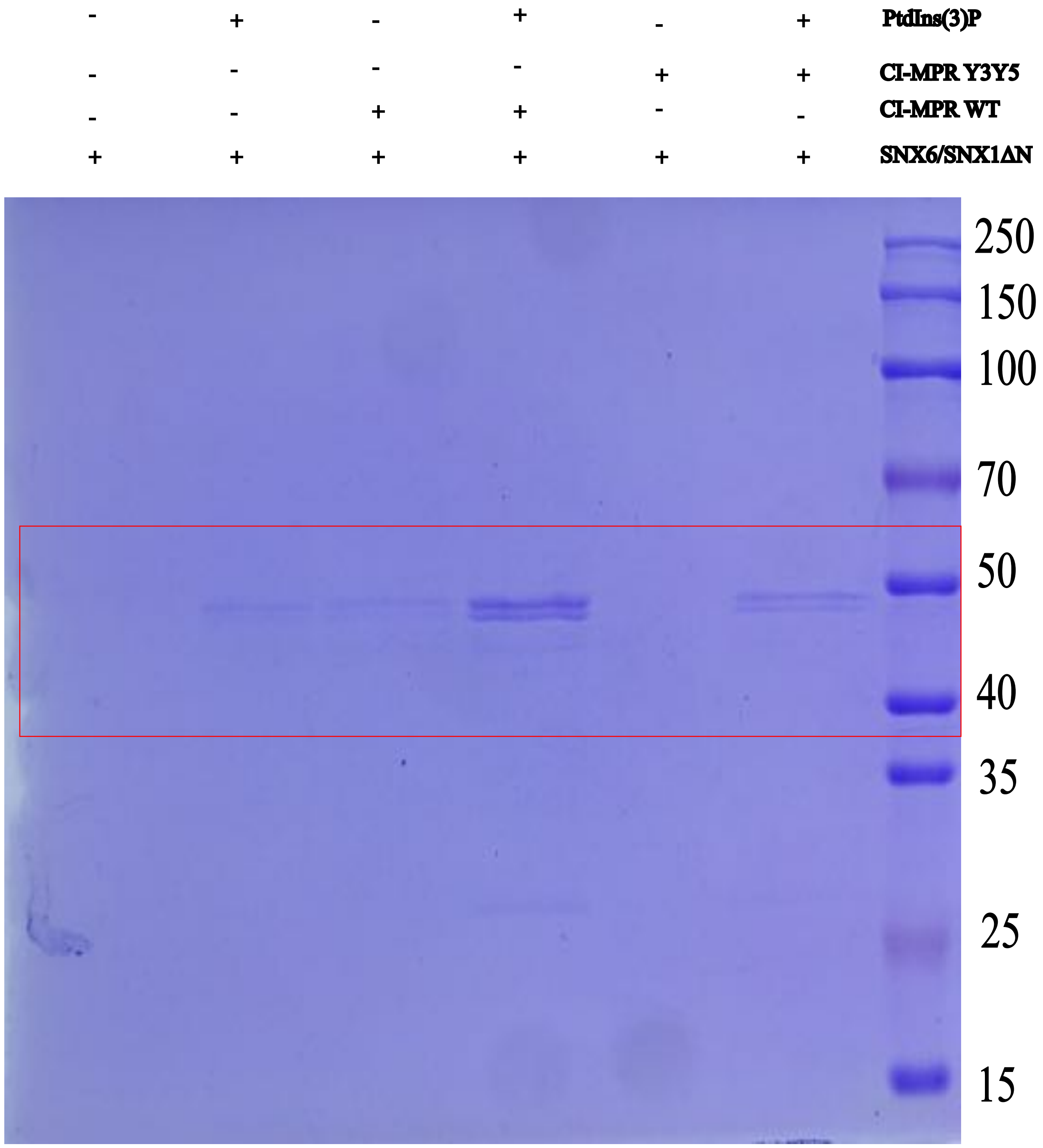

Fig 2F

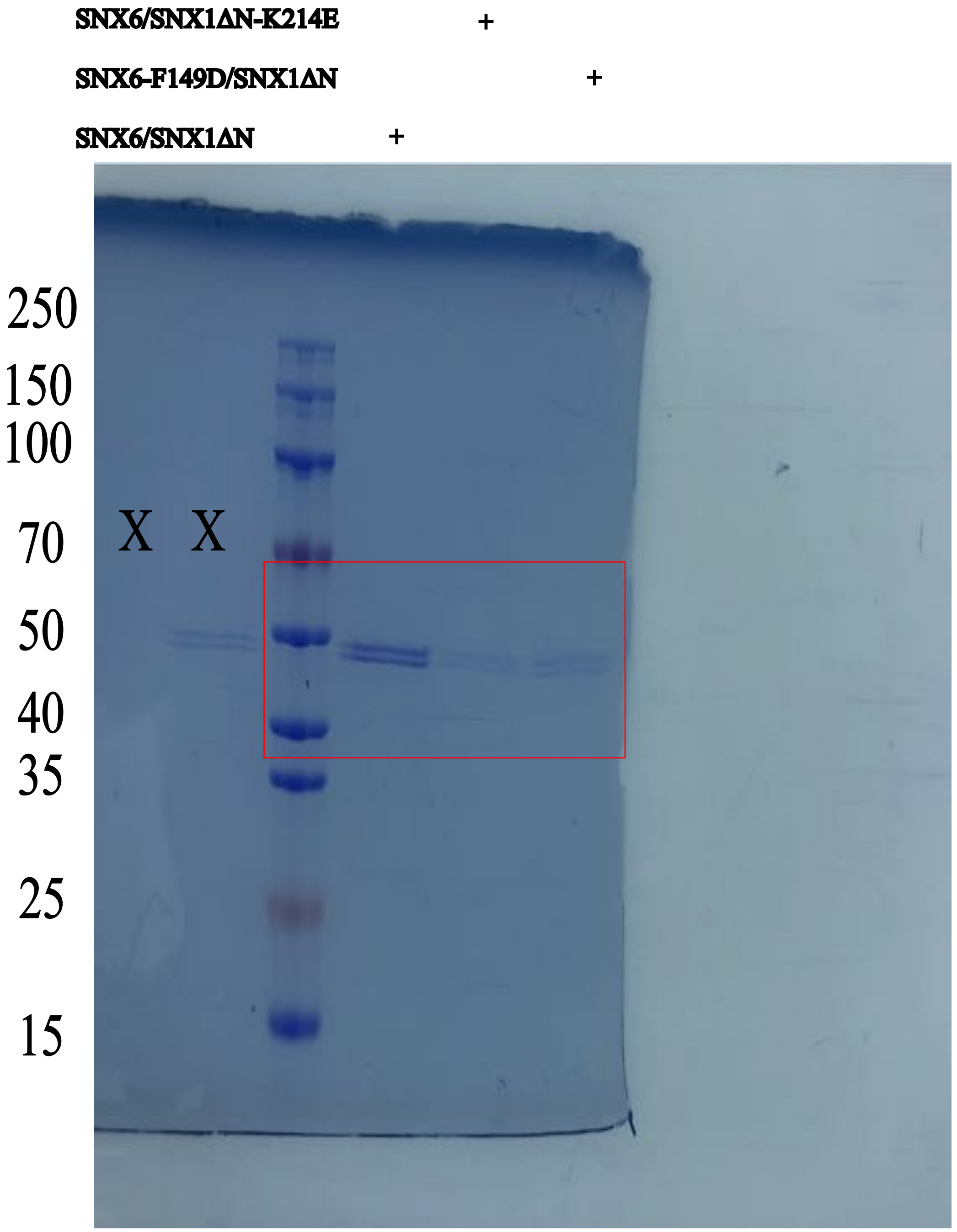

Fig 3

Fig 3B

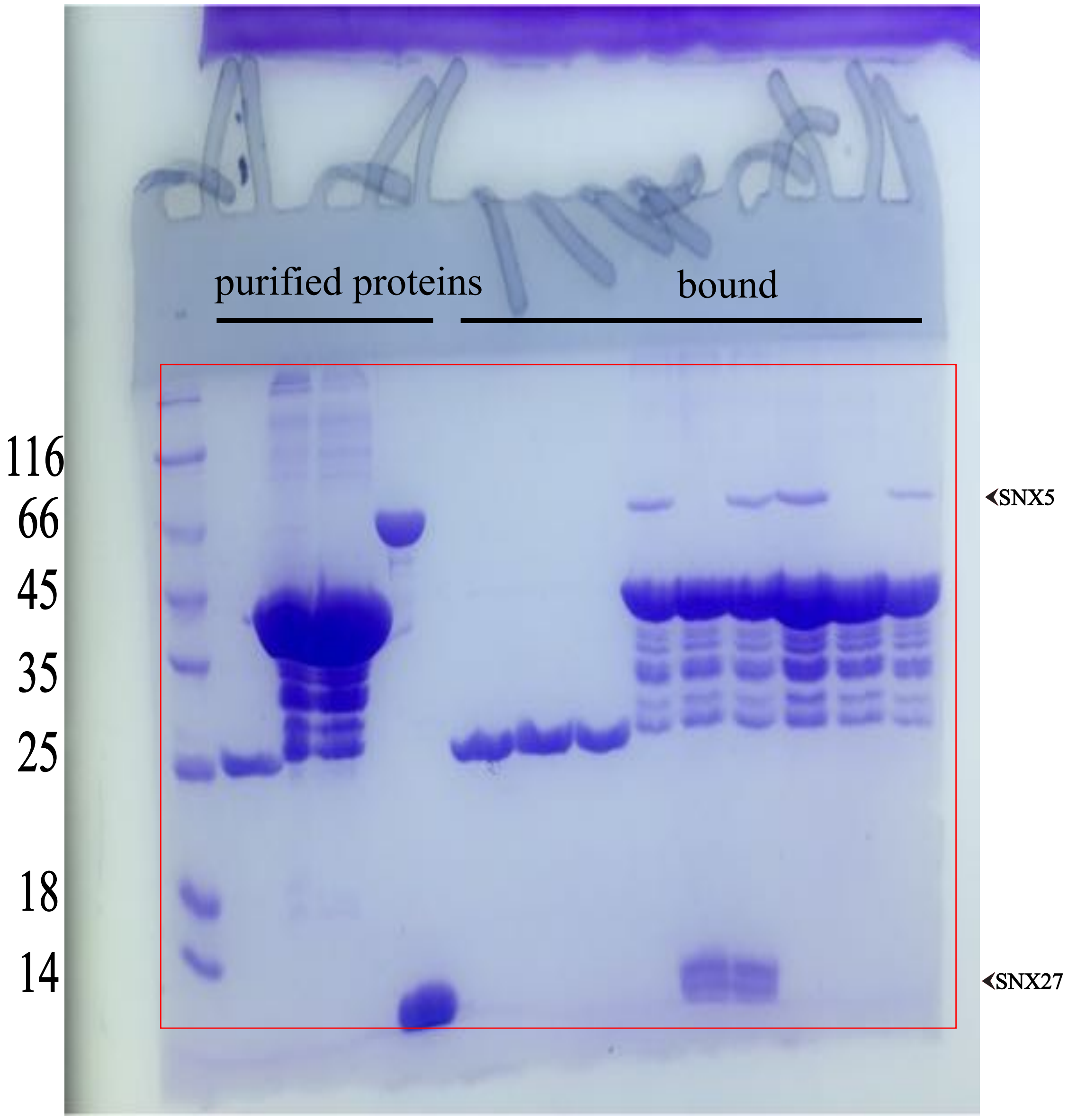

Fig 3C

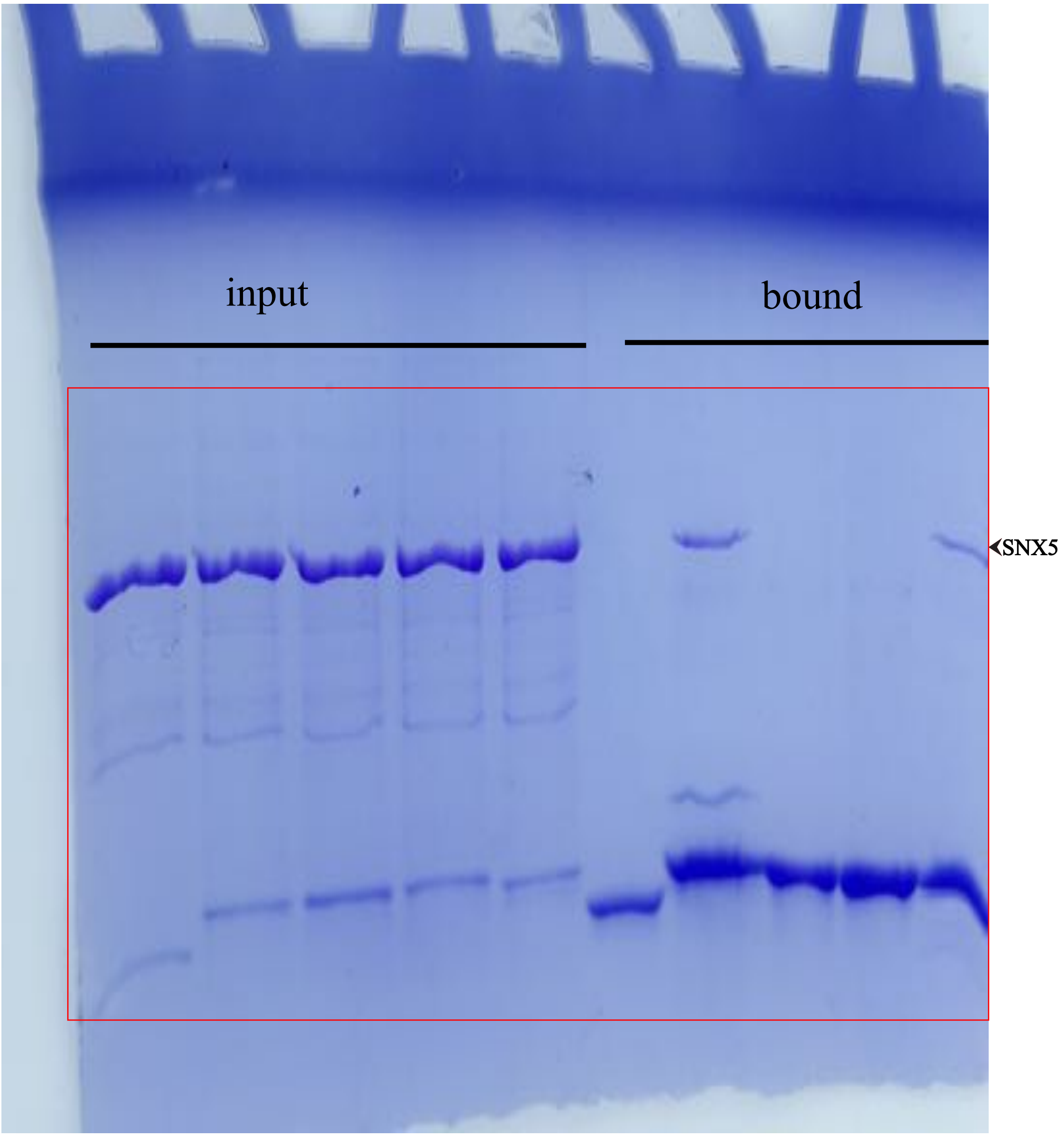

Fig 4

Fig 4B

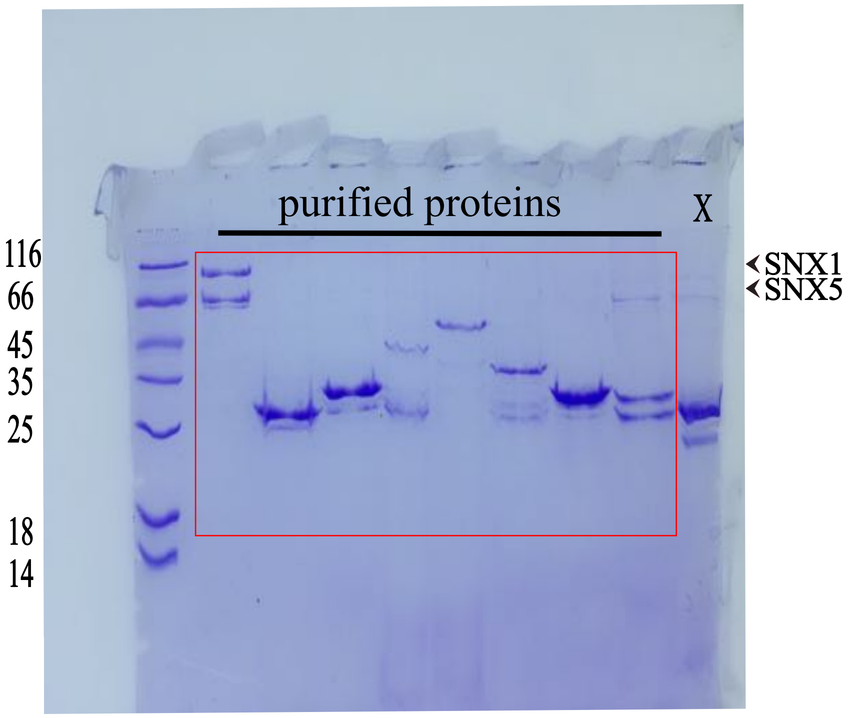

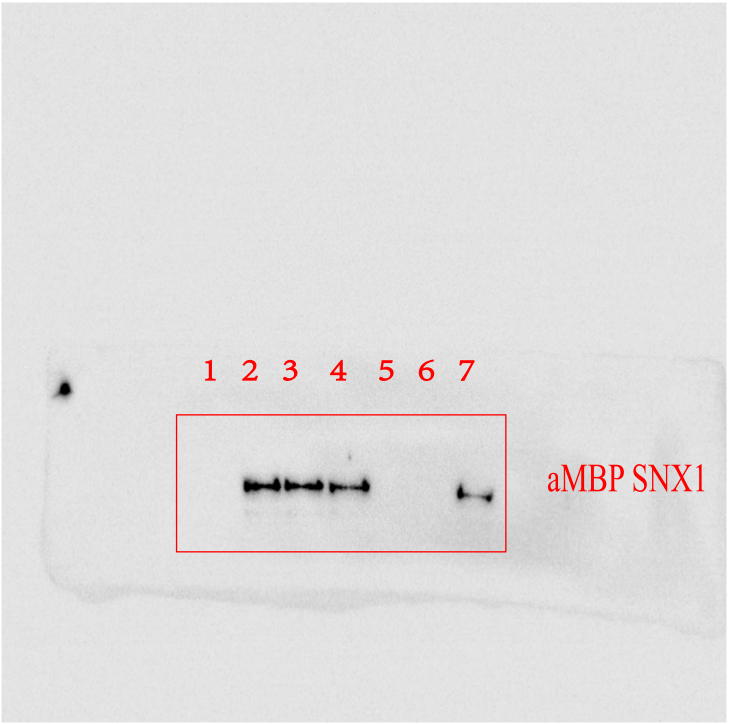

Fig 4C

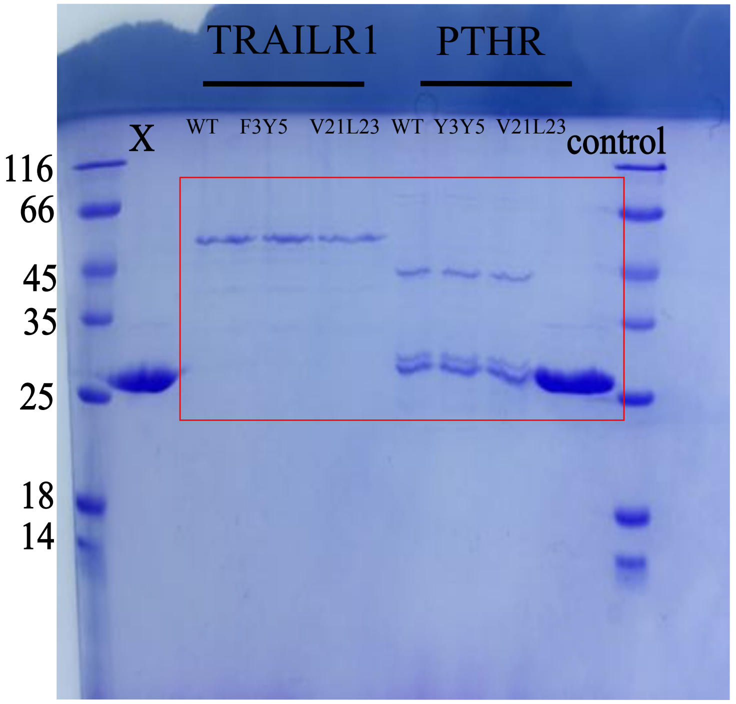

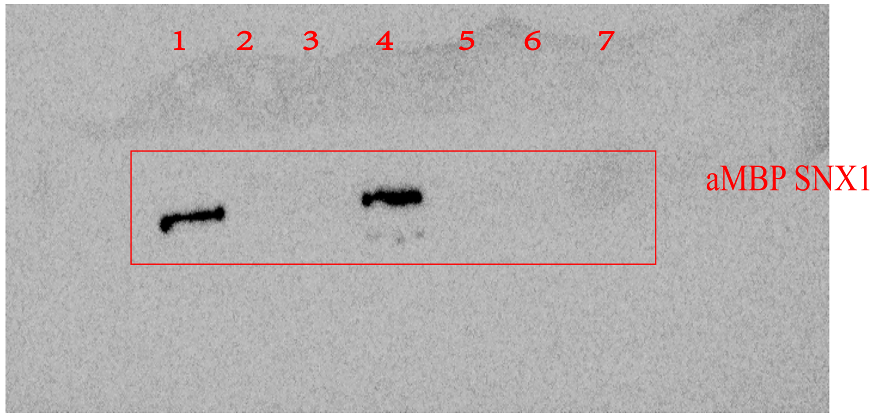

Fig 5D

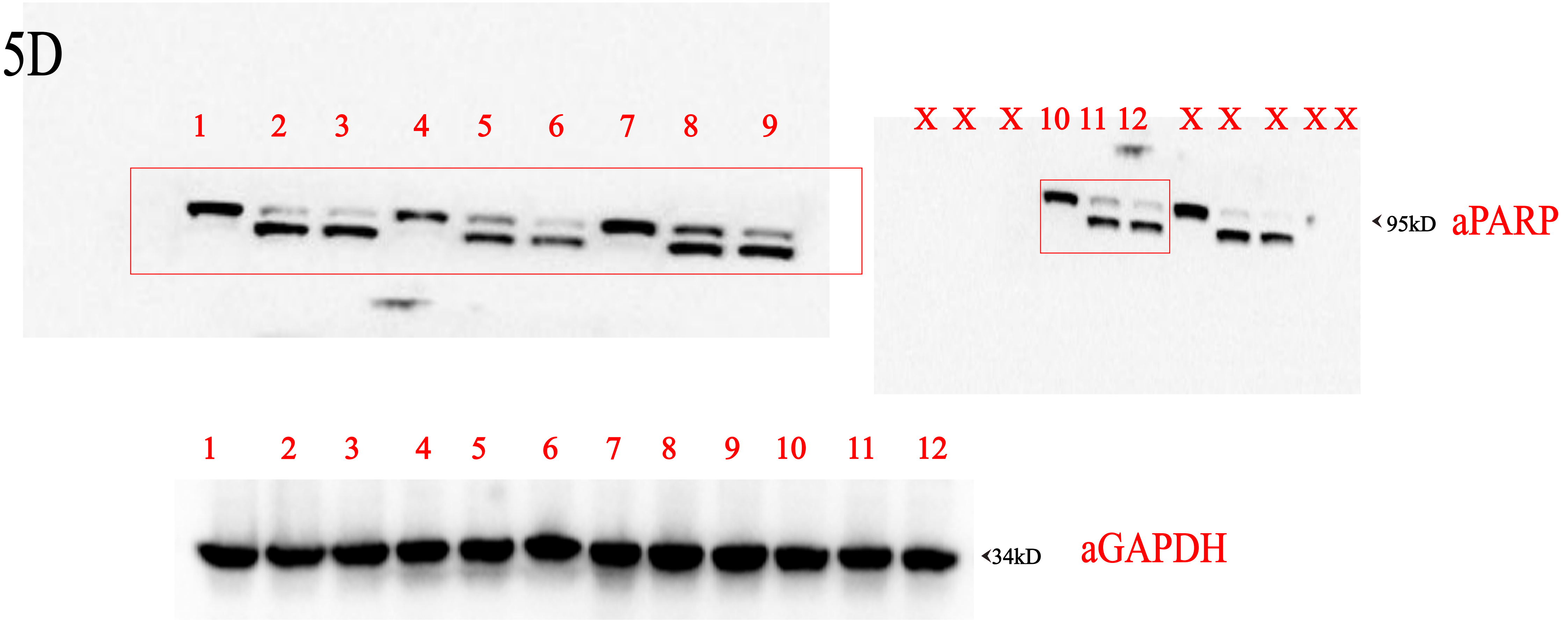

S1A Fig

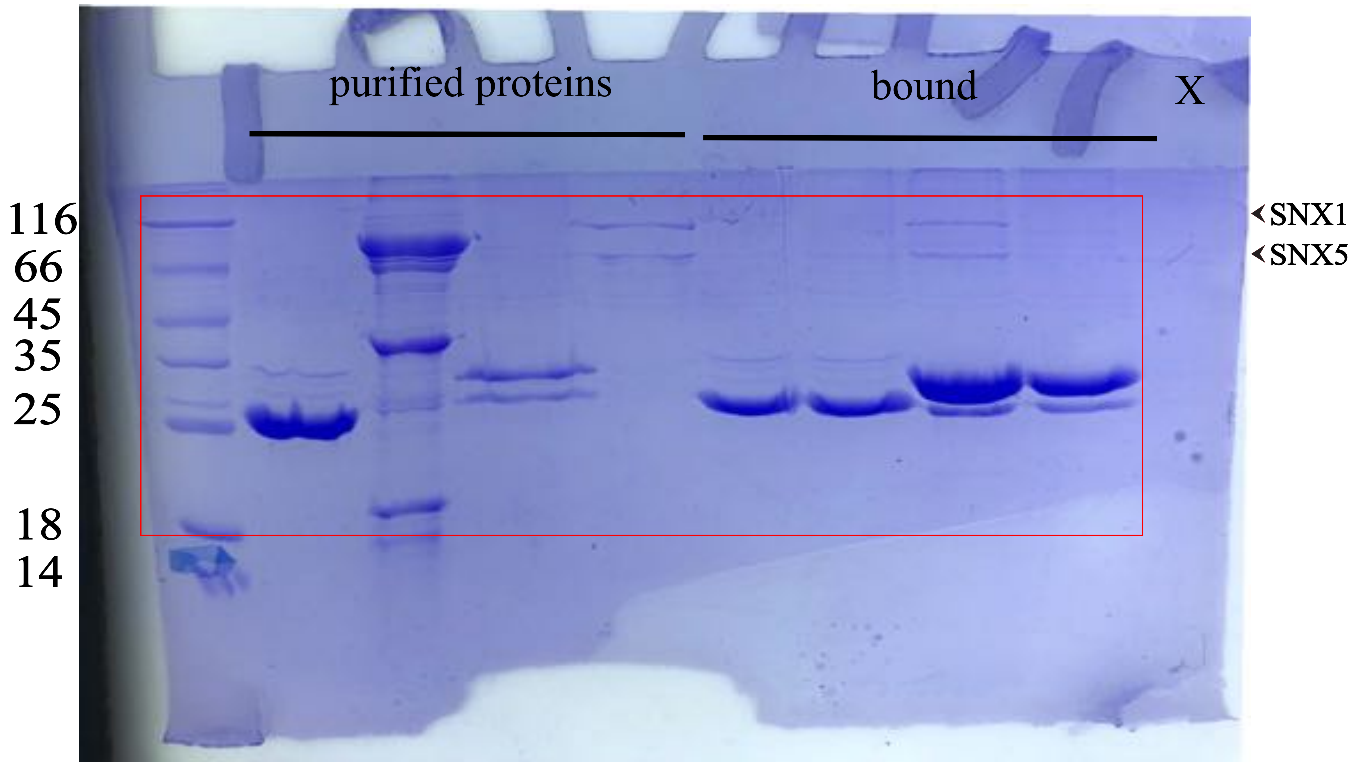

S3 Fig

S3B Fig

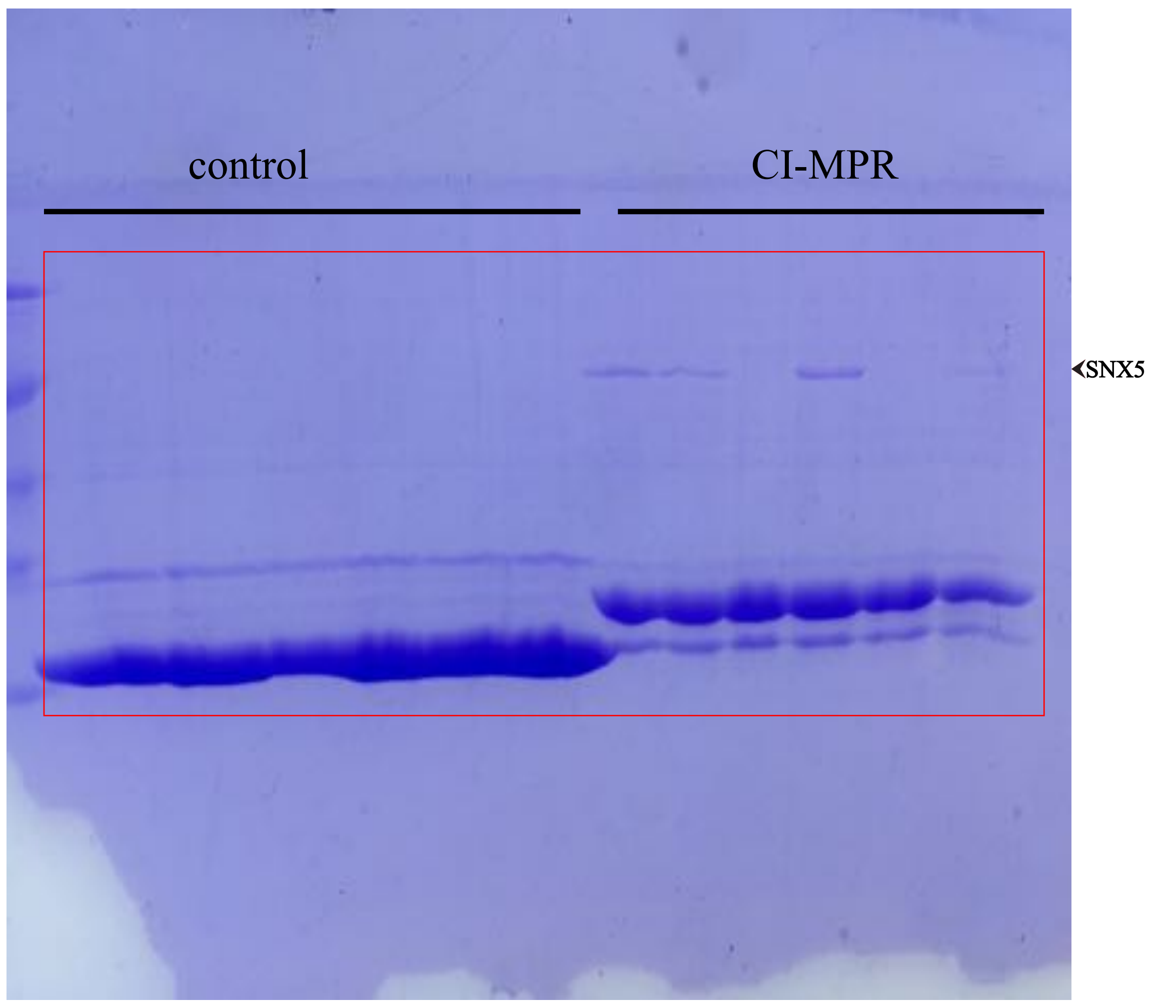

S3C Fig

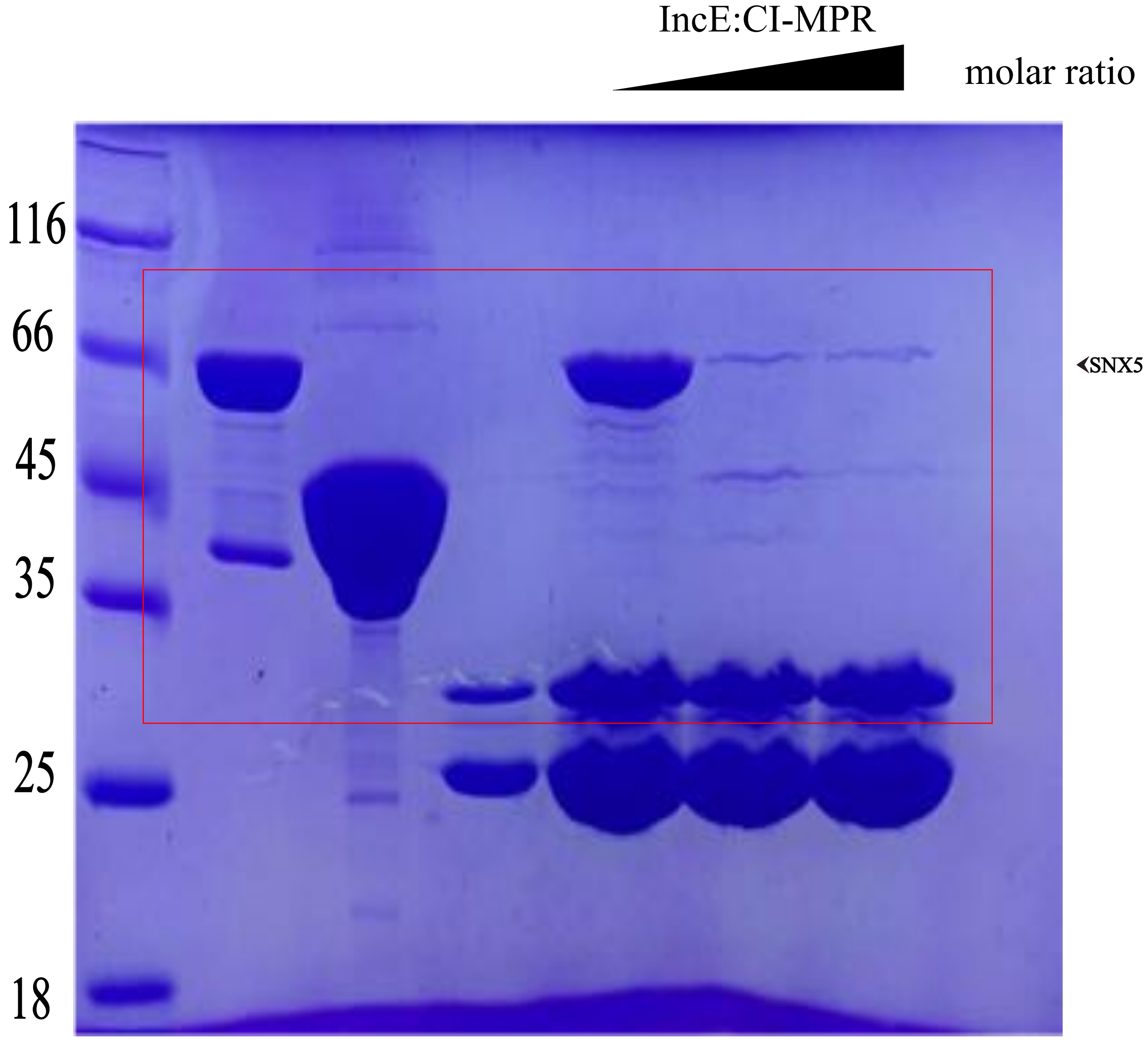

S4 Fig

S4A Fig

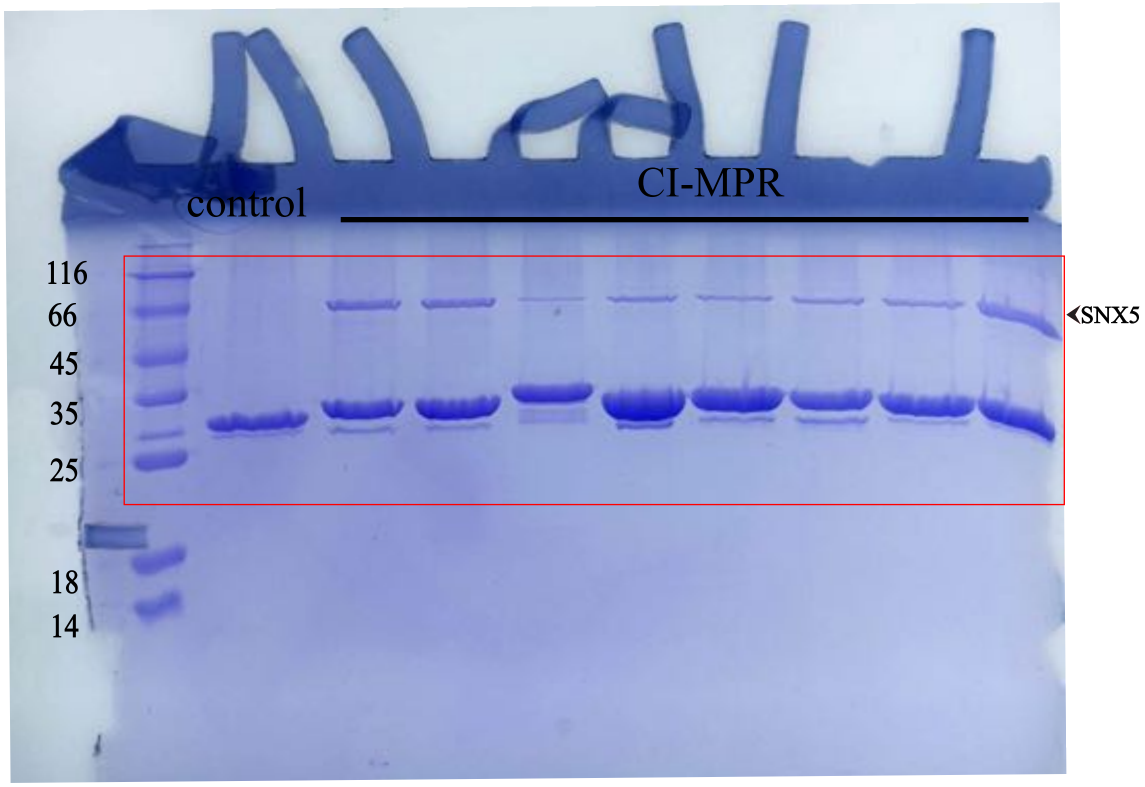

S4B Fig

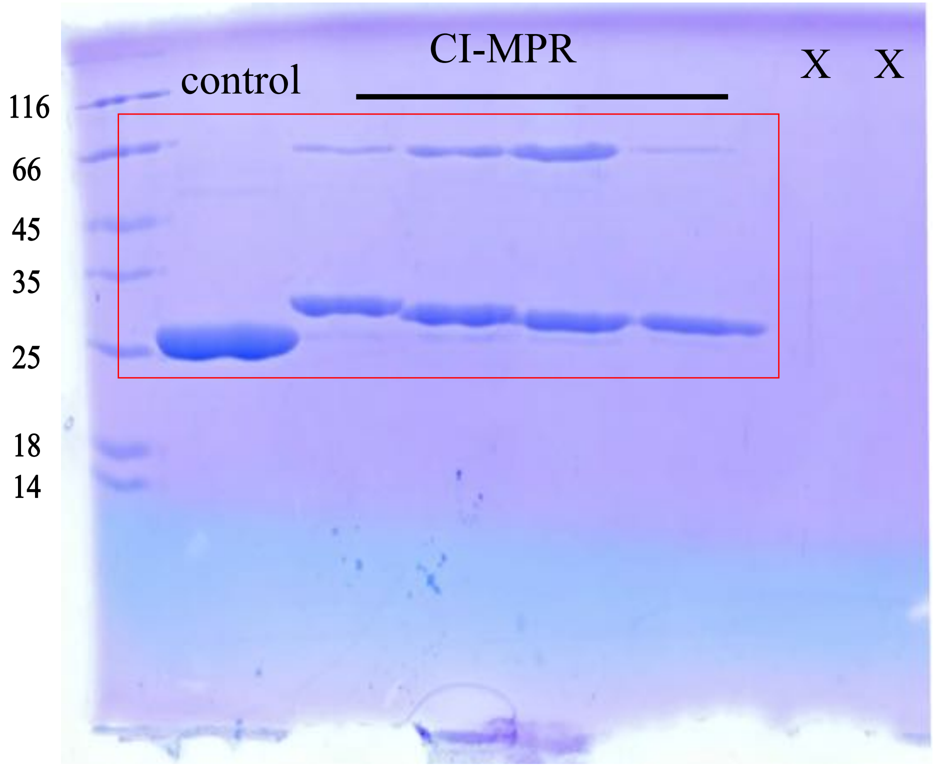

S4C Fig

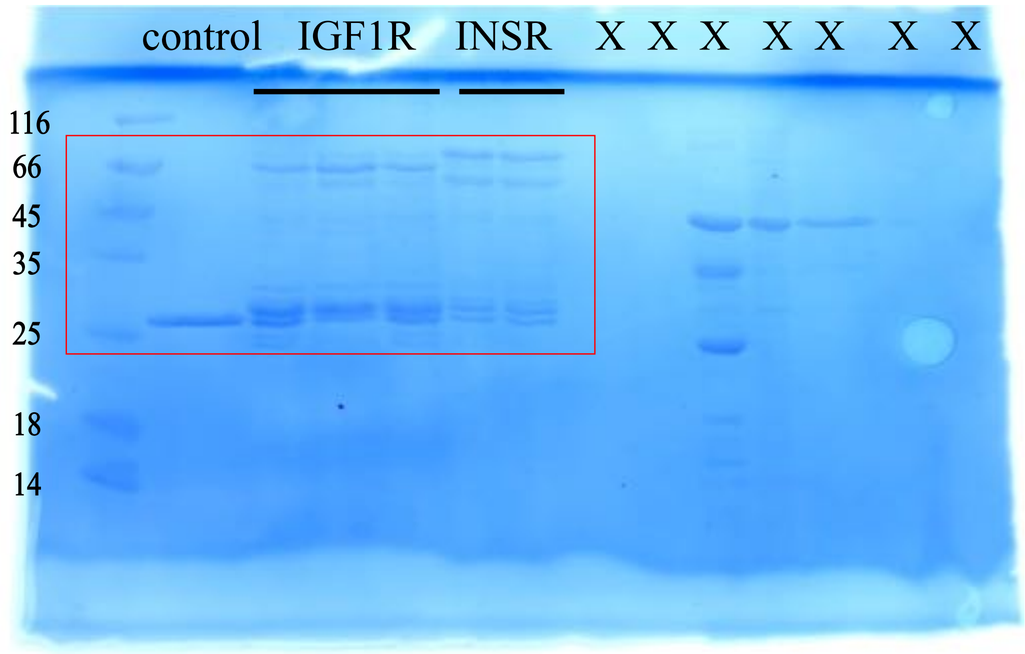

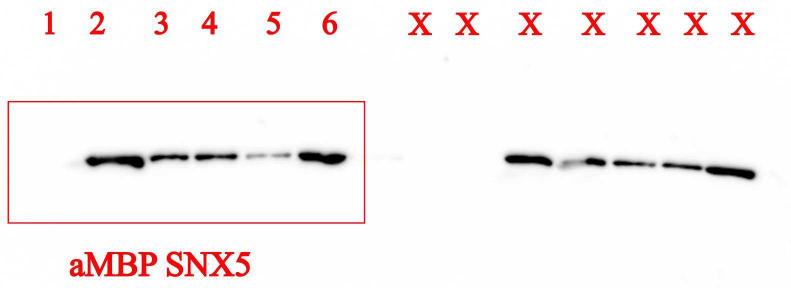

S5 Fig

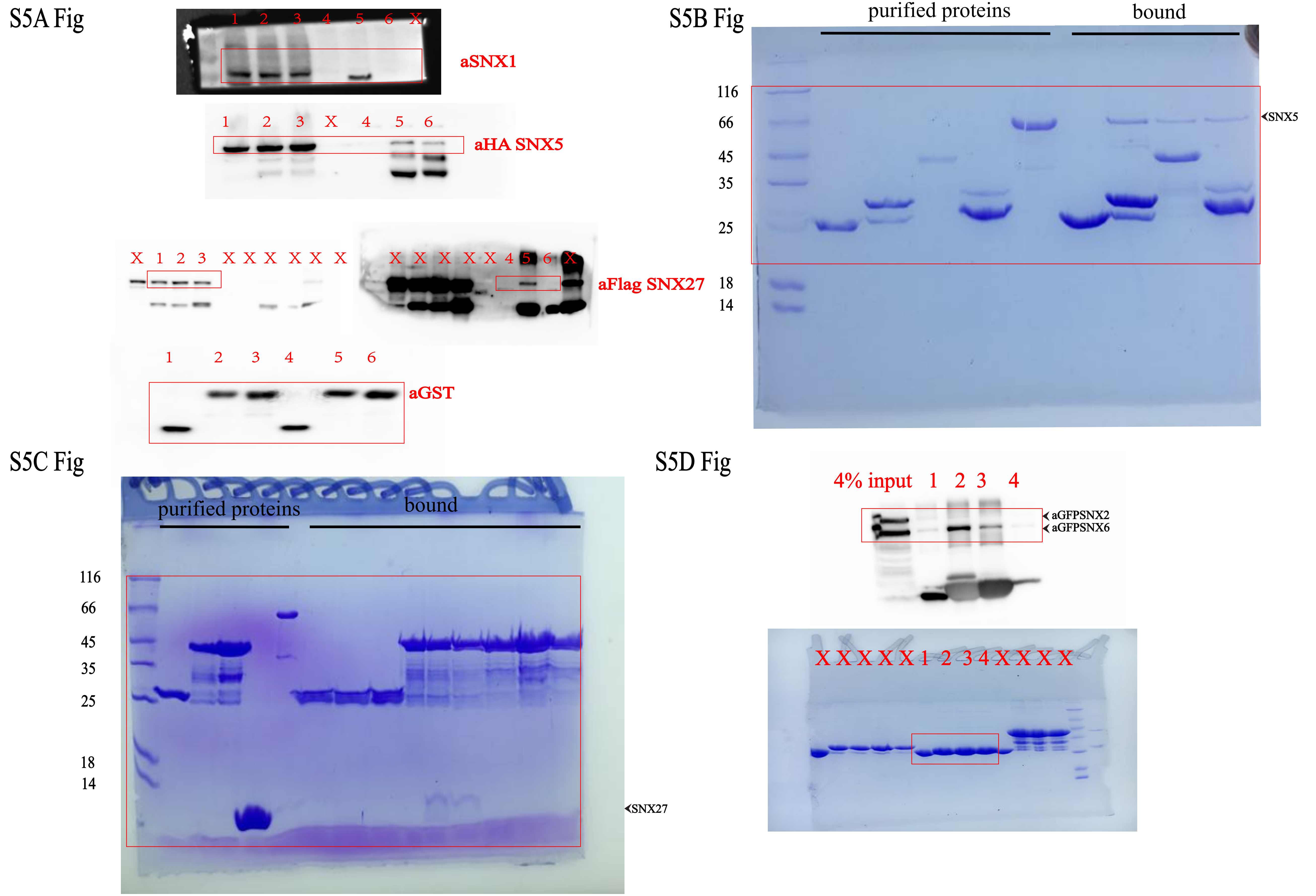
S5A Fig
1 2 3 4 5 6 X
aSNX1
1 2 3 X 4 5 6
aHA SNX5
X 1 2 3 X X X X X X
X X X X X 4 5 6 X
aFlag SNX27
1 2 3 4 5 6
aGST
S5B Fig
purified proteins
bound
116
66
45
35
25
18
14
SNX5
S5C Fig
purified proteins
bound
116
66
45
35
25
18
14
SNX27
S5D Fig
4% input 1 2 3 4
aGFPSNX2
aGFPSNX6
X X X X X 1 2 3 4 X X X X

S6 Fig

S6A Fig

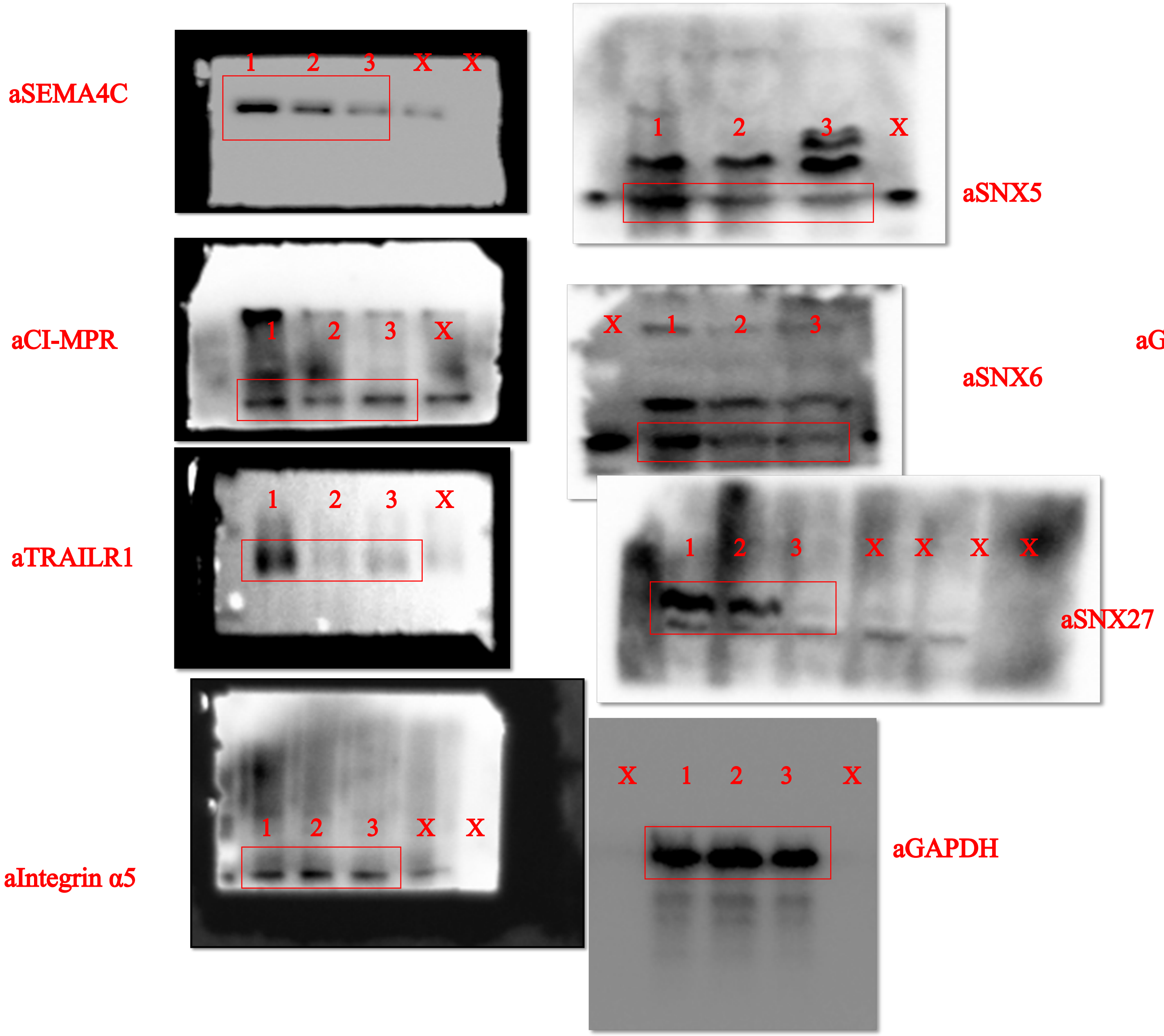

S6E Fig

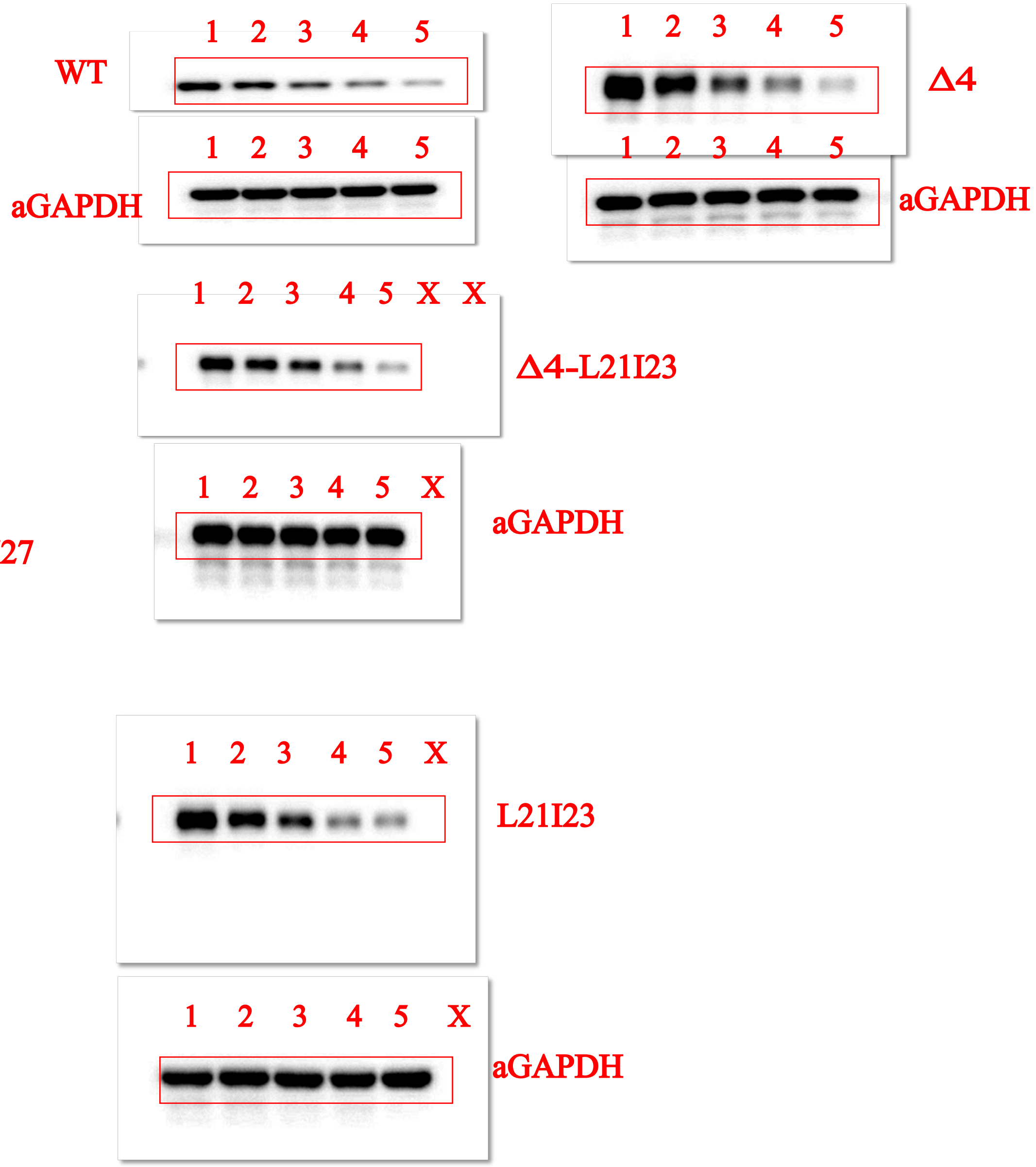

S8 Fig

S8A Fig

siSNX1256
siVPS35

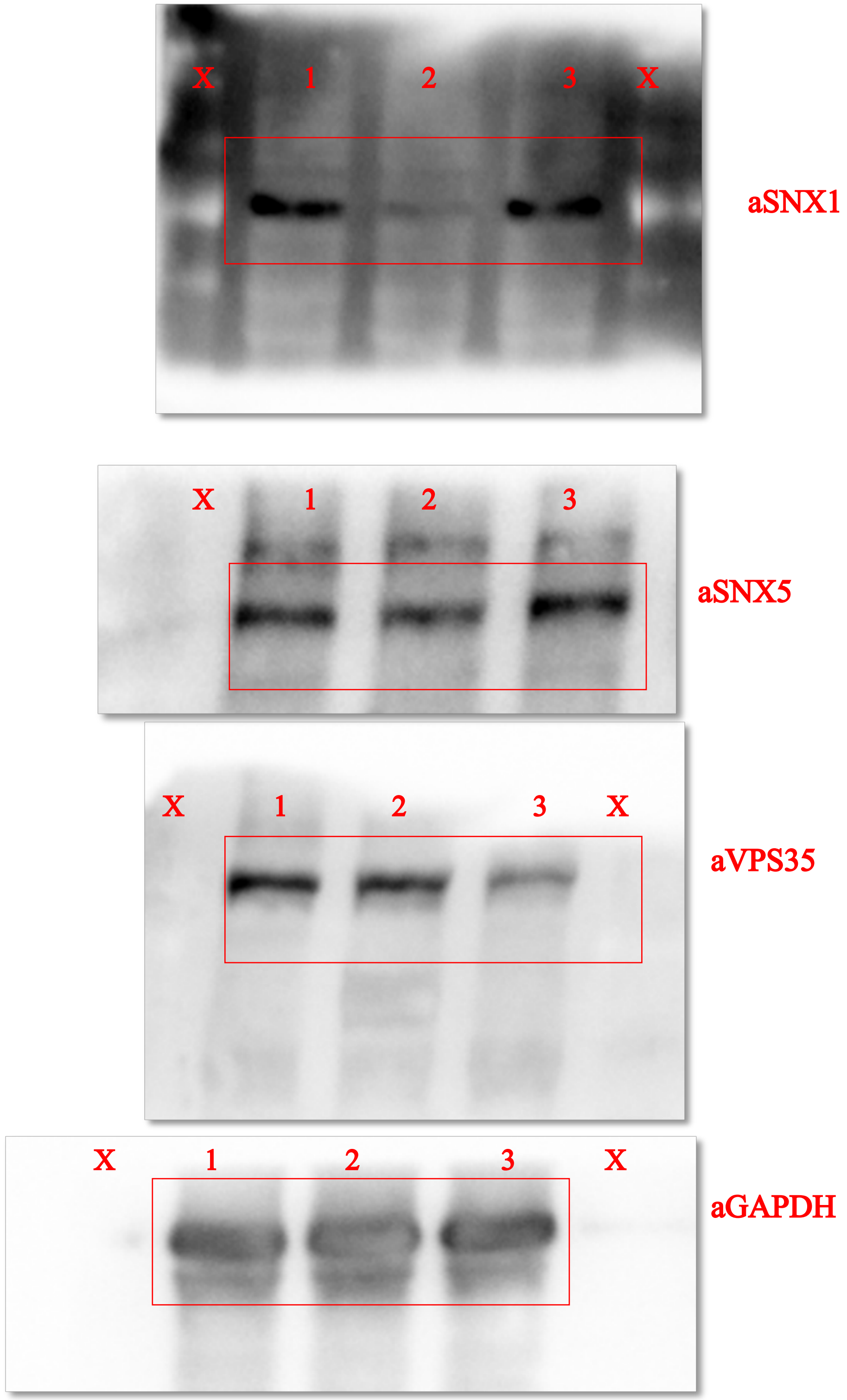

S8D Fig

SNX1+2 KO
SNX5+6 KO

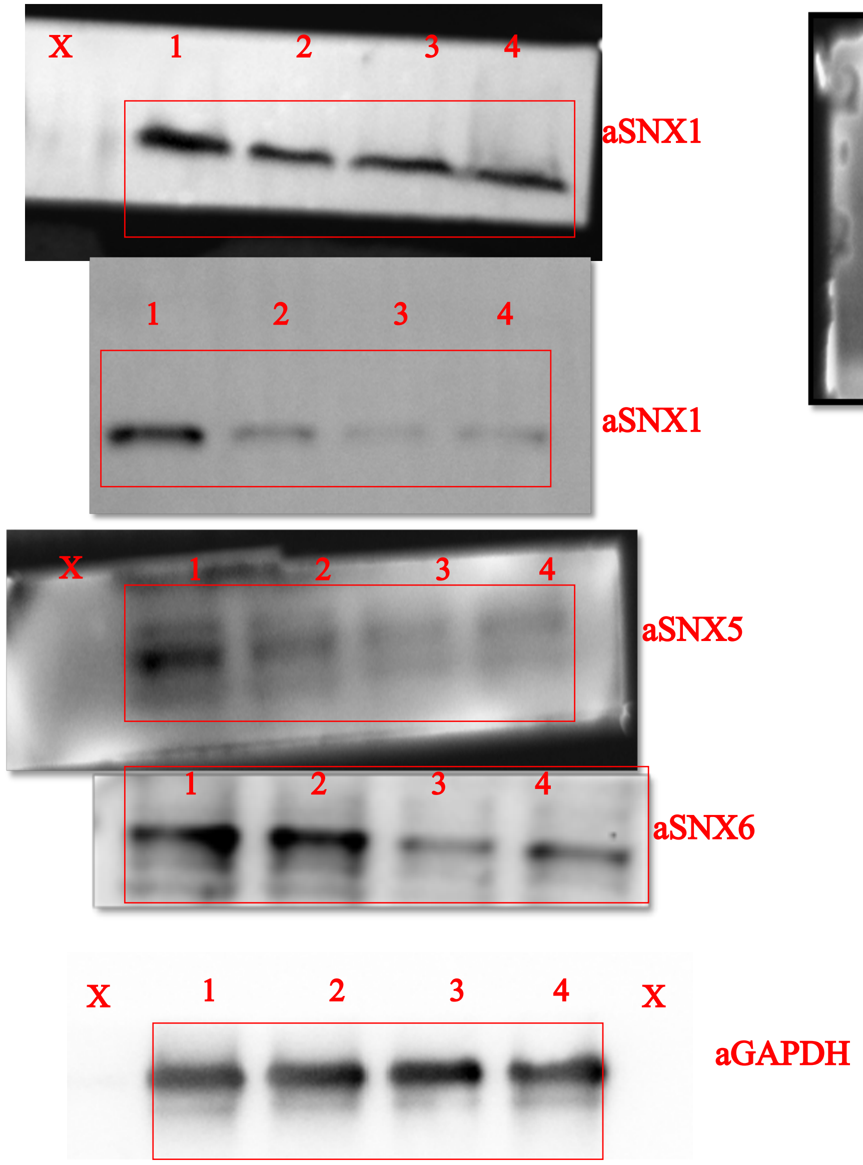

SNX27 KO

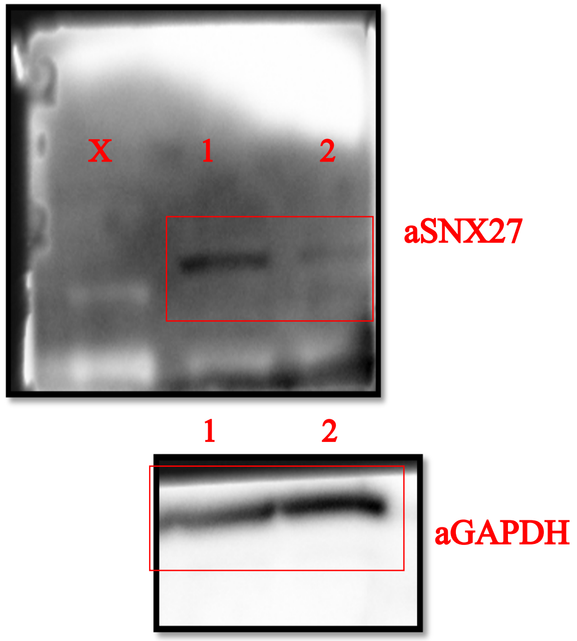

Supplement: S1 Raw images — (PDF) [file pbio.3000631.s014.pdf]
